# OpenFLUX2: ^13^C-MFA modeling software package adjusted for the comprehensive analysis of single and parallel labeling experiments

**DOI:** 10.1186/s12934-014-0152-x

**Published:** 2014-11-19

**Authors:** Mikhail S Shupletsov, Lyubov I Golubeva, Svetlana S Rubina, Dmitry A Podvyaznikov, Shintaro Iwatani, Sergey V Mashko

**Affiliations:** Ajinomoto-Genetika Research Institute, 117545 Moscow, Russian Federation; Computational Mathematics and Cybernetics Department, Lomonosov Moscow State University, 119991 Moscow, Russian Federation; Department of Theoretical and Experimental Physics, Moscow Physical Engineering Institute (Technical University), 115409 Moscow, Russian Federation; Biological Department, Lomonosov Moscow State University, 119991 Moscow, Russian Federation; Present address: Fermentation Group, Process Industrialization Section, Research Institute for Bioscience Products & Fine Chemicals, Ajinomoto Co., Inc., 840-2193 SAGA Saga-shi, Morodomi-cho, 450 Morodomitsu, Japan

**Keywords:** Non-linear least-squares minimization problem, Normalized flux precision function, Partial optimization of experimental design, Convergence control of flux confidence interval bounds

## Abstract

**Background:**

Steady-state ^13^C-based metabolic flux analysis (^13^C-MFA) is the most powerful method available for the quantification of intracellular fluxes. These analyses include concertedly linked experimental and computational stages: (*i*) assuming the metabolic model and optimizing the experimental design; (*ii*) feeding the investigated organism using a chosen ^13^C-labeled substrate (tracer); (*iii*) measuring the extracellular effluxes and detecting the ^13^C-patterns of intracellular metabolites; and (*iv*) computing flux parameters that minimize the differences between observed and simulated measurements, followed by evaluating flux statistics. In its early stages, ^13^C-MFA was performed on the basis of data obtained in a single labeling experiment (SLE) followed by exploiting the developed high-performance computational software. Recently, the advantages of parallel labeling experiments (PLEs), where several LEs are conducted under the conditions differing only by the tracer(s) choice, were demonstrated, particularly with regard to improving flux precision due to the synergy of complementary information. The availability of an open-source software adjusted for PLE-based ^13^C-MFA is an important factor for PLE implementation.

**Results:**

The open-source software OpenFLUX, initially developed for the analysis of SLEs, was extended for the computation of PLE data. Using the OpenFLUX2, *in silico* simulation confirmed that flux precision is improved when ^13^C-MFA is implemented by fitting PLE data to the common model compared with SLE-based analysis. Efficient flux resolution could be achieved in the PLE-mediated analysis when the choice of tracer was based on an experimental design computed to minimize the flux variances from different parts of the metabolic network. The analysis provided by OpenFLUX2 mainly includes (*i*) the optimization of the experimental design, (*ii*) the computation of the flux parameters from LEs data, (*iii*) goodness-of-fit testing of the model’s adequacy, (*iv*) drawing conclusions concerning the identifiability of fluxes and construction of a contribution matrix reflecting the relative contribution of the measurement variances to the flux variances, and (*v*) precise determination of flux confidence intervals using a fine-tunable and convergence-controlled Monte Carlo-based method.

**Conclusions:**

The developed *open-source* OpenFLUX2 provides a friendly software environment that facilitates beginners and existing OpenFLUX users to implement LEs for steady-state ^13^C-MFA including experimental design, quantitative evaluation of flux parameters and statistics.

**Electronic supplementary material:**

The online version of this article (doi:10.1186/s12934-014-0152-x) contains supplementary material, which is available to authorized users.

## Background

Metabolic flux analysis (MFA) plays a key role in systems biology because intracellular fluxes, i.e*.*, *in vivo* reaction rates through different pathways within an intact living cell [1], are the functional output of all conventional genetic and metabolic regulatory systems and determine the physiological phenotype of the cell [2].

In recent decades, a metabolic steady-state version of ^13^C-labeling-based MFA (^13^C-MFA) has become the best developed and most powerful method for quantifying intracellular fluxes when all fluxes and metabolite concentrations can be considered to be (at least approximately) constant [2-4]. ^13^C-MFA, applied to microbial, plant and mammalian systems, has been increasingly used in systems biology and metabolic engineering [5-7], biotechnology and medicine [8-10].

Due to the high complexity of native metabolic networks, ^13^C-MFA typically involves the use of a simplified stoichiometric model in which only the key pathway reactions of the central carbon metabolism and the set of lumped targeted biosynthetic reactions are parameterized before the assumed model-based fluxes are inferred from measurable quantities [11].

Concerning the experimental data applied in ^13^C-MFA, the physiological/extracellular fluxes or effluxes (e.g., biomass precursor drain, substrate uptake, and product excretion rates) and ^13^C-labeling patterns (i.e., isotopomer distributions) of metabolic products resulting from feeding partially ^13^C-labeled substrates (tracers) are used. These effluxes are determined from the time courses of cellular dry weight and extracellular metabolite concentrations during cultivation [12]. The ^13^C-isotopomers generated due to the metabolic conversion of tracers are detected through nuclear magnetic resonance (NMR) spectroscopy [13], mass spectrometry (MS) [14], and/or tandem MS (MS/MS) [15].

Prof. W. Wiechert and co-workers significantly contributed towards formalizing the framework for ^13^C-MFA: from measured effluxes and intracellular labeling information the intracellular fluxes could be computed [16-19]. On this basis, several mathematical models have been developed that can simulate a unique profile of isotopomer abundance for the fluxes with assigned parameters by describing the propagation of labeled atoms from the tracer through an assumed metabolic network according to the known atom rearrangements for each reaction [18,20-26]. All simulations are providing under the essential assumption that the possible isotopic mass effects [27] are negligible, i.e., that the labeling states of the metabolites do not influence the rate of their enzymatic conversion [16]. The goal of ^13^C-MFA is to determine the set of initially unknown flux parameters that minimizes the differences between experimentally observed and simulated measurements. In mathematical essence, this set is a solution of a large-scale non-linear parameter estimation problem [28]. Analytical solutions of this problem are available only for the simplest systems. Therefore, the values of the assumed fluxes must generally be inferred from the experimental datasets through computer model-based interpretation using an iterative least-squares fitting procedure [2,3,26].

Several high-performance computational software suites for performing flux calculations have been developed and described, e.g., 13CFLUX [29] and its reinforced version – 13CFLUX2 [30], METRAN [31,32], OpenFLUX [33], FIA [25], influx_s [34], OpenMebius [35].

These software toolboxes most often automatically generate metabolite and isotopomer balance models relying on an initially user-defined simple notation of metabolic networks and the known atom transitions occurring in biochemical reactions. Then, starting from the generated models and from measured effluxes that must be constrained within the obtained error ranges, semi-random guesses regarding intracellular fluxes are used to simulate *in silico*^13^C-labeling patterns of targeted metabolites, which, in turn, are compared with the measured patterns. This process is repeated until a satisfactory match to the measurable quantities is achieved, i.e., the constrained non-linear least-squares minimization problem (NLLSP) is solved [29]. According to the rules of regression analysis, providing a statistical goodness-of-fit test of the adequacy of the applied flux model is required, at a minimum, after determining the optimized fluxes [28,36]. Then, linearized statistics [17,37,38], a non-linear-based search algorithm [28], and/or the Monte Carlo approach [39,40] are used to estimate the precise flux resolution, i.e., the uncertainty of the determined fluxes. The optimized parameters of the fluxes and their confidence intervals in the statistically adequate user-made metabolic model must be obtained as the concerted results of these computations.

When the ^13^C-labeling data were obtained from NMR in the early stages of ^13^C-MFA development, each analysis was typically performed on a single labeling experiment (SLE), primarily for cost reasons [41]. Implementing highly sensitive MS- [42-44] and MS/MS-mediated [15,45] measurements, which are development approaches that involve ^13^C-tracer experiments at a miniaturized scale [46,47], led to a significantly increased accessibility and decreased cost of labeling experiments (LEs). Thus, it has become possible to realize the advantages of parallel labeling experiments (PLEs), in which two or more LEs are initiated from the same seed culture and conducted in parallel under the same experimental conditions differing only in the set of ^13^C-tracers applied [48-53].

SLE-based ^13^C-MFA remains a widely used method and can be implemented with the application of a single labeled substrate as a tracer or using a mixture of isotopomers of the same compound or multiple labeled substrates [32,54-57]. Studies have shown that achieving optimal resolution of fluxes from different parts of the central carbon metabolism requires different ^13^C-tracers [19,48,58]. Several sophisticated experimental design strategies have been adopted to improve the desired flux precision [19,32,36,49,54,56,59-64]. Therefore, the use of only one set of tracers will likely not maximize the resolution of all fluxes in SLEs, particularly when a large-scale metabolic model is employed [28,65].

According to previous studies [53,58,66,67], there are several advantages of using PLEs for ^13^C-MFA compared with an SLE-based approach. In general, the data from each LE are integrated to achieve an improved flux resolution, primarily due to the synergy of the complementary information used for fitting to the single metabolic model [48,53,58,66]. Indeed, the latest applications of the COMPLETE (short for COMplementary Parallel Labeling Experiments TEchnique [58]) MFA approach employing all six singly labeled glucose tracers to evaluate metabolic fluxes resulted in the most accurate and precise flux parameters obtained thus far for wild-type *E. coli* as well as for some metabolically engineered strains of the bacterium [58,67]. However, for laboratories lacking in-house experience, one crucial factor in the implementation of the PLE approach is the availability of a free, ready-to-use software package allowing the successful manipulation of the complex data obtained in PLEs, which is necessary for comprehensive flux analysis.

In the present study, the open-source software OpenFLUX [33], which uses an elementary metabolic unit (EMU) decomposition-based algorithm to generate an isotopomer balance model [26] and was initially developed for SLE analysis, has been extended for the computation of PLE data (see, Additional file 1: ***SF-1.3***. The methodology of PLE data implementation is rather clear, and one of the possible algorithms has been earlier schematically described in [53]. The expertized investigators have already adjusted their home-made ^13^C-MFA software by PLEs-mediated data (see, [66] for review). Currently, additional MATLAB-based scripts have been appeared on the OpenFLUX homepage (http://openflux.sourceforge.net) that demonstrated to users how the data of two labeling experiments conducting in parallel could be implemented in the already existing software. The presented *open-source* OpenFLUX2 provides a friendly software environment that facilitates beginners and existing OpenFLUX users to manipulate with SLE- and PLE-based data, for experimental design, determination of flux parameters, and for broaden evaluation of flux statistics.

Using OpenFLUX2, direct *in silico* simulation confirmed that the flux resolution was improved when ^13^C-MFA was provided with PLE data that were fitted to and integrated with the common metabolic model as compared with the individual analysis of each LE. Additionally, the best flux resolution was achieved in the analysis of PLE results when the choice of tracer for each provided LE was based on a computed experimental design targeted to minimize the approximated variances of several fluxes from the different parts of the assumed metabolic network. The statistical methods of analysis of the obtained experimental and simulated data, followed by a goodness-of-fit test of the adequacy of the applied metabolic model, have been extended in OpenFLUX2, including the statistical conclusions concerning the feasibility of the obtained flux parameters in the final report and the flux confidence intervals estimated at the desired significance level. In turn, the flux confidence intervals could be computed in OpenFLUX(2) using different methods, but up today the most dependable and precise approach is a fine-tunable Monte Carlo-based determination of the flux variances, which are dependent on the randomly corrupted measured data [33,39] and that has been modified in OpenFLUX2 due to implementation of a convergence control and visualization of computation results.

Following the original position of the OpenFLUX authors [33], OpenFLUX2, which is an extended version of the already available software, has been developed as *open-source software*. We hope that OpenFLUX2 will be useful to research groups applying ^13^C-MFA, particularly for beginners not yet experienced in fluxomics analyses. Additionally, the availability of the OpenFLUX2 *code* could promote further improvement of the software based on the experiences of different researchers. OpenFLUX2 can be downloaded from SourceForge (http://sourceforge.net/projects/openflux2).

## Results

### Key features of OpenFLUX2 software

OpenFLUX2 was developed as an extension of the OpenFLUX software. New calculation facilities were added, mostly as extensions of the initial options, without dramatic changes in the parent content. Moreover, the initial forms of the model and experimental data setup, together with results representation, were maintained as much as possible during the development of OpenFLUX2 to facilitate the transition from one version to the other. In the present study, the procedures that were developed previously in OpenFLUX and retained in OpenFLUX2 without essential modifications are indicated as “OpenFLUX(2)”, and only the added/modified elements are indicated as being implemented in OpenFLUX2.

To clarify the essence of the modifications implemented at the stage of OpenFLUX2 software development, the following items are schematically described in Additional file 1: (***SF-1.1*.**) the assignment of free fluxes, followed by (***SF-1.2.***) flux variability analysis; (***SF-1.3.***) the calculation of optimized fluxes through iterative fitting; (***SF-1.4.***) a goodness-of-fit analysis of the adequacy of the metabolic model; (***SF-1.5.***) local linearized statistical approximations; and non-linear-search of the optimal flux confidence intervals (***SF-1.6.***), where, in particular, the introducing a convenient concept, “the normalized flux precision” function, is described, as well; (***SF-1.7.***) – a fine tunable and convergence-controlled Monte Carlo-based approaches for precise determination of the optimized flux confidence intervals according to “discarding” strategy at the predetermined confidence level, significantly modified at the stage of implementation in OpenFLUX2. The main aim of this description is to demonstrate that the individual procedures are essential interconnected parts of a unique solution to a complex optimization problem, where the statistical significance of the calculated model-based parameters must be verified via the comprehensive goodness-of-fit of the model’s adequacy. As an auxiliary aim of this part, it is a rather short, but slightly (in comparison with the excellent review [4]) mathematically-enriched introduction in the ^13^C-MFA background that could be helpful, especially for beginners, to repair their knowledge by essential parts of linear algebra and statistics.

**Figure 1:**
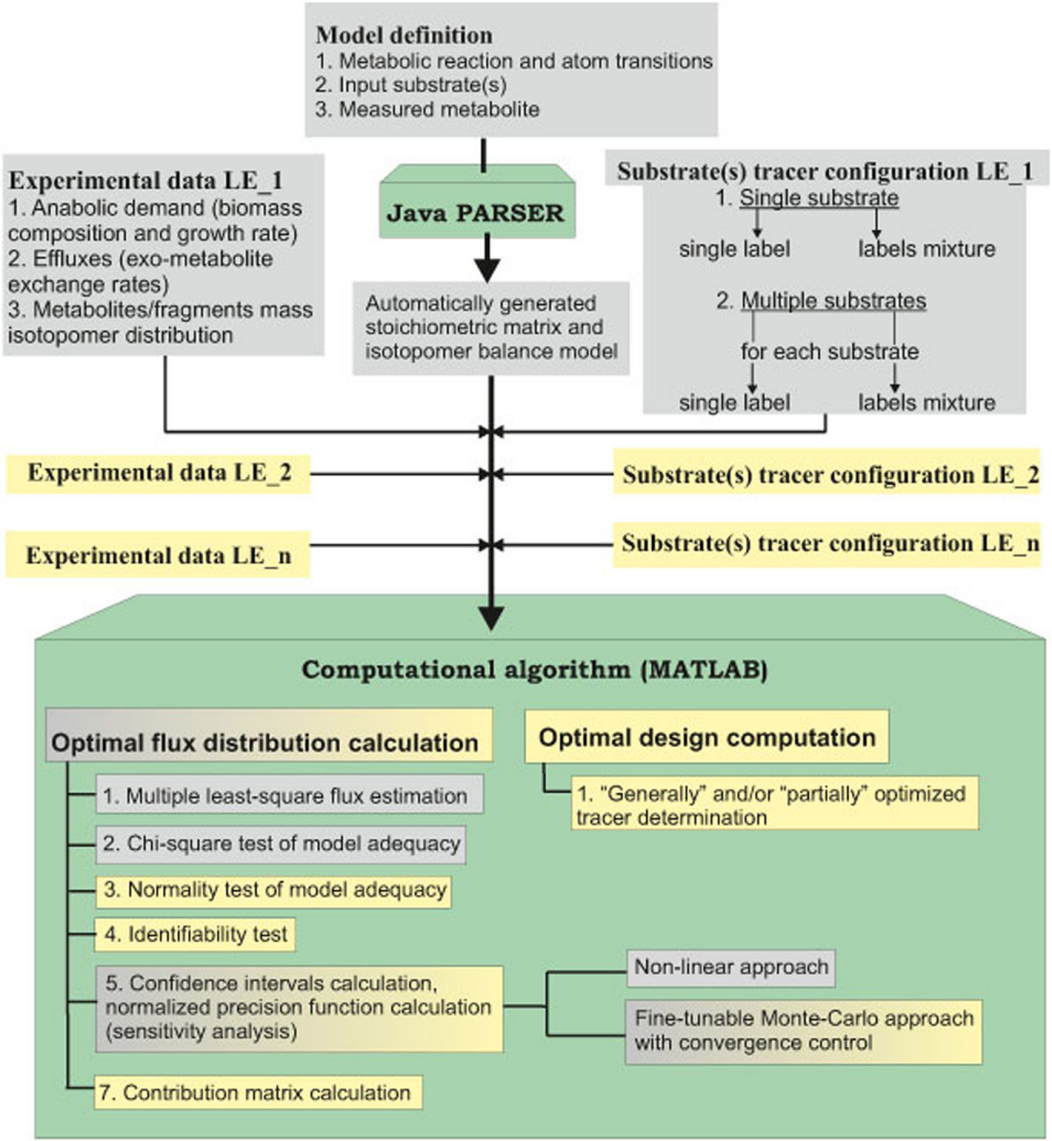
**Workflow of OpenFLUX2.** Green boxes represent the two main components of OpenFLUX(2): PARSER for automatic stoichiometric and isotopomer model generation and the MATLAB-based box for the ^13^C-MFA of the generated model. A gray background indicates options offered by OpenFLUX(2). A yellow background indicates options added during OpenFLUX2 development.

The workflow of OpenFLUX software consists of two components: (*i*) the automated set-up of stoichiometric and isotopomer balance models from user-supplied data, performed by Java PARSER, and (*ii*) the application of the generated models to the ^13^C-MFA of SLEs for flux parameter estimation and sensitivity analysis, performed by a set of specially developed MATLAB functions. The modifications introduced in OpenFLUX2 can be divided into two types (Figure 1). First, a set of new options related to statistical analysis of the flux evaluated from SLE data and experimental design facilities were added. Second, the new software was extended to perform metabolic flux analysis of PLE-based data. Here, the PLE concept is considered as determined in [66]; PLE-based design means that several tracer experiments that are started from the same seed culture to minimize biological variability, and must be performed independently under identical conditions on the same substrate(s), using different tracer(s).

Because an ^13^C-MFA PLE-based approach requires the simultaneous fitting of several datasets obtained from independent LEs to a single model, there is no major difference in the spread-sheet model set up and the consequent automated generation of stoichiometric and isotopomer balance models by the Java PARSER for PLEs and SLEs in OpenFLUX2. The set of substrates is fixed during the model generation step, and individual substrate tracer configurations are then defined by the user for each LE constituting the PLE together with the corresponding measured data (Figure 1). The option to use either a single label or a labeling mixture for each substrate in the PLE is provided by OpenFLUX2, as was previously provided in OpenFLUX for SLEs. Thus, all of the introduced modifications were finally concentrated in the MATLAB-based portion of the computational algorithm.

### Comprehensive flux analysis of a *Corynebacterium glutamicum* model, as an example, using OpenFLUX2 software

#### The *C. glutamicum* model

**Figure 2:**
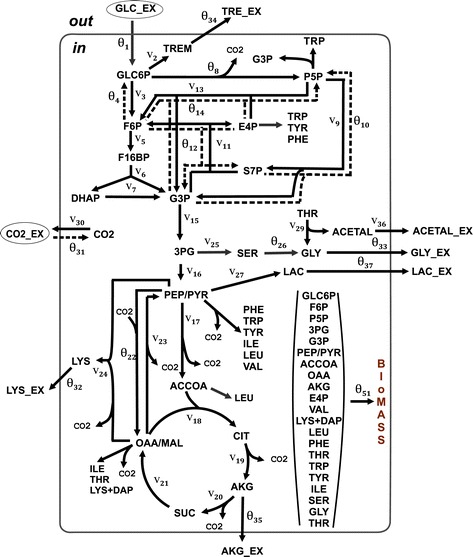
**Assumed metabolic model of an** **l** **-lysine-producing** ***Corynebacterium glutamicum*** **strain grown on glucose.** Single (solid) and double (solid and dotted) lines with arrows correspond to the assumed irreversible reactions and to bi-directional (forward and reverse) reactions, respectively. The letter “θ” indicates fluxes assigned as free, and the letter “v” indicates dependent fluxes. The free and dependent fluxes are numbered continuously throughout the applied model. Amino acid biosynthetic pathways are shown schematically as the drain of precursors of the corresponding amino acid. Carbon sources are indicated with ovals. The following abbreviations are used for metabolites: 3PG – 3-phosphoglycerate; ACCOA – acetyl-CoA; ACETAL – acetaldehyde; ACETAL_EX – extracellular acetaldehyde; AKG – α-ketoglutarate; AKG_EX – extracellular α-ketoglutarate; CIT – lumped pool of citrate and isocitrate; CO2 - carbon dioxide; CO2_EX – extracellular carbon dioxide; DAP – diaminopimelate; DHAP – dihydroxyacetone phosphate; E4P – d-erythrose 4-phosphate; F16BP – fructose 1,6-bisphosphate; F6P – d-fructose 6-phosphate; G3P – glyceraldehyde 3-phosphate; GLC_EX – extracellular glucose; GLC6P – d-glucose 6-phosphate; GLY_EX – extracellular glycine; LAC - lactate; LAC_EX – extracellular lactate; LYS_ EX – extracellular lysine; MAL – malate; OAA – oxaloacetate; P5P – pentose-5-phosphates (lumped pool of d-ribulose 5-phosphate, d-ribose 5-phosphate, d-xylulose 5-phosphate); PEP – phosphoenolpyruvate; PYR – pyruvate; S7P – d-sedoheptulose 7-phosphate; SUC – succinate; TREM – trehalose constituent (d-glucose 6-phosphate and UDP-glucose); TRE_EX – extracellular trehalose. Amino acids are expressed using the standard three-letter code.

The specific features of the developed OpenFLUX2 software were illustrated via the *in silico* simulation of a set of SLEs and PLEs followed by their comprehensive ^13^C-MFA. The *Corynebacterium glutamicum* metabolic model initially proposed for an l-lysine-overproducing *C. glutamicum* strain by Becker et al. [68] and later used to demonstrate the possible applications of OpenFLUX software [33] was chosen (with minor changes; see Methods) as the object of the computational simulation and ^13^C-MFA. This model, schematically presented in Figure 2, accounted for the main central metabolic pathway, the biosynthetic pathways for several excreted products, including lysine as the main product, and anabolic demand. CO_2_-mediated carbon transfer was accounted for using expression reactions, accompanied by CO_2_ production/consumption, in an explicit manner. The bi-directional reactions were represented as non-negative forward and reverse fluxes. Finally, the metabolic model was composed of 51 unknown fluxes balancing 36 metabolites, resulting in the stoichiometric matrix **S**, dim(**S**) = (36 × 51), with a residual 15 degrees of freedom. The degrees of freedom were associated with the following free fluxes: experimentally determined effluxes, including biomass biosynthesis (1 flux), effluxes of secreted products (5 fluxes), and the glucose uptake rate (1 flux); reverse branches of five bi-directional reactions; and free irreversible reactions associated with branch points. An isotopomer model was generated automatically under the assumption that the mass isotopomer distributions of the following metabolites were measured: alanine, valine, threonine, aspartate, glutamate, serine, phenylalanine, glycine, tyrosine, and trehalose. Application of the EMU approach offered by OpenFLUX(2) led to a major simplification of the isotopomer model (see Methods).

#### Circumstantiation of flux parameters for the computer simulations

The full set of fluxes necessary for the simulation of *in silico* SLEs and PLEs for the defined model was unfortunately not directly available from previously published materials [33,68]. Thus, the evaluation of fluxes from published experimental data was repeated in the present study. To this end, a new constrained NLLSP was designed based on a modified metabolic model, with assigned free fluxes, previously measured [68] effluxes with their variances, and MIDs with variances assumed to be 0.15 mol% (see Methods). Solutions of the designed NLLSP were obtained by OpenFLUX2 using a gradient-based local optimization method with the assistance of MATLAB’s FMINCON function (see Additional file 1: ***SF-1.3*.**, for details). To determine the global minimum, 100 independent iterative trials were performed with an arbitrary set of initial free fluxes, **θ**_k_ ∈ ℜ^*p*^, *k* = 1, 2, …, 100, where ℜ^*p*^ indicates a feasible constrained domain in the free flux variation space (see Additional file 1: ***SF-1.2***.). These trials resulted in a set of flux estimates, $\mathbf{u}\left({\overset{\frown }{\boldsymbol{\uptheta}}}_k\right),k=1,2,\dots, 100$, corresponding to the minimum values of the objective function ${\left(SS{R}_{\mathbf{f}}^{SLE}\right)}_k$ in Eq. (*S* − 1.3.7), which were reached in each *k*-th trial. According to the optimization report, all of the provided trials were terminated successfully due to the achievement of the given termination tolerance (ΤΤ = 1 × 10^− 4^) within the constraint violation tolerance. The χ^2^-based goodness-of-fit confirmed the model adequacy (see Additional file 1: ***SF-1.4*.**, for details) for 82 (at a significance level of *α* = 0.05) and 34 (at *α* = 0.32) of the 100 obtained values of ${\Xi}_{\mathrm{k}}=2\cdot {\left(SS{R}_{\mathbf{f}}^{SLE}\right)}_k$ as follows:

1 $$ {\chi}_{\alpha /2}^2\left(W-p\right)<{\Xi}_k<{\chi}_{1-\alpha /2}^2\left(W-p\right) $$

where *W* is the number of independent measurements, and *p* is the number of estimated free fluxes ((*W* − *p* = 21) in this case). All essential information concerning the obtained solutions is presented in Table 1**(Exp_1.1),** Additional file 2: Figure SF-2.1, and Additional file 3: Table SF-3.3. A minimal value of the Ξ function was registered for the group of 26 trials, and all fluxes from these solutions corresponded practically to the same point, $\mathbf{u}\left(\overset{\frown }{\boldsymbol{\uptheta}}\right)\in {\Re}^n$, in the feasible *n*-dimensional space for the flux variation.

**Table 1:** **General characteristics of the obtained NLLSP solutions**

**Characteristic**	**Exp 1.1**	**Exp 1.2**	**Exp 2.1**	**Exp 2.2**	**Exp 3.6**	**Exp 3.1**	**Exp 4.1**	**Exp 4.2**	**Exp 4.3**	**Exp 5.1**	**Exp 5.2**	**Exp 5.3**	**Exp 5.4**	**Exp 5.5**	**Exp PLE_1 (5.1-5.5)**
**Number of measurements,** *W* ^**(a)**^	**36**	**36**	**45**	**45**	**45**	**45**	**45**	**42**	**41**	**56**	**61**	**66**	**59**	**48**	**290**
**Number of free fluxes,** *p* ^**(b)**^	**15**	**15**	**15**	**15**	**15**	**15**	**15**	**15**	**15**	**15**	**15**	**15**	**15**	**15**	**15**
**Degrees of the freedom,** *W-p*	**21**	**21**	**30**	**30**	**30**	**30**	**30**	**27**	**26**	**41**	**46**	**51**	**44**	**33**	**275**
**Termination tolerance,** TT	**1 × 10** ^**−4**^	**1 × 10** ^**−6**^	**1 × 10** ^**−4**^	**1 × 10** ^**−6**^	**1 × 10** ^**−4**^	**1 × 10** ^**−4**^	**1 × 10** ^**−4**^	**1 × 10** ^**−4**^	**1 × 10** ^**−4**^	**1 × 10** ^**−4**^	**1 × 10** ^**−4**^	**1 × 10** ^**−4**^	**1 × 10** ^**−4**^	**1 × 10** ^**−4**^	**1 × 10** ^**−4**^
**Minimal reached value of** Ξ(Ξ_min_)	**18.2**	**18.2**	**1.5 × 10** ^**−3**^	**1.5 × 10** ^**−3**^	**9.65**	**22.8**	**46.6**	**38.9**	**18.9**	**23.2**	**24.6**	**50.4**	**44.4**	**31.5**	**239**
**Maximal reached value of** Ξ(Ξ_max_)	**8.5 × 10** ^**4**^	**263.1**	**105.6**	**1.4 × 10** ^**4**^	**3.5 × 10** ^**4**^	**1.5 × 10** ^**4**^	**1.4 × 10** ^**4**^	**1.4 × 10** ^**4**^	**1.1 × 10** ^**4**^	**23.7**	**28.8**	**53.9**	**983**	**910.5**	**9.0 × 10** ^**3**^
**Number of** ${\Xi}_{\mathrm{k}}:{\varXi}_k<{\chi}_{0.025}^2\left(W-p\right)$	**0**	**0**	**98**	**99**	**92**	**0**	**0**	**0**	**0**	**100**	**100**	**0**	**0**	**0**	**0**
**Number of** ${\Xi}_k:{\chi}_{0.025}^2\left(W-p\right)<{\Xi}_k<{\chi}_{0.975}^2\left(W-p\right)$	**82**	**85**	**0**	**0**	**6**	**96**	**26**	**47**	**97**	**0**	**0**	**100**	**99**	**99**	**97**
**Number of** ${\Xi}_k:{\chi}_{0.16}^2\left(\mathrm{W}-\mathrm{p}\right)<{\Xi}_k<{\chi}_{0.84}^2\left(W-p\right)$	**34**	**85**	**0**	**0**	**2**	**96**	**0**	**0**	**97**	**0**	**0**	**100**	**99**	**99**	**0**
**Number of trials with** Ξ_*k*_ : Ξ_*k*_ = Ξ_min_^**(c)**^	**26**	**77**	**6**	**51**	**28**	**11**	**4**	**4**	**7**	**94**	**89**	**96**	**96**	**82**	**91**
$\mathrm{Null}\left({\mathbf{J}}_{\mathbf{f}}\left(\overset{\frown }{\boldsymbol{\uptheta}}\right)\right)$ ^(d)^	**Empty**	**Empty**	**Empty**	**Empty**	**Empty**	**Empty**	**Empty**	**Empty**	**Empty**	**Empty**	**Empty**	**Empty**	**Empty**	**Empty**	**Empty**
**Individual Residuals ∈ N(0, 1)** ^(d, e)^	**Yes**	**Yes**	**No**	**No**	**Yes**	**Yes**	**No**	**No**	**Yes**	**Yes**	**Yes**	**Yes**	**Yes**	**Yes**	**Yes**

To determine the global minimum of the NLLSP, 100 independent iterative trials were provided with an arbitrary set of initial free fluxes for all represented *in silico* LEs. All iterative trials were terminated successfully for each independent NLLSP. More detailed characteristics for all performed *in silico* experiments are listed in Additional file 3.

^(a)^In the case of the SLE, the vector of the measured data consists of both measured MID values and measured effluxes. In the case of the PLE, the vector of the measured data consists of the measured MIDs and effluxes that were available for each SLE.

^(b)^The measured effluxes are included in the set of free fluxes, constrained according to Eq. (*S* − 1.2.2) with experimentally determined parameters ${\mathbf{q}}_{eff}^{mea},{\boldsymbol{\upsigma}}_{eff}^{mea}$.

^(c)^The values of Ξ_*k*_ that were obtained during different iterative trials were assumed to be equal to the Ξ_min_ if the values were different from the Ξ_min_ by a value less than or equal to the TT level, i.e., Ξ_*k*_ − Ξ_min_ ≤ TT.

^(d)^The analysis was performed for the NLLSP solution, which provided the minimal achieved value of Ξ, i.e., Ξ_min_.

^(e)^The individual variance-weighted residuals were recognized to be not distributed as N(0, 1) if both the Kolmogorov-Smirnov test, provided by MATLAB, rejected this hypothesis and the individual residual plot revealed unpredictable high values for the individual residual/group of the individual residuals or, in contrast, unpredictable small values for the primary part of the individual residuals.

The flux identifiability analysis, which was based on model linearization and on the computation of $\mathrm{Null}\left({\mathbf{J}}_{\mathbf{f}}\left(\overset{\frown }{\boldsymbol{\uptheta}}\right)\right)$ in Eq. (*S* − 1.5.9) (see Additional file 1: ***SF-1.5***.), was provided for the obtained statistically available solution as described by Yang et al. [69]. This analysis resulted in the conclusion that only a unique set of flux parameters for the global minimum of the constrained NLLSP could be computed numerically; because the calculated null space matrix was empty. The goodness-of-fit analysis was finalized by confirming the Ν(0, 1) distribution of the individual variance-weighted residuals in $\Xi \left(\overset{\frown }{\boldsymbol{\uptheta}}\right)$ (see Additional file 1: ***SF-1.4.*** for details).

The obtained statistically acceptable solution of the constrained NLLSP, $\mathbf{u}\left(\overset{\frown }{\boldsymbol{\uptheta}}\right)$, primarily coincided with the previously published [33,68] values of fluxes in the range of the earlier evaluated flux confidence intervals (Additional file 2: Figure SF-2.2). Thus, all flux parameters, including previously unavailable, were evaluated and assigned as the true values for the assumed metabolic model, **u**(**θ**_*true*_).

**Figure 3:**
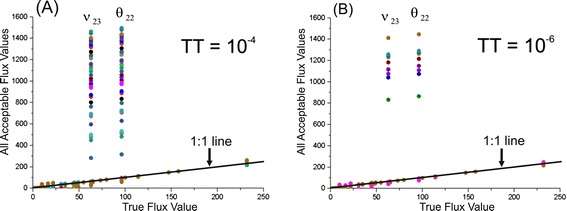
**Comparison of true flux values with flux parameters corresponding to all** ***χ*** ^**2**^ **–statistically acceptable values of the objective function.** The fluxes that successfully coincide with true values lie on the 1:1 line when plotted against the true flux. The fluxes estimated from the same iterative trial are indicated by the same color. **(A)** The termination tolerance, TT, is equal to the default value of 10^−4^; **(B)** TT =10^−6^.

Other *χ*^2^ -statistically acceptable trials resulted in larger values of Ξ(**θ**), which corresponded to the points of **u**(**θ**) ∈ ℜ^n^ and primarily differed from **u**(**θ**_true_) in the values of two fluxes: *θ*_22_: PYR + CO2→^F^ OAA and (v_dep_)_23_: OAA →^F^ PYR + CO2 (Figure 3(A)). The value of the residual net flux for these reactions, *v*^*net*^ = |*θ*_22_ − (*v*_*dep*_)_23_|, remained practically constant for all *χ*^2^ -statistically acceptable trials. The possible existence of local minima of the Ξ(**θ**) function with a parameter of *θ*_22_, as an alternative to its true values, was tested. As shown in Additional file 2: Figure SF-2.3, the minimized sum of the squared residuals as a function of *θ*_22_ represents one distinct global minimum in the neighborhood of (*θ*_22_)_*true*_ followed by a gentle decreasing slope, which ends nearly as a plateau.

Thus, additional solutions of the constrained NLLSP may be obtained in independent trials starting from the random initial values of the free fluxes, resulting in the premature termination of the computer search for the global minimum due to the discovery of a termination tolerance (TT). Indeed, repeated numerical solutions of the same constrained NLLSP under conditions with a decreasing TT of the objective function value sloped from the default value of 1 × 10^− 4^ up to the more precise value of ΤΤ = 1 × 10^− 6^ (see Additional file 1: ***SF-1.3.***, for details), resulting in an increased number of trials that reached the global minimum (77 of 100, in contrast to 26 in the previous calculations; see Table 1**(Exp_1.1; Exp_1.2)** and Additional file 2: Figure SF-2.1), with a decreasing number of alternative statistically acceptable solutions (Figure 3(B)).

Thus, a unique optimal set of fluxes for the statistically adequate metabolic model was obtained: $\mathbf{u}\left(\overset{\frown }{\boldsymbol{\uptheta}}\right)\equiv \mathbf{u}\left({\boldsymbol{\uptheta}}_{true}\right)$. Then, new GC-MS-based “experimental data,” i.e., new MIDs, could be generated through direct *in silico* simulation describing the propagation of ^13^C atoms from different tracers through a metabolic network with known flux parameters (see Methods).

#### ^13^C-MFA for *in silico* SLEs with [1-^13^C]-glucose as a tracer

Initially, the simulation was provided for 99% of [1-^13^C]-glucose as a tracer. The new set of MIDs was generated as described in Methods, excluding the last step of data corruption. To confirm that flux estimations could be unambiguously inferred from the obtained non-noised data, these simulations were used as the experimental data, with assumed MID variances equal to 0.4 mol%, and the assignment of all effluxes as variable free fluxes constrained in the range of the 95% confidence intervals with the standard deviations determined in [68] (see Additional file 3: **Table SF-3.3**). The solution to the corresponding constrained NLLSP was obtained using OpenFLUX2 software according to the standard procedure described above with details presented in Table 1**(Exp_2.1)**. In total, 98 values of Ξ_i_, which corresponded to solutions from the total obtained set of $\mathbf{u}\left({\overset{\frown }{\boldsymbol{\uptheta}}}_i\right),i=1,2,\dots, 100$, were smaller than the upper, and even the lower, critical threshold values, ${\chi}_{0.975}^2\left(W-p\right)$ and ${\chi}_{0.025}^2\left(W-p\right)$, respectively, at the 95% confidence level and with (W − p) = (38 + 7) − (8 + 7) = 30 degrees of freedom. Moreover, the group of trials (6 solutions at ΤΤ = 1 × 10^− 4^ and 51 solutions in the case of TT decreased to 1 × 10^− 6^; see Table 1**(Exp_2.1; Exp_2.2)**) had a minimal value of 1.5 ⋅ 10^− 3^, which was significantly less than the minimal threshold. Such a questionably small value for the objective function generally indicates possible overfitting of the applied model. However, in our case, the cause stems from the solution of the inverse task without corrupting the “experimental” data generated at the stage of direct simulation. The same cause resulted in the negative evaluation of the individual weighted residuals in Ξ_i_ according to the normality test, and the null hypothesis (concerning Ν(0, 1) distribution of residuals) was rejected. The flux estimates that corresponded to the solutions of the group with the minimal Ξ_i_ that was reached were assigned as reference fluxes, i.e., **u**(**θ**_*ref*_). Confirmation of flux identifiability was obtained based on the empty null space for the Jacobian matrix calculated at point **θ** = **θ**_*ref*_. As shown in Additional file 3: Table SF-3.5, the obtained reference flux values are extremely close to the true values used for data generation.

The next stage of the simulation was the generation of “experimental data” that were closed to results that could be obtained in real experiments. The values of the previously simulated MIDs and the measured effluxes were corrupted with the Ν(**0**, **σ**^*mea*^) normally distributed random errors using the Statistics Toolbox of MATLAB. Five (*L* = 5) obtained sets of data, ${\mathbf{x}}_i^{m\mathrm{e}\mathrm{a}}={\left({x}_1^i,{x}_2^i,\dots, {x}_{\mathrm{w}}^i\right)}^{\mathrm{T}},i=1,2,\dots, L$ were used for calculation of mean, and unbiased estimator of the variance, respectively:

2 $$ {\mu}_j^{mea}=\frac{1}{L}\cdot {\displaystyle \sum_{i=1}^{\mathrm{L}}{x}_j^i},\ {\sigma}_j^{mea}=\sqrt{\frac{1}{\left(L-1\right)}\cdot {\displaystyle \sum_{i=1}^{\mathrm{L}}{\left({x}_j^i-{\mu}_j^{mea}\right)}^2}} $$

for each from *w*_*MID*_ MIDs and *w*_*eff*_ effluxes, (*w* = *w*_*MID*_ + *w*_*eff*_), and to assign the ${SSR}_{\mathbf{f}}^{SLE}$ objective function that finally determines the constrained NLLSP (S − 1.3.8). Statistically acceptable solution (corresponding to the value 9.65 of Ξ(**θ**) function (for the group of 27 from 100 performed trials) that was smaller even the lower threshold, $\left({\chi}_{0.025}^2(30)=16.79\right)$) at the 95% confidence level, with Ν(0, 1) distributed weighted residuals, was found using OpenFLUX2 (Table 1**(Exp_3.6)**).

#### Monte Carlo-based and non-linear approaches for determination of flux confidence intervals

**Figure 4:**
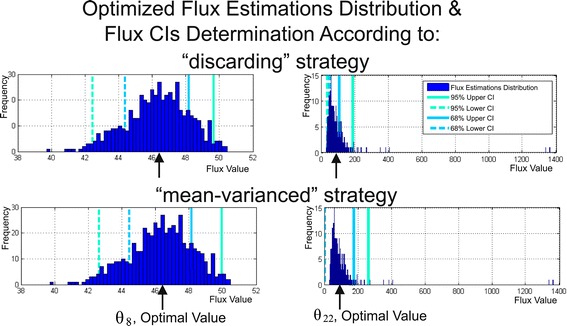
**Determination of flux confidence intervals at a confidence level of** ***γ*** **using the Monte Carlo approach based on multiple parameter estimations.** For each of *L*_*MAX*_ = 500 trials (simulations), all MIDs and measured efflux values were corrupted by random noise with a given standard deviations, and fluxes were estimated via least-squares optimization. Then, the estimations of each flux obtained in *L*_*MAX*_ trials were sorted in ascending order followed by performing of “discarding” or “mean-varianced” strategies for determination of flux confidence intervals at the confidence level of *γ*, $C{I}_{\gamma}^{MC-1}$ or $C{I}_{\gamma}^{MC-2}$, respectively, (see Additional file 1: ***SF-1.7.***). The computation of the 68% and 95% confidence interval bounds for the ${\overset{\frown }{\theta}}_8$ and ${\overset{\frown }{\theta}}_{22}$ fluxes provided by both Monte Carlo-based strategies are presented as examples of estimates that are distributed rather symmetrically or non-symmetrically, respectively, around the optimized values of fluxes (indicated by the cursor).

At the final stage of the ^13^C-MFA performed for the considered above constrained NLLSP, accurate 68% and 95% confidence intervals ($C{I}_{0.68}^{MC}$ and $C{I}_{0.95}^{MC}$, respectively) were computed for the optimized fluxes initially by fine-tunable and convergence-controlled Monte Carlo-based approach implemented in OpenFLUX2 for this purpose (see Additional file 1: ***SF-1.7.*** for details). According to [39], Monte Carlo-mediated analysis of flux statistics was carried out on the basis of a discrete approximation of optimized flux estimation distributions obtained in *L* multi-trials when the experimental data for each trial, comprising measured MIDs and effluxes, were artificially generated by corrupting of real initial data with normally distributed random errors. So, consecutive providing of *L* optimization trials finally resulted in *L*-set of optimized flux estimations:

3 $$ \mathbf{U}\left({\overset{\frown }{\theta}}_L\right)=\left(\mathbf{u}\left({\overset{\frown }{\boldsymbol{\uptheta}}}_1\right),\kern0.5em \mathbf{u}\left({\overset{\frown }{\boldsymbol{\uptheta}}}_2\right),\dots, \kern0.5em \mathbf{u}\left({\overset{\frown }{\boldsymbol{\uptheta}}}_{\mathrm{j}}\right),\dots, \kern0.5em \mathbf{u}\left({\overset{\frown }{\boldsymbol{\uptheta}}}_L\right)\right), $$

where ${\overset{\frown }{\theta}}_L=\left({\overset{\frown }{\boldsymbol{\uptheta}}}_1,{\overset{\frown }{\boldsymbol{\uptheta}}}_2,\dots, {\overset{\frown }{\boldsymbol{\uptheta}}}_{\mathrm{L}}\right)$ and $\mathbf{u}\left({\overset{\frown }{\boldsymbol{\uptheta}}}_j\right)={\left({u}_1\left({\overset{\frown }{\boldsymbol{\uptheta}}}_j\right),{u}_2\left({\overset{\frown }{\boldsymbol{\uptheta}}}_j\right),\dots, {u}_n\left({\overset{\frown }{\boldsymbol{\uptheta}}}_j\right)\right)}^T,j=1,2,\dots, L$. Finally, the upper and lower bounds of the $C{I}_{\gamma}^{MC}$ confidence interval at a determined confidence level of *γ* were evaluated for each flux on the basis of these distributions according to “discarding” or “mean-varianced” strategies (see Additional file 1: ***SF-1.7.,*** for details, and Figure 4 for clarity). In OpenFLUX2, the first group of the tunable parameters was implemented in the Monte Carlo-based procedure which modifications could increase/decrease the precision of global minimum search during optimization process in each trial. It was necessary to tune these control parameters up to the levels when their further modification does not significantly decrease the width of $C{I}_{\gamma}^{MC}\left({u}_i\right)$. Testing the several control parameters of the first group resulted in a set of their default values provided the convergence of the $C{I}_{\gamma}^{MC}\left({u}_i\right)$ bounds. Evaluations of these parameters, partially, were presented in the Additional file 2: Figure SF-2.4 as direct visualization of the computed interval bounds in dependence on provided trials at the different values of control parameters.

Usually, the Monte Carlo search of $C{I}_{\gamma}^{MC}\left({u}_i\right)$ bounds is based on an assumption that their estimated values have to converge in case of significant increasing of the total number of trials. So, the number of trials, *L*, is one of the most important parameters of Monte Carlo-based procedure, and it has to be optimally chosen (*L* = *L*_MAX_) for $C{I}_{\gamma}^{MC}\left({u}_i\right)$ precise determination in a reasonable computation time. The special procedure was implemented in OpenFLUX2 for a control of an essential number of optimization trials that was performed during target flux $C{I}_{\gamma}^{MC}\left({u}_i\right)$ bound estimation. This control finalizing in determination of all $C{I}_{\gamma}^{MC}\left({u}_i\right)$, could be realized ONLINE, with a help of specially preset control parameters (the second group of parameters implemented for fine tuning of the Monte Carlo-based search procedure), or according to direct user’s decision based on visualization of estimated bound plots in dependence on the current *L*, and flux estimation histograms that could be presented after the predetermined *L*_MAX_ trials were performed.

**Figure 5:**
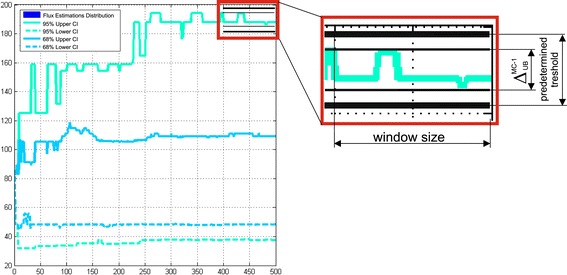
**Visualization of the** ***θ***_**22**_ **flux confidence interval bounds convergence.** Achieved as could be seen in the “window” due to an absolute spread of an upper confidence interval bound evaluated according to “discarding” Monte Carlo-based strategy is smaller than the predetermined threshold.

**Figure 6:**
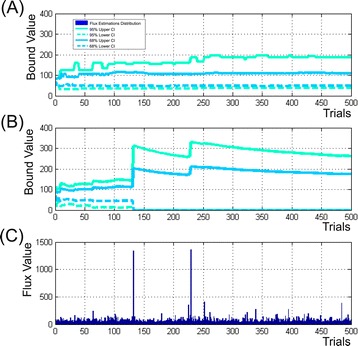
**Illustration of the** ***θ***_**22**_ **flux confidence bound convergence process in dependence on the number of performed trials computed according to “discarding” (A) and “mean-varianced” (B) strategies of Monte Carlo-based approach.** **(C)** – distribution of the ${\overset{\frown }{\theta}}_{22}$ flux estimations presented in accord to their appearance in *i-*th trial.

The developed Monte Carlo-based approach was used for determination of $C{I}_{\gamma}^{MC}\left({u}_i\right)$ for the solution of the constrained NLLSP described above (Table 1 (**Exp_3.6**)). The optimized flux parameter distributions were obtained for *L*_MAX_ = 500 trials using the default set of tunable control parameters that facilitate search of global minimum in each trial of the applied “multi runs per trial” fitting strategy (N_AS_ =3, K_NR_ =50, $\mathrm{T}\mathrm{T}=1\times {10}^{-4},\varepsilon =1\times {10}^{-4}$, see Additional file 1: ***SF-1.7.*,** for details). Then, for all 51 fluxes of assumed metabolic network the sliding control was performed for upper and lower bounds of $C{I}_{\gamma}^{MC}\left({u}_i\right)$ evaluated initially according to “discarding” strategy (i.e., bounds of $C{I}_{\gamma}^{MC-1}\left({u}_i\right)$, see definition in Additional file 1: ***SF-1.7.***) with the “window” size equaled to 40 trials. As could be seen from Additional file 3: Table SF-3.6, and Figure 5 (the later presented the convergence process of confidence bounds of the $C{I}_{0.95}^{MC-1}\left({\theta}_{22}\right)$ interval, as example), the ${\delta}_{\left(L/U\right)B}^{MC-1}$ (defined by Eq. (*S* − 1.7.7)) relative spreadings for the both upper and lower bounds of $C{I}_{0.95}^{MC-1}\left({u}_i\right)$ for 42 from 51 fluxes were dispersed between 0.1 and 1.0% in the last “window”, that could be considered as rather rigorous conditions of convergence. Moreover, if a value of ${\delta}_{\left(L/U\right)B}^{MC-1}\left(\gamma, i,{\overset{\frown }{\theta}}_L,M\right)$ had been equaled to 1.0% as the predetermined threshold, this level of convergence was achieved for more than 70% fluxes after already 20 – 50 optimization trials performed. The residual 30% of determined bounds converged after 400–420 trials according to these rigorous conditions. Eight residual fluxes (${\theta}_{10,}{\theta}_{12,}{v}_{13},{\theta}_{14},{\theta}_{22,}{v}_{23},{v}_{29}$ and *v*_36_) demonstrated the convergence at the relax conditions for upper and/or lower bounds, when ${\delta}_{\left(L/U\right)B}^{MC-1}\le 10\%$ at the level of confidence *γ* = 0.95, and this criterion was satisfied when 200 – 440 trials were performed. Five fluxes (${\theta}_{10},{\theta}_{14},{\theta}_{31},{v}_{29}$ and *v*_36_) had the specific feature: the values of lower bounds of their $C{I}_{0.95}^{MC-1}\left({u}_i\right)$ were closed to zero. So, these bounds, manifesting the obvious convergence, had to be restricted at the level of absolute (not relative) value of spreading (defined in Eq. (*S* − 1.7.6)), e.g., ${\Delta}_{LB}^{MC-1}\left(\gamma \right)\le 2$. In any case, all bounds of $C{I}_{\gamma}^{MC-1}\left({u}_i\right)$ could be obviously determined after the Monte Carlo-based optimized flux estimation had been obtained after *L*_MAX_ =500 performed trials in case of “discarding” strategy was used. In case of “mean-varianced” strategy was applied, the main part of $C{I}_{0.95}^{MC-2}\left({u}_i\right)$ (see definition in Additional file 1: ***SF-1.7.***) bounds (for 41 of 51 fluxes) converged earlier than *L*_MAX_ trials were performed under the rigorous convergence conditions, bounds for six additional fluxes demonstrated the convergence under relaxed conditions, and the corresponding bounds of $C{I}_{0.95}^{MC-2}\left({u}_i\right)$ were rather closed to the earlier determined bounds $C{I}_{0.95}^{MC-1}\left({u}_i\right)$. At the same time, the upper bounds for four residual fluxes (${v}_9,{\theta}_{10},{\theta}_{22},$ and *v*_23_) did not finalize the convergence process after 500 performed trials (Figure 6B): their minimal upper bound values of $C{I}_{0.95}^{MC-2}\left({u}_i\right)$ were gradually decreased in dependence on increased *L* at the late stage of computation, nevertheless, they remained significantly higher than the corresponding upper bounds of $C{I}_{0.95}^{MC-1}\left({u}_i\right)$ (Figure 6A). As could be seen from the (Figure 6C), the reason of this rather slow-speed convergence of several flux bounds was appearance of a small quantity of flux estimations significantly exceeded the ordinary values: “mean-varianced” strategy could not discard these outstanding values and had to accumulate a lot of usual estimations for significant decreasing the totally calculated “mean”.

Summing up, it could be concluded that obtaining a proper approximation of the optimized flux parameters was the most important part of the Monte Carlo based search of $C{I}_{\gamma}^{MC}\left({u}_i\right)$: even rather small quantity of significantly differed “outstanding” values in the flux estimations distribution that were obtained when the global minimum was not achieved in the fitting procedure, could significantly increase the width of the evaluated $C{I}_{\gamma}^{MC}\left({u}_i\right)$. In the tested cases, the convergence of $C{I}_{\gamma}^{MC}\left({u}_i\right)$ bounds was achieved faster (at the smaller number of the performed trials) if the “discarding”, but not “mean-varianced” strategy of the bound determination was used. So, the “discarding” strategy manifested significantly higher resistance for these occasional “outstanding” estimations. Moreover, this strategy was preferable to demonstrate the asymmetric character of optimized flux estimation distribution resulting in the asymmetric locations of the upper and lower bounds presented simultaneously for $C{I}_{0.95}^{MC-1}\left({u}_i\right)$, and $C{I}_{0.68}^{MC-1}\left({u}_i\right)$, in comparison with symmetric locations of their bounds obtained in case of “mean-varianced” strategy was used (Figure 4).

The later feature of the $C{I}_{\gamma}^{MC-1}\left({u}_i\right)$ bounds corresponded well with the parameters of $C{I}_{\gamma}^{n-lin}\left({u}_i\right)$ independently obtained by non-linear search developed by Antoniewicz et al. in [28] and implemented in OpenFLUX (see Additional file 1: ***SF-1.6.*** for details). All obtained data were presented in the Additional file 3: Table SF-3.6. As could be seen, the most part of $C{I}_{\gamma}^{n-lin}\left({u}_i\right)$ parameters coincided rather well with Monte Carlo-based results, especially with $C{I}_{\gamma}^{MC-1}\left({u}_i\right)$ bounds (this was true from 39 of 51 fluxes). Nevertheless, in several cases evaluations of the *CI*_*γ*_(*u*_*i*_) bounds given by Monte Carlo and non-linear approaches differed, and some times significantly (e.g., $U{B}_{0.95}^{MC-1}\le U{B}_{0.95}^{n-lin}$ and, on the contrary, $U{B}_{0.68}^{MC-1}\ge U{B}_{0.68}^{n-lin}$ for the *θ*_14_, and *θ*_22_ fluxes; both determined upper bound values for the *θ*_31_ -flux confidence interval, $U{B}_{0.95}^{n-lin}$ and $U{B}_{0.68}^{n-lin}$, was lower in case on non-linear computing). Absolutely statistically incorrect result was obtained for 9 fluxes (e.g., *θ*_32_, *θ*_33_, *v*_2_, *v*_5_ and *etc*.): estimated upper bound of $C{I}_{0.68}^{n-lin}\left({u}_i\right)$ had higher values than their upper bound of $C{I}_{0.95}^{n-lin}\left({u}_i\right)$. It seems that these “mistakes” appeared as a result of low accuracy of numerous calculations performed according to non-linear search-based algorithm in the used software. It finally resulted in termination of computation even when necessary optimality conditions were not satisfied and the real global minimum was not reached in the optimization procedure. The proposed modifications targeted to improvement of the calculation efficiency are at the final stages of testing and implementation in new release of OpenFLUX2 software (see, Additional file 1: ***SF-1.6.,*** for details). Up today, the current version of OpenFLUX2 contains the initial variant of non-linear algorithm of flux confidence intervals search. Keeping in mind incorrect results that could be computed now for $C{I}_{\gamma}^{n-lin}$ of some fluxes, that are very difficult to recognize without information for comparison, but, on the other hand, very high speed of all flux $C{I}_{\gamma}^{n-lin}\left({u}_i\right)$ computation (about one hour for estimation flux statistics for SLE using computers described in Methods), it could be highly recommended to use the current algorithm of $C{I}_{\gamma}^{n-lin}\left({u}_i\right)$ non-linear search mainly for quick preliminary evaluation. The accurate determination of *CI*_*γ*_(*u*_*i*_) could be performed, e.g. as $C{I}_{\gamma}^{MC-1}\left({u}_i\right)$, according to the fine-tunable Monte Carlo based approach with automatic and/or visual control of all bounds convergence.

#### Normalized flux precision function as a measure of flux resolution efficiency

To compare the achieved efficiency of the *u*_*i*_ flux resolution, it is convenient to use the normalized flux precision function (see, Additional file 1: ***SF-1.6.***) with *β* as the scaling parameter:

4 $$ {\eta}_{\gamma}^{MC-1}\left({u}_i\left(\overset{\frown }{\boldsymbol{\uptheta}}\right),\beta \right)=\left\{\begin{array}{l}1-\frac{C{I}_{\gamma}^{MC-1}\left({u}_i\right)}{u_i\left(\overset{\frown }{\boldsymbol{\uptheta}}\right)+\beta \cdot \max {\mathbf{V}}_{eff}^{mea}}\cdot,\ \mathrm{when}\ C{I}_{\gamma}^{MC-1}\left({u}_i\right)\le {u}_i\left(\overset{\frown }{\boldsymbol{\uptheta}}\right)+\beta \cdot \max {\mathbf{V}}_{eff}^{mea}\\ {}0,\ \mathrm{when}\ C{I}_{\gamma}^{MC-1}\left({u}_i\right)>{u}_i\left(\overset{\frown }{\boldsymbol{\uptheta}}\right)+\beta \cdot \max {\mathbf{V}}_{eff}^{mea}\end{array}\right. $$

Generally, in this function, ${u}_i\left(\overset{\frown }{\boldsymbol{\uptheta}}\right)$ is the best available estimation of the unknown true value that can be computed from the SLE- or PLE-based ^13^C-MFA. In the case of computer simulations, the “true” flux parameters are known: namely ${u}_i\left(\overset{\frown }{\boldsymbol{\uptheta}}\right)={\left({u}_i\right)}_{true}$ were used for the calculation of *η*_*γ*_(*u*_*i*_; *β*) values in the present study. In turn, the superscript employed for the *η*_*γ*_(*u*_*i*_; *β*) and *CI*_*γ*_(*u*_*i*_) functions, e.g., *n-lin* and *MC* − 1, respectively, indicates the method of estimation of the flux confidence interval. So long as the Monte Carlo-based approach with application of “discarding” strategy was used in all examples of the present study as the main method for flux confidence interval determination, we would not specially indicate below the way of determination of flux confidence intervals using the superscript “^*MC*-1^”. The variable scaling parameter *β* was set to 0.1 in the present study, and the dependence of the *η*_*γ*_(*u*_*i*_) function on *β* as the parameter is not directly indicated in the corresponding equations below for brevity. According to its definition, the *η*_*γ*_(*u*_*i*_) function at each fixed *β* parameter is close to “1” for precisely estimated *u*_*i*_ fluxes (with narrow confidence intervals) and is close to “0” for a poorly determined *u*_*i*_.

**Figure 7:**
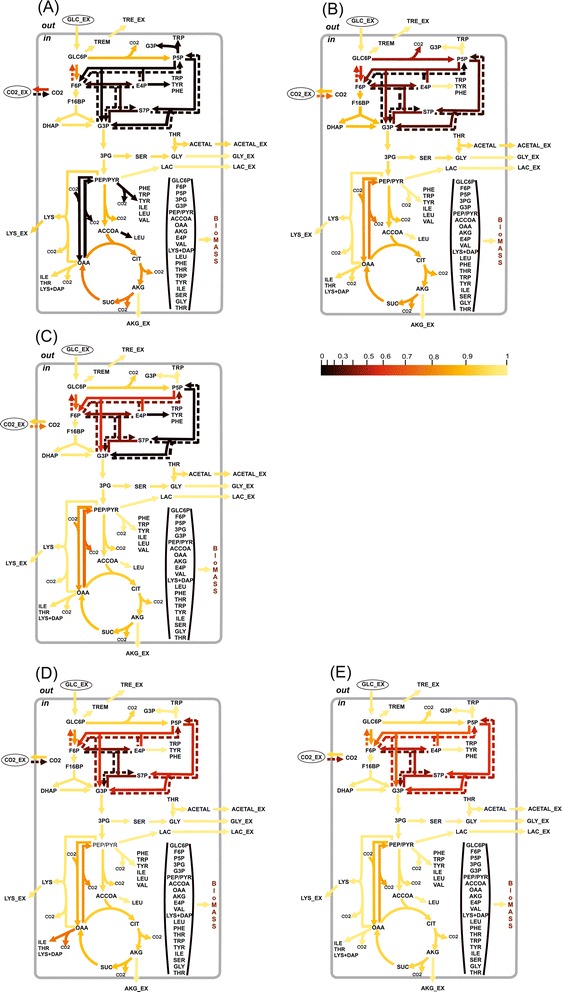
**The values of the normalized flux precision function in the different parts of the assumed metabolic network depend significantly on the applied tracer(s).** The results of flux estimation: **(A)** SLE using 100% [1-^13^C]-glucose as the tracer; and from PLEs consisting of: **(B)** 5 LEs using different mixtures of [U-^13^C]/[U-^12^C]-glucose in each LE, with 20%, 35%, 50%, 65%, or 80% [U-^13^C]-glucose; **(C)** 5 LEs using partially optimized mixtures of [1-^13^C]/[U-^12^C]/[U-^13^C] glucose for the separate minimization of the approximated variances of the *θ*_10_, *θ*_12_, *θ*_14_, *θ*_22_, *θ*_31_ free fluxes in each LE; **(D)** 3 LEs using 100% [1-^13^C]-, [3-^13^C]-, or [4-^13^C]-glucose as a tracer; **(E)** 6 LEs, where 3 of the LEs used in **(D)** corresponded to partial optimization for *θ*_4_and*θ*_12_, *θ*_14_, *θ*_22_and*θ*_31_, respectively, and 3 other LEs used the partially optimized mixture of [1-^13^C]/[3-^13^C]/[4-^13^C]-glucose to minimize the approximated variances of the *θ*_8_, *θ*_10_, *θ*_26_ free fluxes. The values of the normalized flux precision function for all fluxes are indicated by the color-scored grade.

**Figure 8:**
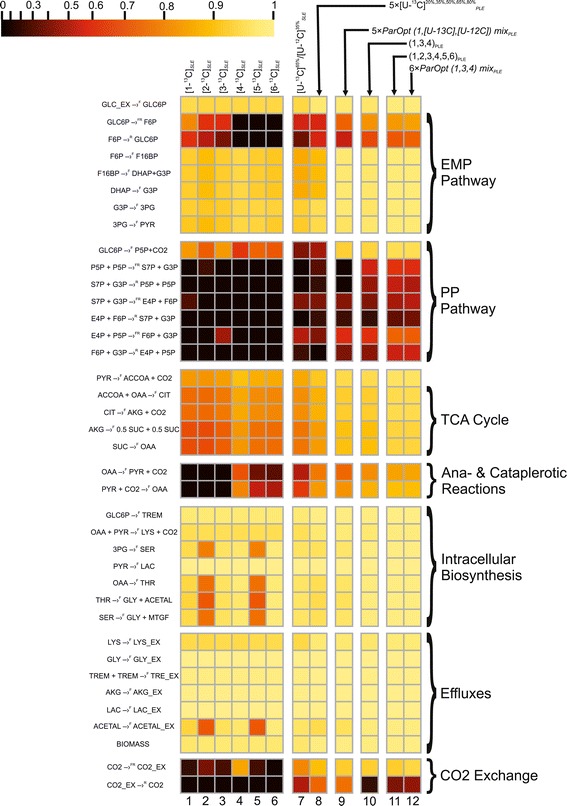
**Normalized flux precision functions for all fluxes are presented in the form of color-scored squared diagrams for the different tracers used for simulations.** The columns correspond to the following labeling experiments: **1**, **2**, …., **6** − SLEs with 100% [*i*-^13^C]-glucose as a tracer, where *i* = 1, 2, …, 6, respectively; **7** − an SLE with a generally optimized (minimum *D*-value) mixture of [U-^13^C]^65%^/[U-^12^C]^35%^-glucose; **8** − a PLE consisting of 5 LEs with different mixtures of [U-^13^C]/[U-^12^C]-glucose in each LE, where the fraction of [U-^13^C]-glucose was 20%, 35%, 50%, 65%, or 80%; **9** − a PLE consisting of 5 LEs with partially optimized mixtures of [1-^13^C]/[U-^13^C]/[U-^12^C] glucose for separate minimization of the approximated variances of the *θ*_10_, *θ*_12_, *θ*_14_, *θ*_22_, *θ*_31_ free fluxes in each LE; **10** − a PLE composed of 3 LEs using 100% [1-^13^C]-, [3-^13^C]-, or [4-^13^C]-glucose as the tracer; **11** − a PLE consisting of 6 LEs, where all singly labeled isotopomers of glucose, as in **1 – 6**, were used as tracers; **12** − a PLE composed of 6 LEs, including 3 of the LEs used in **10**, corresponding to partial optimization for *θ*_4_ and *θ*_12_, *θ*_14_, *θ*_22_and*θ*_31_, and 3 other LEs using the partially optimized mixture of [1-^13^C]/[3-^13^C]/[4-^13^C]-glucose to minimize the approximated variances of the *θ*_8_, *θ*_10_, *θ*_26_ free fluxes.

The computed values of *η*_*γ*_(*u*_*i*_) for the obtained solution of the NLLSP were flux specific and varied from zero to almost unity, with a sum of ${\varSigma}_{\eta (0.95)}={\displaystyle \sum_{\mathrm{i}=1}^n{\eta}_{0.95}\left({u}_i\right)}=38.3$ (see Additional file 3: Table SF-3.6 for details). The flux specificity of the *η*_*γ*_(*u*_*i*_) function for the determined metabolic model was subsequently shown to be primarily dependent on the applied labeled tracer (see Figures 7 and 8). Indeed, the high resolution of one set of fluxes (e.g., *v*_3_: GLC6P →^FR^ F6P; *θ*_4_: F6P →^R^ GLC6P; *v*_25_: 3PG→^F^ SER; *θ*_26_: SER→^F^ GLY + MTHF, with values of ${\eta}_{0.95}\left({u}_i\right)\in \left[0.65,0.96\right]$) and the low resolution of another set of fluxes (e.g., *θ*_22_: PYR + CO2→^F^ OAA; *v*_23_: OAA →^F^ PYR + CO2; *θ*_31_: CO2_EX →^R^ CO2, with *η*_0.95_(*u*_*i*_) = 0) are rather typical for experiments using [1-^13^C]-glucose as the labeled tracer (Figure 7 (A), Figure 8: Column 1). The following indication of the *η* -function emphasizes the type of tracer used and the SLE- or PLE-based character of the provided ^13^C-MFA: ${\eta}_{0.95}\left({u}_i;{\left[1{-}^{13}\mathrm{C}\right]}_{SLE}\right)$.

Concerning the tracer-specific values of *η*_*γ*_(*u*_*i*_), it was interesting to estimate the sensitivity of this function, which is dependent on flux variances, in the case of five simulated SLEs with the same tracer. The corresponding calculations were provided for 5 independent SLE with earlier simulated MIDs and the measured effluxes with the $N\left(\mathbf{0},{\boldsymbol{\upsigma}}^{mea}\right)$ normally distributed random errors (Additional file 3: Table SF-3.6 (Exp.3.1-Exp.3.5)). These data are summarized in Additional file 2: Figure SF-2.5. The calculated mean-doubled measured specific variances did not exceed 0.02 for nearly all fluxes, and they therefore practically did not change the tracer-specific profile of the *η* -function. The summarized values of the *η* -function for all fluxes, (*Σ*_*η*(0.95)_([1 − ^13^C]_*SLE*_))_*k*_, which were calculated for each flux from *k* = 1, 2, …, 5 SLEs, varied in a rather narrow range (between 37.9 and 38.5). Considering the total number of fluxes (equal to 51) for the assumed metabolic model and the detected measurement-dependent sensitivity of the *η* -function, if the difference between the *Σ*_*η*(0.95)_ values is no greater than 1, then the LEs can be considered to be provided with an essentially equivalent flux precision. Certainly, the value of *Σ*_*η*(0.95)_ could be considered only as a general, conditional measure of flux resolutions in the investigated network: some metabolic branches could be resolved better, and other branches – worse in different experiments with, perhaps, equal values of summing normalized flux precision functions. So, only the value of the *η*_*γ*_(*u*_*i*_) -function could be considered as the absolute measure of the *u*_*i*_-flux resolution efficiency that could be compared with other LEs performed with the same metabolic model.

#### The necessity of a comprehensive statistical analysis of the NLLSP solution

In the present study, the results of the ^13^C-MFA included the set of estimated fluxes, the goodness-of-fit of the model’s adequacy, and the confidence intervals of fluxes, in accord with the recently recommended publishing guidelines [70]. As shown in Additional file 1: ***SF-1.4.***, the comprehensive goodness-of-fit analysis had to consist of the *χ*^2^ -mediated testing of the Ξ(**θ**) objective function at the point of convergence, $\overset{\frown }{\boldsymbol{\uptheta}}$, and confirmation that the individual weighted residuals used as the summands of this function were $\mathrm{N}\left(0,1\right)$ distributed. The solution of the constrained NLLSP was considered statistically acceptable only if all of the tests were successfully passed.

In this report, an example is presented in which the mistaken flux parameters could be assumed when the statistical analysis of the obtained solution was partially provided but to an insufficient extent. Let us analyze the first from five earlier described *in silico* experiments with 99% [1-^13^C]-glucose as a tracer (see Table 1**(Exp_3.1)**). The “contribution matrix” (see Additional file 1: ***SF-1.5***), $\mathbf{C}\mathbf{M}, \dim \left(\mathbf{C}\mathbf{M}\right)=\left(n\times w\right)$, was computed at the true point, **u**(**θ**_*true*_), as an important component of the flux statistics (Additional file 3: Table SF-3.12). It is known [28] that the (**CM**)_*ij*_ elements of this matrix indicate the relative importance of the variance of the *j*-th measurement to the local variance of the *i*-th flux. As can be observed in Additional file 3: Table SF-3.12, the variances of the “serine” MIDs demonstrated the high importance of the flux resolution among MS measurements; all matrix columns corresponding to the SER mass isotopomers showed rather high sums of their elements. The variances of these “serine” (“SER”) MIDs significantly influenced the resolution of several fluxes (${\theta}_8,{\theta}_{22},{v}_{23},{\theta}_{31}$) for the following reactions: GLC6P →^F^ P5P + CO2; PYR + CO2 →^F^ OAA; OAA →^F^ PYR + CO2; and CO2_EX →^R^ CO2, respectively. The new set of “experimental data” was generated in the following fashion: the measured effluxes and all MIDs, except for “SER” MIDs, were considered as in the previously analyzed example. The “SER” MIDs were modified as in the case of “poor” resolution of the SER-390 (m + 0) MID, which exhibited an unknown by-product that increased the value of the corresponding SER peak to +4.5%. Due to the necessity of normalizing all SER-isotopomers, the applied modification resulted in a proportional decrease in the other SER MID portions; therefore, the SER-390 MIDs were modified from (m + 0)/(m + 1)/(m + 2)/(m + 3) = 0.443/0.357/0.140/0.042 (in the previously described example) to a ratio of 0.463/0.344/0.135/0.040.

**Figure 9:**
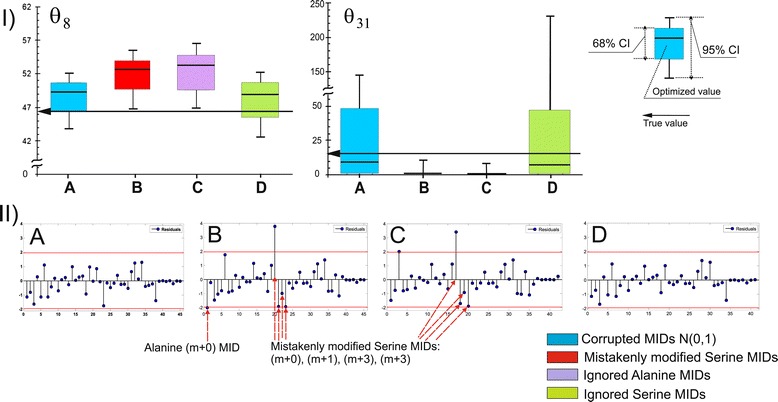
**(I) – Determination of optimized parameters and confidence intervals for the** ***θ***_**8**_, ***θ***_**31**_ **fluxes** **from** **(A)** **“properly” corrupted and** **(B)** **“mistakenly” corrupted values of SER MIDs, and after the “elimination” of residuals corresponding to ALA MIDs** **(C)** **or SER MIDs** **(D)** **from the expression for the objective function.** **(II) – Stem plots of the variance-weighted individual residuals at the point of convergence.** The individual residual values are represented by the leaves of the stem plots sorted in accordance with their enumeration provided by the model. Additional red horizontal lines represent the 0.95-quantile values of the standard normal distribution Ν(0, 1). The efflux values, estimated from an SLE, are usually rather close to the measured values; the last seven points, indicating the residuals corresponding to the efflux measurements, are close to zero. In the case of the PLE in which efflux measurements were generated independently for each SLE making up the PLE, this tendency was not observed.

This “mistakenly” modified set of “experimental data” was used to generate the newly constrained NLLSP. A unique solution, corresponding to the minimal values of the Ξ = Ξ(**θ**) objective function with the empty null space of the **J**_**f**_(**θ**) matrix evaluated at the new point of convergence, $\boldsymbol{\uptheta} ={\overset{\frown }{\boldsymbol{\uptheta}}}_{\mathrm{new}}$, was found. Moreover, the value of $\Xi \left({\overset{\frown }{\boldsymbol{\uptheta}}}_{\mathrm{new}}\right)$ was in the *χ*^2^ -statistically acceptable interval (see Table 1**(Exp_4.1)**). Simultaneously, the parameters obtained for several fluxes did not coincide with the true flux values even in the range of their confidence intervals. Among them there were fluxes *θ*_8_ and *θ*_31_ (Figure 9 (I - B)) which exhibited variances that were essentially determined by SER MID measurements. The fact that the obtained flux parameters were incorrect could be established only at the stage of normality distribution testing of the weighted residuals; three variance-weighted residuals corresponding to “SER-390” MIDs and one corresponding to “alanine (ALA)-260” were shown to be located outside of the 95% confidence interval (Figure 9 (II-B)). According to the general recommendation presented in ***SF-1.4***, improvement of the solution statistics could be achieved due to the excision of the “prominent” residuals from the expression for the objective function followed by the repeated solution of the constrained NLLSP with the partially truncated Ξ function. Two alternative NLLSPs were generated where ALA or SER MIDs were excluded from the “experimental data” set. Parameters obtained after solution of the NLLSP with ignored “ALA-260” MIDs did not coincide with the true flux values in the range of their confidence intervals (Figure 9 (I-C)). Moreover several variance-weighted residuals still located outside of the 95% confidence interval including that of “SER-390” (Figure 7 (II-C)). In contrast, the proper statistically acceptable solution was obtained for NLLSP with ignored “SER-390” MIDs, which coincided with true fluxes in the range of the determined flux confidence intervals (Figure 9 (I-D) and (II-D)). Thus, “incorrect flux parameters” could be assumed if test for normality of variance-weighted individual residual distribution is ignored. Certainly, higher negative influence on the proper flux estimation could be assumed if *χ*^2^ -statistically unacceptable solution is obtained. Analysis of variance-weighted residuals plot followed by step by step exclusion substances with prominent residuals, as described in the example, could help to identify “incorrect measurements” and, perhaps, to obtain statistically acceptable solution with correctly estimated parameters.

It is obvious that measured MIDs and effluxes’ parameters are completely separate categories of experimental data that could not be trivially compared. It is well established [3] that labeling experiments are performed to resolve internal fluxes, even parallel and cycle pathways, and reversible reactions, which cannot be resolved on the basis of measured effluxes. Nevertheless, the variances of the measured effluxes could provide the most significant influence on the resolution of some fluxes, as could be seen from the values of the corresponding (**CM**)_*ij*_ elements in the Additional file 3: Table SF-3.12. Thus, generally, it is desirable to execute the efflux measurements with the highest possible accuracy to decrease the variances and totally improve the flux resolution. One of the interesting step in this direction has been done recently when the authors tried to increase the measurement accuracy for an efflux corresponding to quantifying biomass composition due to exploiting of the high-precision GC-MS technique [71]. Unfortunately, in many cases, the efflux measurements as the stage of labeling experiment have received much less attention than the more glamorous stages of the subsequent highly-precised MS-based measurements.

#### Simulations of LEs with [U-^13^C]-glucose as a tracer

A uniformly ^13^C-labeled isotopomer of glucose, [U-^13^C]-glucose, is often used as a tracer in ^13^C-MFA. In the present study, new sets of “experimental data” were generated for the same metabolic model when different relative amounts of [U-^13^C]-glucose (20%, 35%, 50%, 65%, and 80%) mixed with non-labeled ([U-^12^C]) glucose were used as the sole carbon source (Additional file 3: Table SF-3.8).

Statistically acceptable solutions were obtained for constrained NLLSPs for five independent SLEs and for a PLE using all of the generated “experimental data” for fitting to the single metabolic model (see Table 1**(Exp_5.1-Exp_5.5; PLE_1)** and Additional file 3: Table SF-3.8). As shown by the presented data, the SLE-based ^13^C-MFA resulted in values between 35.4 and 40.4 for the *Σ*_*η*(0.95)_([U − ^13/12^C]_*SLE*_) function (Additional file 3: Table SF-3.8). Again, a set of tracer-specific fluxes that possessed rather high values of the *η* -function could be detected (at least more than in the case of exploiting [1-^13^C]-glucose as a tracer), e.g., *θ*_22_: PYR + CO2 →^F^ OAA; *v*_23_: OAA →^F^ PYR + CO2; *θ*_31_: CO2_EX →^R^ CO2. The PLE-based ^13^C-MFA of experiments using [U-^13/12^C]-glucose as the carbon source actually improved the resolution of all fluxes estimated in the corresponding SLEs, *Σ*_*η*(0.95)_([U − ^13/12^C]_*PLE*_) = 42.4. Moreover, the tracer-specific behavior described in relation to SLEs was reproduced in the PLE. The colored scheme presented in Figure 7 and the diagram in Figure 8 correspond to the calculated values of the *η* -function for all fluxes obtained in different experiments, illustrating this fact and demonstrating that optimization of the experimental design is necessary to increase the precision of the targeted fluxes [19,49,54,62].

#### Optimal design for LEs using mixtures of [U-^13^C]-, [1-^13^C]-, and [U-^12^C]-glucose isotopomers

To achieve the best flux resolution in an LE employing a mixture [U-^13^C]-, [1-^13^C]-, and [U-^12^C]-glucose isotopomers as the carbon source, optimization of the experimental design could be performed according to the method proposed by Möllney et al. [19]. In their study, the comparison of different designs was based on an evaluation of *D*-factor values [72], i.e., the squared volumes of the flux confidence ellipsoids for a given confidence level. According to [19] and using the notations introduced in the present study, the squared volume of the *p*-dimensional confidence ellipsoid for free fluxes, which was evaluated at the point of convergence, $\boldsymbol{\uptheta} =\overset{\frown }{\boldsymbol{\uptheta}}$, could be obtained (up to a constant that does not depend on designed parameters) using the following expression:

5 $$ \mathrm{D}\left({\mathbf{m}}^{input},{\mathbf{m}}^{mea},{\boldsymbol{\upsigma}}^{mea},\overset{\frown }{\boldsymbol{\uptheta}}\right)= \det {\Sigma}_{\overset{\frown }{\boldsymbol{\uptheta}}}\left({\mathbf{m}}^{input},{\mathbf{m}}^{mea},{\boldsymbol{\upsigma}}^{mea},\overset{\frown }{\boldsymbol{\uptheta}}\right)= \det {\left[{\mathbf{H}}_{SSR}\left(\overset{\frown }{\boldsymbol{\uptheta}}\right)\right]}^{-1} $$

where ${\Sigma}_{\overset{\frown }{\boldsymbol{\uptheta}}}$ is a covariance matrix of the free fluxes estimated according to linearized statistics (see Additional file 1: ***SF-1.5.***) and to the approximation of this matrix by the inverse Hessian matrix presented in Eq. (*A*2 − 10) Additional file 1: ***SF-1 Appendix 2***. Specifically, the last equality from Eq. (5) was used for computation of the *D*-factor. According to a previous suggestion [19], the use of the value of the $\sqrt[2p]{D}$ parameter, which can be interpreted as an average length of the confidence interval of estimated fluxes, is more suitable. As noted previously, the confidence ellipsoid volume estimation provided by Eq. (5) holds true within some vicinity of the predefined point $\boldsymbol{\uptheta} =\overset{\frown }{\boldsymbol{\uptheta}}$ and can change due to a shift in that point [19]. Because the true values of the free fluxes were known for our artificial metabolic model, the optimization of the experimental design, which was dependent on the tested ^13^C-labeled tracers, was significantly simplified and was based on the computation of the determinant of the inverse Hessian matrix evaluated at the point **θ** = **θ**_*true*_. In practice, when the true flux values are not known *a priori*, they are assumed, for example, from data in the literature, FBA or even from ^13^C-MFA performed on the basis of a rather inexpensive label [37,63]. In some cases, a second round of experimental design optimization is necessary if the proposed flux distribution is far from the feasible point of convergence obtained in a planned labeling experiment [73,74]. In the present study, all of the new experimental designs were compared with the reference design (SLE with 99% [1-^13^C]-glucose as the tracer) using the value of the $\sqrt[2p]{D}$ parameter. In addition to the “general optimization” achieved due to the minimization of the relative value of the average confidence interval length:

6 $$ {\overline{\mathrm{D}}}_{opt}\left({\mathbf{m}}^{input},{\mathbf{m}}^{mea},{\boldsymbol{\upsigma}}^{mea},{\boldsymbol{\uptheta}}_{true}\right)=\underset{{\mathbf{m}}^{input}- feasible}{ \min}\sqrt[2p]{\frac{{\mathrm{D}}_{new}\left({\mathbf{m}}^{input},{\mathbf{m}}^{mea},{\boldsymbol{\upsigma}}^{mea},{\boldsymbol{\uptheta}}_{true}\right)}{{\mathrm{D}}_{ref}\left({\mathbf{m}}^{ref},{\mathbf{m}}^{mea},{\boldsymbol{\upsigma}}^{mea},{\boldsymbol{\uptheta}}_{true}\right)}} $$

“partial optimization” could be obtained as a result of minimization of the standard deviation of the targeted *θ*_*i*_ free flux, which, in turn, was approximated as the square root of the corresponding diagonal element of the ${\Sigma}_{\overset{\frown }{\boldsymbol{\uptheta}}}$ matrix:

7 $$ \underset{{\mathbf{m}}^{input}- feasible}{ \min }{\left({\boldsymbol{\upsigma}}_{lin}^{{\boldsymbol{\uptheta}}_{true}}\right)}_i=\underset{{\mathbf{m}}^{input}- feasible}{ \min}\sqrt{{\left({\left[{\mathbf{H}}_{SSR}\left({\boldsymbol{\uptheta}}_{true}\right)\right]}^{-1}\right)}_{ii}} $$

The results of the corresponding computations performed using the special subprograms implemented in OpenFLUX2 software are presented in Additional file 2: Figure SF-2.6. According to the provided calculations, “general optimization” required the use of a mixture of [1 − ^13^C]^78 %^/[U − ^13^C]^22 %^ glucose isotopomers in the SLE (Additional file 2: Figure SF-2.6 (A)). Interestingly, these *D*-factor-mediated optimized tracer compositions were extremely close to the [1 − ^13^C]^80 %^/[U − ^13^C]^20 %^ -labeled mixture of glucose that has been used without any calculations to achieve a rather high flux resolution in other experimental systems (e.g., *Escherichia coli*-based systems [48], in particular). In contrast, the same *D-*criterion-based approach resulted in another optimal mixture of the same glucose isotopomers ([1 − ^13^C]^48 %^/[U − ^13^C]^40 %^/[U − ^12^C]^12 %^) when some modifications differed in the metabolic model of the l-lysine-producing *C. glutamicum* strain applied in the present study, and plans were made to obtain another set of measurements [19].

**Figure 10:**
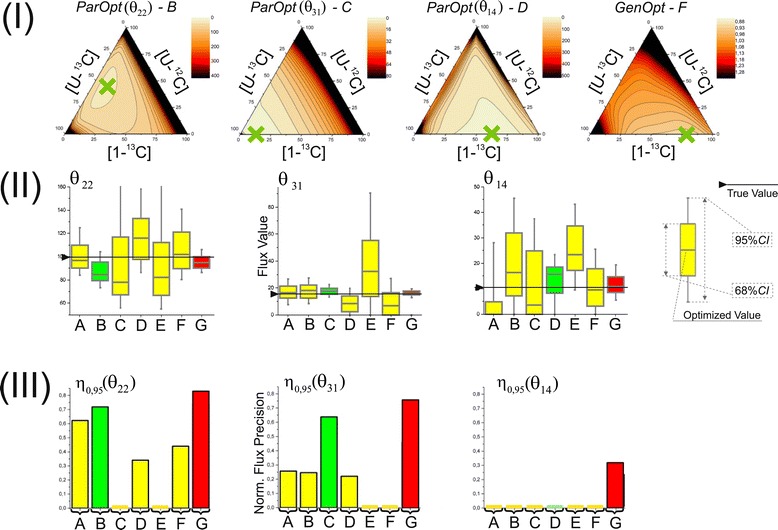
**Experimental design studies** **for the identification of mixtures of [1-** ^**13**^ **C]/[U-** ^**12**^ **C]/[U-** ^**13**^ **C]-glucose isotopomers for “general” optimization or for the best resolution (“partial” optimization) of** ***θ***_**31**_, ***θ***_**14**_, ***θ***_**22**_ **fluxes** **(I)** **, results of these** **flux estimations (II)** **obtained through** ^**13**^ **C-MFA of the simulated SLEs (** **A – F** **) or PLEs (** **G** **) with the mentioned mixed tracers:** **A** **− SLE, 0/50/50, %% (partially optimized for** ***θ***_**12**_ **);** **B** **− SLE, 10/44/46, %% (partially optimized for** ***θ***_**22**_ **);** **C** **− SLE, 13/0/87, %% (partially optimized for** ***θ***_**31**_ **);** **D** **− SLE, 66/0/34, %% (partially optimized for** ***θ***_**14**_ **);** **E** **− SLE, 89/0/11, %% (partially optimized for** ***θ***_**10**_ **);** **F** **−78/0/22, %% (generally optimized);** **G** **− PLE consisting of five LEs using the same tracers as in** **A – E** **for each experiment (red 68%** ***CI*** **boxes);** **(III) the normalized flux precision function values** **for the confidence level of** ***γ*** **= 0.95 computed for these** **(A – G)** **experiments.** Green 68% *CI* boxes in **(II)** indicate flux estimation resulted from ^13^C-MFA of SLE optimized for best resolution of this flux. The results of the experimental design studies computed by OpenFLUX2 were visualized in the form of a ternary plot generated by commercially available OriginPro 9.1 (Originlab Corp., Northampton, MA, USA) software.

According to the provided computations, “partial optimization,” i.e., the optimal resolution of different targeted free fluxes, had to be achieved in the SLEs with the distinguished mixtures of the same glucose isotopomers (e.g., see Additional file 2: Figure SF-2.6 (B - F)). Several of the proposed optimal designs (e.g., see Figure 10 (I)) were realized using computer simulations, and the set of experimental data was generated, followed by a search of the solution for the corresponding constrained NLLSP. One of the simulated SLEs was performed using the “generally optimized” mixed tracers, five others employed the “partially optimized” mixtures, and five experiments were performed with the randomly chosen mixture of [1 − ^13^C], [U − ^13^C] and [U − ^12^C] glucose isotopomers (“randomly mixed”) as the tracers. As shown in the presented results (Additional file 3: Table SF-3.9), the SLE with “generally optimized” conditions resulted in *Σ*_*η*(0.95)_([1 − ^13^C]^78 %^[U − ^13^C]^22 %^_*SLE*_) = 38.4, with “partially optimized” conditions – ${\varSigma}_{\eta (0.95)}\left( ParOp{t}_{SLE}\right)\in \left[36.0,38.7\right]$, and with “randomly mixed” tracers – ${\varSigma}_{\eta (0.95)}\left( RanMi{x}_{SLE}\right)\in \left[35.2,39.1\right]$. It has to be especially noted, that SLEs with the higher calculated values than *Σ*_*η*(0.95)_ = 38.4 was detected here, and among described earlier (see, final Additional file 3: Table SF-3.11), and so “general” and/or “partial” optimizations does not result in maximization of the sum of normalized precision functions for all fluxes, *Σ*_*η*(0.95)_. Simultaneously, the free fluxes, *θ*_*i*_, that were used as the targets for the partial optimization generally showed the best resolutions (i.e., the narrowest confidence intervals, *CI*_0.95_(*θ*_*i*_), (Figure 10 (II)) and the highest values of the *η*_0.95_(*θ*_*i*_) function (Figure 10 (III)) among all of the estimations obtained for different SLEs that could be provided using all possible combinations of the tested tracers. The detected exceptions (see Figure 10(II) (D)) could most likely be explained by the simplified linearized statistics used for design optimization; however, a Monte Carlo-based approach was later performed to calculate the non-symmetrical flux confidence intervals.

It was interesting to see the possible effect of “general” and “partial” optimizations when the corresponding set of independently analyzed SLEs was considered as LEs in a PLE, followed by rigorous fitting of all of the simulated “experimental” data to the single metabolic model. The set of PLE-based experiments consisted of 5 earlier analyzed SLEs consisted of “generally” and “partially” optimized, as well as “randomly mixed” tracers were analyzed. As could be expected, the PLE consisted of “generally” and/or “partially” optimized LEs were among experiments with the most precisely resolved fluxes (with the maximal values of *Σ*_*η*(0.95)_). On the other hand, the real difference between summarized values of the *η* -function for all fluxes determined for these PLEs was very small (between 42.7 and 44.2 see, Additional file 3: Table SF-3.9) and all of these values significantly exceeded the corresponding sum estimated for any SLEs among used in this comparison. So, the positive effect of PLE-based experiment provided with many LEs substantively differed in the used tracers was so significant, that further improvement of flux resolution due to additional optimization of experimental design could have rather marginal positive sense.

#### ^13^C-MFA for SLEs/PLEs with singly ^13^C-labeled-glucose tracers

It has been repeatedly shown [19,59,72] that significantly improved resolutions of fluxes from different parts of a metabolic network can be achieved using commercially available or specially synthesized singly or multiply ^13^C-labeled glucose isotopomers other than the previously applied and cheapest [1-^13^C] and [U-^13^C] variants. Moreover, the COMPLETE-MFA approach, using all singly labeled glucose tracers ([*i*-^13^C]-glucose, where *i* = 1, 2, …, 6) in the individual LEs of the PLE, was recently developed to evaluate metabolic fluxes for the metabolic model of wild-type *Escherichia coli* with a high precision [58]. Additionally, the PLE included two LEs with only [2-^13^C]-glucose, and separate [3-^13^C]-glucose in the medium provided the most precise fluxes for the *E. coli* model among all possible paired combinations of singly labeled glucose added as the sole tracer.

It was of interest to evaluate the advantages of this approach in the case of another metabolic model used in the present study. Initially, the possible exploitation of all six singly labeled ^13^C-glucose isotopomers, which were separately added to the medium as tracers for SLEs and PLEs, was investigated. The solutions of the corresponding constrained NLLSPs are summarized in Additional file 3: Table SF-3.10 and Figure 8: Columns 1 – 6. The following conclusions were drawn from these data. Initially, the values of the normalized flux precision function varied significantly for individual fluxes in a tracer-dependent manner, and it was not the best singly labeled glucose tracer for the estimation of all fluxes in the assumed metabolic model. Indeed, according to the formally computed value of the *D*-factor (see (*S* − 1.5.14)) for the six singly labeled tracers, the volume of the confidence ellipsoid for free fluxes had to be increased in the following order for the applied tracers:

8 $$ {D}_{\left[3{-}^{13}\mathrm{C}\right]}<{D}_{\left[1{-}^{13}\mathrm{C}\right]}<{D}_{\left[2{-}^{13}\mathrm{C}\right]}<{D}_{\left[4{-}^{13}\mathrm{C}\right]}<{D}_{\left[6{-}^{13}\mathrm{C}\right]}<{D}_{\left[5{-}^{13}\mathrm{C}\right]} $$

i.e., the minimal volume of the confidence ellipsoid for free fluxes could be obtained using [3-^13^C]-glucose as a singly labeled glucose tracer. Simultaneously, a different order of the tracers could be formed by increasing the summarized values of the normalized flux precision functions, *Σ*_*η*(0.95)_([*i* − ^13^C]_*SLE*_) ≡ *Σ*_*η*_(*i*), *i* = 1, 2, …, 6:

9 $$ 39.4={\varSigma}_{\eta }(4)>{\varSigma}_{\eta }(3)>{\varSigma}_{\eta }(1)>{\varSigma}_{\eta }(6)>{\varSigma}_{\eta }(2)>{\varSigma}_{\eta }(5)=36.6 $$

i.e., [4-^13^C]-glucose was the best tracer according to this *Σ*_*η*_ -based criterion; namely, [4-^13^C]-glucose was the best tracer for determining 13 fluxes, and the corresponding values of the *η* -function for this tracer for 28 other fluxes were among the maximal values in the range of no more than 0.02, which is a typical value for measurement-specific errors. Notably, the applicability of [4-^13^C]-glucose as the tracer for the efficient resolution of primary branch points and reversibilities was previously demonstrated for a similar *C. glutamicum* metabolic model [49].

The flux resolution obtained in each of the six independent LEs could clearly be improved via PLE-based flux analysis if two or more LEs were combined in the PLE. The corresponding calculations for the possible pair combinations are provided (see Additional file 3: Table SF-3.10), which resulted in the conclusion that the combination of [2-^13^C]-glucose and [1-^13^C]-glucose provided the most precise fluxes. In contrast, the use of [2-^13^C]-glucose and [5-^13^C]-glucose as the tracers in two independent LEs provided the least precise fluxes:

10 $$ {\varSigma}_{\eta (0.95)}{\left(\mathit{\mathsf{i}},\mathit{\mathsf{j}}\right)}_{PLE}\in \left[39.0{\left({}_{j=5}^{i=2}\right)}_{PLE},43.6{\left({}_{j=2}^{i=1}\right)}_{PLE}\right] $$

The results concerning the flux resolution obtained in experiments using singly labeled tracers demonstrated the model specificity of the experimental design optimization. Indeed, [4-^13^C]-glucose was one of the best tracers for the resolution of a large portion of the fluxes for the assumed *C. glutamicum* model and was one of the worst tracers for determining precise fluxes in the *E. coli* model [58]. Furthermore, the best detected pair combination ${\left({}_{j=2}^{i=1}\right)}_{PLE}$ for the resolution of fluxes in the model applied in the present study differed from the ${\left({}_{j=3}^{i=2}\right)}_{PLE}$ combination, which is best for *E. coli* [58].

In full accordance with the published data on the *E. coli* model [58], a significant improvement of the flux resolution was detected under the COMPLETE-MFA approach, where all six singly ^13^C-labeled glucose isotopomers were used separately as tracers in the individual LEs combined in the PLE (Figure 6: Column 13). Indeed, the computed value of the normalized flux precision function for all fluxes was as follows:

11 $$ {\varSigma}_{\eta (0.95)}{\left(1,2,3,4,5,6\right)}_{PLE}=45.9 $$

The flux resolution detected in this PLE was the best among the above-described resolutions obtained in the present study. It was clear that this result was obtained through PLE-mediated ^13^C-MFA due to the synergy of complementary information concerning the highly efficient resolution of the set of fluxes from the different parts of the metabolic model and the dependence of these sets on the different tracers used in individual LEs. Performing partial optimization of the experimental design for individual LEs for further PLE-based ^13^C-MFA it seemed probable to improve the flux resolution.

To test this possibility, partial optimization was conducted by searching the minimal linear approximation of free flux variances in the triangle composed of all possible mixed tracers, which consisted of the three singly labeled glucose isotopomers ([1-^13^C], [3-^13^C], and [4-^13^C]) that previously resulted in the best flux resolution according to the *Σ*_*η*_ -based criterion (see Additional file 3: **Exp. 9**). The obtained results are presented in Additional file 2: Figure SF-2.7. The set of minimal free flux variances could be observed to correspond closely to the corners of the tested triangle (e.g., for fluxes *θ*_4_, *θ*_14_, *θ*_22_, see Additional file 2: Figure SF-2.7 (B), (D), and (F), respectively), and three minima were detected approximately at the middle positions of the triangle’s sides (for fluxes *θ*_8_, *θ*_10_, *θ*_26_, see Additional file 2: Figure SF-2.7 (C), (E), and (G), respectively). It was interesting to mention, that all singly labeled glucose isotopomers used in these calculations could be considered as “partially optimized” for some free fluxes in the current metabolic model. According to this partial optimization, two PLEs were designed. The first consisted of three LEs with separately applied singly [1-, 3-, or 4-^13^C] labeled glucose. The second PLE consisted of six LEs, where, in addition to the three LEs from the previous PLE, three LEs used the optimized mixed paired combinations of glucose isotopomers. The corresponding PLEs were simulated, and ^13^C-MFA was performed, resulting in solutions of NLLSPs with highly resolved fluxes (Figure 7 (D) and (E)). Concerning the PLE consisting of three LEs (Figure 8: Column 10), the corresponding value of *Σ*_*η*(0.95)_(1, 3, 4)_*PLE*_ = 44.6 Additional file 3: (Table SF-3.10) significantly exceeded the best parameters obtained for the pairs of tracers, and was more than earlier obtained for PLEs from 5LEs with another tracers (see previous item). In turn, the flux precision obtained in the second PLE (Figure 8: Column 12) was approximately identical (maybe even slightly exceeded) to the previously described result for COMPLETE-MFA performed with six different singly labeled isotopomers of glucose (Additional file 3:Table SF-3.10**,** Figure 8: Column 11):

12 $$ {\varSigma}_{\eta (0.95)}\left(6\cdot ParOpt-mi{x}_{PLE}\right)=46.1 $$

Notably, an exceptionally high flux resolution was obtained in both PLEs including 6 LEs. For all fluxes, *η*_0.95_(*u*_*i*_) > 0; i.e., the length of the 95% confidence interval did not exceed the value of the corresponding flux corrected by the scaling factor.

## Conclusions

The main aim of the present study was to extend the possibilities of the previously developed open-access OpenFLUX software for comprehensive ^13^C-MFA. These extensions included (*i*) fitting the obtained experimental data, not only in SLEs but also in PLEs, to the assumed metabolic model; (*ii*) computing the flux parameters and providing the goodness-of-fit of the model’s adequacy, followed by an estimation of the model’s viability and its probable improvement; (*iii*) fine-tunable and convergence-controlled Monte Carlo-based approach to obtain distribution of optimized flux estimations followed by precise computing of flux confidence intervals; and (*iv*) conducting general and/or partial experimental design through searching for the minimal value to characterize the average confidence interval length for all free fluxes and/or the minimal linear approximation of the targeted free flux variances, respectively. The considered examples demonstrated the specific features of these steps and their concerted essentiality for obtaining statistically verified results of the described ^13^C-MFA provided with the help of OpenFLUX2.

Introducing the normalized flux precision function, *η*_*γ*_(*u*_*i*_; *β*), allowed for the quantitative characterization of the efficiency of the flux resolution at a confidence level of *γ*, with values of *η* close to “1” or “0” being obtained for efficiently or poorly resolved fluxes, respectively, depending on *β* as the scaling parameter, in particular. Moreover, the sum of *η* for all of the evaluated fluxes in the model, *Σ*_*η*_, could be a rather convenient parameter for conditional comparison of the flux precision achieved in different experiments, i.e., depending on the tracer(s) used.

The goodness-of-fit test of the assumed metabolic model’s adequacy is an essential and extremely important part of the statistically verified solution of the NLLSP. This test involved not only obtaining the *χ*^2^ -statistically acceptable value of the Ξ(**x**^*input*^, **x**^*mea*^, **σ**^*mea*^, **θ**) objective function but also confirmation of the expectation concerning the $\mathrm{N}\left(0,1\right)$ distribution of its summands. Providing an insufficient goodness-of-fit test could result in mistaken flux estimations, and the computed contribution matrix could be helpful for improving the statistical properties of the obtained solutions. It is desirable to retain the summands corresponding to the most important measurements if some of the “outstanding” variance-weighted residuals must be deleted to satisfy the normalization criterion.

The assumed metabolic model clearly influences the optimization of the experimental design and, ultimately, the precision of the flux estimation. Nevertheless, according to comparative calculations, the complementary parallel experimental labeling technique for metabolic flux analysis (COMPLETE-MFA [58]) using a set of different labeled tracers, e.g., singly ^13^C-labeled glucose isotopomers or combinations of mixed labeled tracer(s), chosen according to the partial optimization of the targeted fluxes, usually resulted in better flux precision compared with the resolution of fluxes computed from SLE data, which were provided according to a sophisticated general experimental design strategy. Only one simplified metabolic model, consisting of an l-lysine-producing *C. glutamicum* strain, was used for the simulation of experimental data and for comprehensive ^13^C-MFA in the present study; however, the obtained general conclusions coincided well with the data reported by other groups working with other models (e.g., [48,58]).

We hope that the developed OpenFLUX2 open-access software adjusted for comprehensive ^13^C-MFA of SLE and PLE data will help to broaden investigations aimed at quantitatively estimating the cellular metabolic state with high precision.

OpenFLUX2 is being released as open-source software. Regarding the citation of the OpenFLUX2 application, it is highly recommended that the present paper be cited, adding that this software is an extended version of the OpenFLUX open-source software described in [33].

## Methods

### *In silico* experiments

#### Metabolic and isotopomer balance models

The *C. glutamicum* metabolic model used as an example included catabolic reactions of the central metabolism, such as the Embden-Meyerhof-Parnas (EMP) and pentose phosphate (PP) pathways, the tricarboxylic acid (TCA) cycle, anaplerotic carboxylation, and the decarboxylation reaction of oxaloacetate and malate. Moreover, the pathways for the biosynthesis and transport of l-lysine and different extracellular co-products (glycine, trehalose, lactate, and α-ketoglutarate) were included. For glycine synthesis, two possible pathways, starting from serine and threonine [75,76], were considered. The glyoxylate bypass (shunt) was assumed to be inactive [39]. Pools of pyruvate/PEP or oxaloacetate/malate were lumped, followed by the expression of reactions catalyzed by PEP/PYR carboxylase or by PEP carboxykinase/malic enzyme as an irreversible reaction. To achieve accurate accounting of CO_2_-associated carbon transfer, the reactions, accompanied by CO_2_ production or consumption, were expressed in an explicit manner, including an anabolic reaction and a reaction involving CO_2_ exchange with environment. Additionally, the linear consequence of the irreversible reactions was represented as a single reaction. The forward and reverse components of a bi-directional reaction were considered as two non-negative fluxes. The biosynthetic pathways for the following amino acids were expressed explicitly: (*i*) the amino acids whose mass isotopomer distribution (MID) was assumed to be measured and (*ii*) the amino acids whose synthesis was accomplished by CO_2_ release. In Figure 2, for simplicity, the amino acid biosynthetic pathways are expressed schematically as a drain of the precursors to amino acids. Anabolic demand is represented in a slightly different manner than in previous studies [33,68]. Specifically, a single biomass equation was designed by presenting the biomass composition of *C. glutamicum* [75] as the sum of the amino acids whose biosynthesis was expressed explicitly and the residual amounts of the precursors drained to produce biomass. The reactions involved in alanine, aspartate, and glutamate synthesis were used to map the MID of the metabolite onto the MID of the corresponding amino acid (S-type reactions according OpenFLUX(2) notation), whereas the anabolic demand to synthesize these amino acids was considered through precursors. The anabolic demand for lysine included both the lysine used in protein synthesis and the diaminopimelate used in cell wall synthesis, as previously described [75].

The resulting metabolic model contained 54 reactions and 36 balanced metabolites. The reactions, which only map the labeled distribution of the metabolite onto the corresponding amino acid (e.g., pyruvate to alanine), did not participate in the stoichiometric balance. Thus, the stoichiometric matrix **S**, dim(**S**) = (36 × 51), with 36 balanced metabolites, 51 unknown fluxes, and *r* = rank(**S**) = 36, was finally generated. As a result, 15 fluxes should be assigned as free. Seven fluxes were experimentally determined effluxes, which included biomass biosynthesis (1 flux), the effluxes of secreted products (5 fluxes), and the glucose uptake rate (1 flux). Unless otherwise stated, these fluxes were considered free fluxes constrained according to Eq. (*S* − 1.2.2) with the experimentally determined parameters ${\mathbf{V}}_{eff}^{mea},{\boldsymbol{\upsigma}}_{eff}^{mea}$; thus, these fluxes subsequently formed the corresponding residuals in the $SS{R}_{\mathbf{f}}^{SLE}$ objective scalar-function Eq. (*S* − 1.3.7), which was subjected to a least-squares minimization procedure. Five free fluxes were automatically assigned due to their accordance to the reverse reactions: (*i*) in the non-oxidative branch of the PP pathway (3 fluxes), (*ii*) catalyzed by glucose-6-phosphate isomerase (1 flux), and (*iii*) for the carbon dioxide intracellular exchange (1 flux). The three remaining free fluxes were previously automatically assigned using OpenFLUX software [33]. There were fluxes corresponding to irreversible reactions catalyzed by glucose-6-phosphate dehydrogenase in the PP pathway, PEP/PYR carboxylase in the PEP-PYR-oxaloacetate node, and glycine synthesis in the serine-glycine biosynthetic pathway.

An isotopomer model was automatically built based on the EMU approach that simulated the MIDs of target compounds from the known MIDs of input substrates. The input substrates were specifically labeled glucose and naturally labeled CO_2_ (^13^C isotope abundance of 1.07%). In total, 17 sets of matrix equations of EMU balances were used to calculate the 107 unknown EMUs from 15 known EMUs of input substrates. The application of the EMU approach reduced the number of unknown variables from 9,138 unknown scalar variables in the full isotopomer model to 360 scalar MID variables, corresponding to 107 unknown EMUs.

#### Data used for estimating true flux values in the assumed model

Initially, the calculation of “true” flux values for the assumed metabolic model was performed using experimental data on effluxes and MIDs, together with their variances, available from the literature [33,68]. The published labeling patterns of the proteinogenic amino acids were determined due to GC-MS-mediated selective ion monitoring of selected ion clusters, representing [M-57] fragments with the complete carbon skeletons of the amino acids. The simulated MIDs of a compound with *n* carbon backbone atoms was represented by a mass distribution column vector (MDV), whose elements corresponded to the fractional abundances (${x}_{m_0+i}$) of mass isotopomer *m*_0_ + *i* (*i* = 1, 2, …, *n*), with ${\displaystyle \sum_{i=0}^n{x}_{m_0+i}}=1$, where *m*_0_ is the molecular weight of an unlabeled compound. As the published MIDs were uncorrected in accordance with the natural mass isotopomer abundance, all the simulated EMU variables were modified for mass interference from non-carbon backbone isotopes in dependence on the chemical structure of the derivatized substance according to the method developed in [77]. Then for each fragment, the first (*n* + 1) simulated mass isotopomers (*m*_0_, *m*_1_, …, *m*_*n*_) were normalized to unit followed by truncation of the isotopomers number to the dimension of the ${\mathbf{x}}_{MID}^{mea}$ vector before performing least-square analysis. The measured MIDs $\dim \left({\mathbf{x}}_{MID}^{mea}\right)=\left(3\times 1\right)$, i.e., only (*m*_0_, *m*_1_, *m*_2_) were presented for derivatized mass isotopomers fragments of Ala-260, Val-288, Thr-404, Asp-418, Glu-432, Ser-390, Phe-336, Tyr-466, Tre (trehalose)-361 isotopomers, and MIDs $\dim \left({\mathbf{x}}_{MID}^{mea}\right)=\left(2\times 1\right)$, for (*m*_0_, *m*_1_) of Gly-246. At the same time, an extremely small MID variance of 0.15% for mass isotopomer fractional abundances was indicated in [68] and accepted in [33], which ultimately led to rather large and statistically non-acceptable values of the objective function, i.e., the variance-weighted sum of squared residuals, calculated at the point of convergence ($\Xi \left(\overset{\frown }{\boldsymbol{\uptheta}}\right)\equiv 2\cdot SS{R}_{\mathbf{f}}^{SLE}\left(\overset{\frown }{\boldsymbol{\uptheta}}\right)$, according to the notations of the present publication (see Additional file 1: ***SF-1.3.***). These MID variances were assumed to be significantly underestimated. Indeed, the minimal published GC-MS measurement errors are usually approximately 0.2-0.4 mol% [78], and values of this magnitude have been used for variance weighting in studies where ^13^C-MFA has been applied [53,58,79]. To achieve statistically acceptable model fitting for the MIDs measured in [68] and to demonstrate all of the essential stages of the statistical analysis of the solution to the constrained NLLSP provided by the designed OpenFLUX2 software, the MID variances were assumed to be equal to 0.15 mol% for mass isotopomer fractional abundances (instead of 0.15%, as in [68]).

#### Generation of new “experimental data”

To generate new “experimental data” for *in silico* LEs, the OpenFLUX(2) forward simulation option was used, which calculated the MIDs of selected EMU variables from the assumed metabolic and isotopomer networks as well as the known label states of input substrates (^13^C-tracer(s)) and the assumed values of the fluxes. Unless otherwise stated, the true flux values, **u**_*true*_(**θ**_*true*_), were used for direct “experimental” data simulation in the present study. An isotopic purity of 99% was assumed for all of the specifically labeled carbons in the input substrates, and natural enrichment (1.07%) was assumed for other carbons. Again, the simulated MIDs of a substance with *n* carbon backbone atoms were represented by MDV with (*n* + 1) elements, ${x}_{m_0+i},i=1,\kern0.5em 2,\kern0.5em \dots, \kern0.5em n{\displaystyle \sum_{i=0}^n{x}_{m_0+i}}=1$. The natural mass isotopomer abundance of non-carbon-backbone atoms was generated according to the method [77] that resulted in significant increasing of the elements number in MDV for each tested substance, but OpenFLUX2 automatically truncated these mass isotopomers up to the first (*n* + 1) (with or without their normalization according to the user’s choice) (Additional file 3: Table SF-3.2). Then, mass isotopomer fractional abundances of lower than 0.04 (i.e., ≤ 4 mol%) from the total of 1 (i.e., 100 mol%) for each derivatized fragment were excluded from the set of simulated “experimental data,” thus modeling the limited sensitivity of the MS equipment. It is important to mention, that this procedure of the experimental data generation resulted in dependence of ${\mathbf{x}}_{MID}^{mea}$ vector dimension for each “measured” substance not only on its specific number of carbon backbone atoms, but on the different ^13^C-labeled tracers used in the simulated experiments. At the final stage of “experimental” MID generation, mass isotopomer fractional abundances were corrupted with $\mathrm{N}\left(\mathbf{0},{\boldsymbol{\upsigma}}_{MID}^{mea}\right)$ distributed noise, where ${\left\{{\boldsymbol{\upsigma}}_{MID}^{mea}\right\}}_i=0.4\left[\mathrm{mol}\%\right],i=1,2,\dots, {w}_{MID}$. As a result, a set of non-normalized, noisy MS “experimental data,” ${\mathbf{m}}_{MID}^{mea}$, that were corrected for the presence of natural isotopes, was generated for each LE according to the described procedure.

Another type of “experimental data” used during the *in silico* experiments was measured effluxes, ${\mathbf{V}}_{eff}^{mea}$. To generate these data, the “true” value of each efflux was corrupted with noise distributed as $\mathrm{N}\left(0,{\left\{{\boldsymbol{\upsigma}}_{eff}^{mea}\right\}}_j\right)$, where ${\left\{{\boldsymbol{\upsigma}}_{eff}^{mea}\right\}}_j$ was the experimentally determined standard deviation for each *j*-th efflux [68]. As a result, the full set of the “experimental data,” $$ {\mathbf{m}}^{mea}=\left(\begin{array}{l}{\mathbf{m}}_{MID}^{mea}\\ {}{\mathbf{V}}_{eff}^{mea}\end{array}\right) $$, was generated for the *in silico*-simulated LE.

#### Flux estimation and statistical analysis

Flux estimation, identifiability, and goodness-of-fit analyses were performed using OpenFLUX2 software. To determine the global minimum of the constrained NLLSP, as a rule, 100 independent iterative trials, starting from randomly selected points from a feasible constrained domain in the free flux variation space, ℜ^*p*^, were applied. In special cases, e.g., when the minimal value of the Ξ function was achieved in only a few trials, the number of iterative trials was increased to 300 to verify the detected global minimum. Only those iterative trials were used for analysis, which were terminated by satisfaction of the termination criteria without constraint violation (see Additional file 1: ***SF-1.3.***).

Monte Carlo-based approach included previously in OpenFLUX, but significantly modified in the OpenFLUX2 software for convenience of the computation process tuning and control of convergence, was used for estimation of the flux confidence intervals. Usually the most reliable results could be obtained if the variant when “multi runs per trials” approach was chosen for estimation of the optimized flux parameter distributions followed by determination of flux confidence interval borders according to “discarding” strategy after confirmation of the borders convergence (see Additional file 1: ***SF-1.7.*** for details).

### Software requirements

OpenFLUX2 (http://sourceforge.net/projects/openflux2) requires Java and MATLAB, including the Optimization and Statistics Toolboxes. The current version of the OpenFLUX2 software was tested using Java 6 (Sun Microsystems, Inc., Santa Clara, CA, USA) and MATLAB 7.12.0 (R2011a; MathWorks Inc., Natick, MA, USA) software, together with the Optimization Toolbox (version 6.0) and the Statistics Toolbox (version 7.5), on the Microsoft Windows 7 Professional (2009; Microsoft Corp, Redmond, WA, USA) platform on a PC equipped with a 3.2 GHz CPU and 4 Gb of RAM. Using these computation facilities, the calculation of 51 fluxes for the applied metabolic model (see Methods) requires approximately 20 minutes and 2 hours for SLEs and a PLE consisting of 5 LEs, respectively. The confidence interval estimations for the 51 optimized fluxes using the Monte Carlo approach (see Methods) takes approximately 30–70 and 60–120 hours for the SLEs and the PLE noted above, respectively. The computation time significantly depends on the used tunable control parameters and linearly depends on the number of the provided trials. In general, the flux estimation and its statistical analysis via OpenFLUX2 can be performed within one to four days, depending on the type of LE. OpenFLUX2 was also tested on the Microsoft Windows XP (Professional × 64 edition, 2003; Microsoft Corp, Redmond, WA, USA) platform. The following additional software packages were used: Windows Microsoft Excel 2003 (Microsoft Corp, Redmond, WA, USA) for metabolic model formulation and for the generation of Additional file 3 and OriginPro 9.1 (Originlab Corp, Northampton, MA, USA) for the generation of ternary plots, and box charts. Furthermore, several in-house MATLAB scripts were employed to generate “experimental data” and to visualize data related to the NLLSP solving process.

## Additional files

Additional file 1:
***General description of*** ^***13***^ ***C-MFA steps performed by OpenFLUX2.*** The PDF-format file contains the schematic description of the mathematical and statistical approaches used for comprehensive metabolic flux analysis via OpenFLUX2 software. Additional file 2:
***Additional figures illustrated results of performed in silico LEs.*** The PDF-format file contains additional illustrations of the *in silico* experimental results, denoted as Figure SF-2.x in the text. Additional file 3:
***C. glutamicum metabolic model.*** The XLS- format file includes the *C. glutamicum* metabolic model in a format that is readable by OpenFLUX(2); the “experimental data” (MIDs and efflux measurements with their errors) used in the *in silico* experiments; the main results of the *in silico* experiments, including the estimated optimal flux values, confidence intervals and normalized flux precision function values; and the results of the “general” and “partial” optimization of the experimental design.
