# Supplementary material for: OpenFLUX2: 13C-MFA modeling software package adjusted for the comprehensive analysis of single and parallel labeling experiments
Source: Microb Cell Fact. 2014 Nov 19;13:152. doi: 10.1186/s12934-014-0152-x (PMC4263107; doi:10.1186/s12934-014-0152-x)
Supplement: Additional file 1: — General description of 13 C-MFA steps performed by OpenFLUX2. The PDF-format file contains the schematic description of the mathematical and statistical approaches used for comprehensive metabolic flux analysis via OpenFLUX2 software. [file 12934_2014_152_MOESM1_ESM.pdf]

Supplementary File 1.

OpenFLUX2: ^{13}C-MFA modeling software package adjusted for the comprehensive analysis of single and parallel labeling experiments.

Mikhail S Shupletsov[1,2,†] • Lyubov I Golubeva[1] • Svetlana S Rubina[1] • Dmitry A Podvyaznikov[1,3] • Shintaro Iwatani[1,††] • Sergey V Mashko[1,3,4,†]

[1]*Ajinomoto-Genetika Research Institute, 117545, Moscow, Russian Federation;*

[2]*Computational Mathematics and Cybernetics Department, Lomonosov Moscow State University, 119991, Moscow, Russian Federation;*

[3]*Department of Theoretical and Experimental Physics, Moscow Physical Engineering Institute (Technical University), 115409, Moscow, Russian Federation;*

[4]*Biological Department, Lomonosov Moscow State University, 119991, Moscow, Russian Federation;*

[‡]Correspondence: sergey_mashko@agri.ru

[††]Present address: *Fermentation Group, Process Industrialization Section, Research Institute for Bioscience Products & Fine Chemicals, Ajinomoto Co., Inc., 840-2193, SAGA, Saga-shi, Morodomi-cho, 450 Morodomitsu, Japan*

Basic symbols:

a, b, c – scalar values
$\mathbf{u}, \mathbf{v}, \mathbf{w}$ – vectors
u_i, resp. $\{\mathbf{u}\}_i$ or $(\mathbf{u})_i$ – the i-th element of vector $\mathbf{u}$
$\mathbf{A}, \mathbf{B}, \mathbf{C}$ – matrices
a_{ij}, resp. $\{\mathbf{A}\}_{ij}$ or $(\mathbf{A})_{ij}$ – the i, j-th element of matrix $\mathbf{A}$
$\{\mathbf{A}\}_{i.}$ – the i-th row of matrix $\mathbf{A}$
$\{\mathbf{A}\}_{.j}$ – the j-th column of matrix $\mathbf{A}$
$\mathbf{u}^{\mathrm{T}}, \mathbf{A}^{\mathrm{T}}$ – the transpositions of $\mathbf{u}$ and $\mathbf{A}$
$\dim(\mathbf{u}), \dim(\mathbf{A})$ – the dimension of $\mathbf{u}$ and $\mathbf{A}$
$\mathrm{rank}(\mathbf{A})$ – the rank of $\mathbf{A}$
$\mathrm{Null}(\mathbf{A})$ – the null space of $\mathbf{A}$
$\mathrm{E}[x], \mathrm{E}[\mathbf{x}]$ - the expected value of random variable x and random vector $\mathbf{x}$, respectively
$\Sigma_{\mathbf{x}}$ - the covariance matrix of $\mathbf{x}$
$\mathrm{N}(\boldsymbol{\mu}, \Sigma)$ - multivariate normal distribution with mean vector $\boldsymbol{\mu}$ and covariance matrix Σ
$\mathrm{N}(0,1)$ - standard normal distribution
$\mathbf{J}_{\mathbf{f}}(\widehat{\boldsymbol{\theta}})$ - the Jacobian matrix that equals $\partial\mathbf{f}(\boldsymbol{\theta})/\partial\boldsymbol{\theta}$ evaluated at $\boldsymbol{\theta} = \widehat{\boldsymbol{\theta}}$
$\mathbf{H}_{SSR}(\widehat{\boldsymbol{\theta}})$ - the Hessian matrix of the scalar function SSR with respect to variables $\boldsymbol{\theta}$, evaluated at $\boldsymbol{\theta} = \widehat{\boldsymbol{\theta}}$
χ^2 – chi-squared distribution
$\chi^2_{1-\alpha}(p)$ – the value of the inverse of the cumulative distribution function of the χ^2 with p degrees of freedom at a significance level of α
$\chi^2_{\gamma}(p)$ – the same value as previous, but at a confidence level of γ, where $\gamma = 1 - \alpha$.

CONTENT

SF-1.1. Stoichiometric model and assignment of free fluxes 4

SF-1.2. Flux inequality constraints 8

SF-1.3. Flux calculations using regression analysis 11

SF-1.4. Statistical tests of model adequacy 17

SF-1.5. Local linearized statistical approximations 20

SF-1.6. Nonlinear search of flux confidence intervals 25

SF-1.7. Determination of flux confidence intervals using a Monte-Carlo-based approach 31

SF-1 Appendix 1. The special form of the null-space matrix in OpenFLUX(2) 40

SF-1 Appendix 2. Newton's method of the constrained NLLSP solution 45

SF-1 Appendix 3. Summing elements in an arbitrary row of the contribution matrix 48

References cited in Supplementary File 1 49

SF-1.1. Stoichiometric model and assignment of free fluxes

In ^{13}C-MFA, a stoichiometric model is based on a mass balance equation for metabolic quasi-steady state conditions:

$$\mathbf{S}\cdot\mathbf{v}=\mathbf{0}\,, \qquad (S-1.1.1)$$

where $\mathbf{S}$ is a $(m\times n)$-dimensional, i.e., $\dim(\mathbf{S})=(m\times n)$, stoichiometric matrix with $s_{i,j}$ coefficient of the i-th, $i=1, 2, \ldots, m$, balanceable metabolite in the j-th, $j=1, 2, \ldots, n$, reaction, and $\mathbf{v}=(v_1, v_2, \ldots, v_n)^{\mathrm{T}}$ is a flux column-vector, $\dim(\mathbf{v})=(n\times 1)$. The stoichiometric model is automatically generated by OpenFLUX(2) from the user-supplied reaction data.

Typically, realistic models are underdetermined because the number of fluxes normally exceeds the number of metabolites $(m<n)$ [S-1, S-2]. Under this assumption, the general solution to Eq. $(S-1.1.1)$ can be found e.g., using the method of Gauss-Jordan elimination and partial pivoting that result in the conversion of the $\mathbf{S}$ matrix with $r=\mathrm{rank}(\mathbf{S})\le m$ to the following form:

$$\mathbf{S}'=\begin{pmatrix}\mathbf{I} & \mathbf{M}\\ \mathbf{0} & \mathbf{0}\end{pmatrix}, \qquad (S-1.1.2)$$

where $\mathbf{I}$ denotes the identity matrix, $\dim(\mathbf{I})=(r\times r)$, and $\mathbf{M}$ is the matrix $\dim(\mathbf{M})=(r\times p)$ with $p=(n-r)$, which is obtained from the corresponding part of $\mathbf{S}$ after elementary row operations and after column rearrangements with the subsequent reordering of the variables in $\mathbf{v}$. Finally, the $\mathbf{u}$ flux column-vector, $\dim(\mathbf{u})=(n\times 1)$, is obtained:

$$\mathbf{u}=\begin{pmatrix}\mathbf{v}_{dep}\\ \boldsymbol{\theta}\end{pmatrix}, \qquad (S-1.1.3)$$

where the upper elements $\mathbf{v}_{dep}$, $\dim(\mathbf{v}_{dep}) = (r \times 1)$, and the lower elements $\boldsymbol{\theta}$, $\dim(\boldsymbol{\theta}) = (p \times 1)$, are assigned as "dependent" and "free" fluxes, respectively. The following equation is derived from Eqs. $(S-1.1.1)$ – $(S-1.1.3)$:

$$\mathbf{S}' \cdot \mathbf{u} = \begin{pmatrix} \mathbf{I} & \mathbf{M} \\ \mathbf{0} & \mathbf{0} \end{pmatrix} \cdot \begin{pmatrix} \mathbf{v}_{dep} \\ \boldsymbol{\theta} \end{pmatrix} = \mathbf{0}. \qquad (S-1.1.4)$$

From Eq. $(S-1.1.4)$, according to their assignment, the "dependent" fluxes could be expressed as a linear combination of the "free" fluxes as follows:

$$\mathbf{v}_{dep} = -\mathbf{M} \cdot \boldsymbol{\theta}. \qquad (S-1.1.5)$$

Assigning $\mathbf{N} = \begin{pmatrix} -\mathbf{M} \\ \mathbf{I} \end{pmatrix}$, where $\dim(\mathbf{I}) = (p \times p)$ and $\dim(\mathbf{N}) = (n \times p)$, the equation for the flux calculation is finally obtained as follows:

$$\mathbf{u} = \begin{pmatrix} \mathbf{v}_{dep} \\ \boldsymbol{\theta} \end{pmatrix} = \begin{pmatrix} -\mathbf{M} \\ \mathbf{I} \end{pmatrix} \cdot \boldsymbol{\theta} = \mathbf{N} \cdot \boldsymbol{\theta}. \qquad (S-1.1.6)$$

Thus, the general solution to the stationary Eq. $(S-1.1.4)$ can be expressed as a linear combination of $p = (n-r)$ independent $\boldsymbol{\theta}$ fluxes (Eq. $(S-1.1.6)$), where $\mathbf{N}$ is the null-space matrix of $\mathbf{S}$, $\mathbf{N} \equiv \mathrm{Null}(\mathbf{S})$, [S-3, S-4]. In general, the forms of $\mathbf{N}$ and the corresponding sets of $\boldsymbol{\theta}$ are not unique for any given $\mathbf{S}$, and different methods can be applied for their calculation [S-5]. Nevertheless, $\dim(\mathbf{N})$ and $\dim(\boldsymbol{\theta})$ are fully determined by the dimension and the rank of the $\mathbf{S}$ matrix.

The OpenFLUX(2) software essentially follows the above-described procedure of $\mathbf{N}$ and $\boldsymbol{\theta}$ calculation, with the following differences, which improve computational stability: First, matrix $\mathbf{N}'$ of the orthonormal basis vectors of the null space of $\mathbf{S}$ is obtained using singular value decomposition. Because matrix $\mathbf{N}$ is composed of vectors of a specific basis of the null space of $\mathbf{S}$, this matrix can be acquired from the $\mathbf{N}'$ matrix using the following linear

transforms. First, the $\mathbf{N}''$ matrix is obtained by swapping rows in $\mathbf{N}'$, according to the reordering of the initial flux column-vector. Finally, $\mathbf{N}$ is acquired from the row-reduced echelon form of $\mathbf{N}''$ using elementary row and column operations.

In OpenFLUX(2), the $\mathbf{v}$ flux column-vector is decomposed into the following four different reaction types: bi-directional forward and reverse reactions $(\mathbf{v}^{\rightarrow}$ and $\mathbf{v}^{\leftarrow})$ and irreversible reactions that are either free fluxes $(\boldsymbol{\theta}^{irr})$ or dependent fluxes $(\mathbf{v}_{dep}^{irr})$ [S-6]. Usually, the variable measured effluxes, $\mathbf{v}_{eff}^{mea}$, which are constrained within obtained error ranges, are automatically considered to belong to the set of $\boldsymbol{\theta}^{irr}$ [S-6, S-7], i.e., $\mathbf{v}_{eff}^{mea} = \boldsymbol{\theta}_{eff}^{irr} \subseteq \boldsymbol{\theta}^{irr} \subseteq \boldsymbol{\theta}$. According to the user's choice, the precisely determined effluxes could be considered to be invariant fluxes of the fixed values, i.e., constant parameters. Nevertheless, only the variable effluxes with the experimentally measured standard deviations would be considered in the present study to standardize all provided further computations with the free fluxes, their variances, elements of Jacobian and Hessian matrixes, *etc.*

Furthermore, some of the rest of the model-based fluxes can also be automatically included in the set of free fluxes. Indeed, let v_i and v_j, $1 \le i \ne j \le n$, be the elements of column-vector $\mathbf{v}$, which correspond to the forward and reverse fluxes, respectively, of one of the t bi-directional reactions. Then, the following equality of the stoichiometric coefficients must be accomplished for the arbitrary l-th metabolite, $1 \le l \le m$:

$$s_{li} = -s_{lj} . \qquad (S-1.1.7)$$

Thus, the i-th and j-th columns of the $\mathbf{S}$ matrix are linearly dependent, indicating that all t columns corresponding to $\mathbf{v}^{\leftarrow}$, for example, could be

rearranged to the right part of the $\mathbf{S}$ matrix at the stage of conversion to the form presented in Eq. $(S-1.1.2)$, with the coupled reordering of the position of $\mathbf{v}^{\leftarrow}$ to the bottom part of the nascent $\mathbf{u}$ column-vector in Eq. $(S-1.1.3)$. Thus, all t reverse fluxes would be assigned as free fluxes. The residual $(p-t)$ essential free fluxes must be chosen after finalizing the generation of $\mathbf{S}'$ in Eq. $(S-1.1.2)$. If all $\mathbf{v}^{\rightarrow}$ fluxes generate a linearly independent set, then Eq. $(S-1.1.6)$ and the expressions for $\mathbf{u}$, $\mathbf{N}$, and $\boldsymbol{\theta}$ can be presented in the following form:

$$\mathbf{u}=\begin{pmatrix}\mathbf{v}^{\rightarrow}\\ \mathbf{v}_{dep}^{irr}\\ \boldsymbol{\theta}^{irr}\\ \mathbf{v}^{\leftarrow}\end{pmatrix}=\begin{pmatrix}\mathbf{M}_{net}^{rev} & \mathbf{I}\\ \mathbf{M}_{net}^{irr} & \mathbf{0}\\ \mathbf{I} & \mathbf{0}\\ \mathbf{0} & \mathbf{I}\end{pmatrix}\cdot\begin{pmatrix}\boldsymbol{\theta}^{irr}\\ \mathbf{v}^{\leftarrow}\end{pmatrix}=\mathbf{N}\cdot\begin{pmatrix}\boldsymbol{\theta}^{irr}\\ \mathbf{v}^{\leftarrow}\end{pmatrix}=\mathbf{N}\cdot\boldsymbol{\theta}, \qquad (S-1.1.8)$$

corresponding calculations with introducing of the matrixes $\mathbf{M}_{net}^{rev}$ and $\mathbf{M}_{net}^{irr}$ definitions are presented in ***SF-1***. ***Appendix 1***.

All free fluxes must be chosen by the user or, alternatively, could be automatically assigned by OpenFLUX(2) (with the default inclusion of $\mathbf{v}^{\leftarrow}$ and $\boldsymbol{\theta}_{eff}^{irr}$ as a subset of $\boldsymbol{\theta}$). In the former case, the software toolboxes diagnose the user's choice of the $\boldsymbol{\theta}$ candidates and edit this set, if necessary, due to the computer-based determination of matrix $\mathbf{N}$ and to the corresponding set of free fluxes. The selected $\boldsymbol{\theta}$ candidates are verified for their ability to generate a feasible set of non-negative $\mathbf{u}$ fluxes according to Eq. $(S-1.1.6)$, which are subjected to the flux inequality constraints (see ***SF-1.2.***).

SF-1.2. Flux inequality constraints

Because every bi-directional reaction is presented by a pair of forward and reverse fluxes in OpenFLUX(2), the following obvious constraints must be applied to the calculation of fluxes:

$$\mathbf{u}(\boldsymbol{\theta}) = \begin{pmatrix} \mathbf{v}_{dep} \\ \boldsymbol{\theta} \end{pmatrix} \geq \mathbf{0}. \qquad (S-1.2.1)$$

In OpenFLUX2, $\boldsymbol{\theta} = \begin{pmatrix} \boldsymbol{\theta}^{irr} \\ \mathbf{v}^{\leftarrow} \end{pmatrix}$ values are non-negative and obey additional constraints. First, $\boldsymbol{\theta}^{irr}$ is upward bounded by the net flux in the system. Next, it is supposed [S-3, S-8] that a range is given for the variable $\boldsymbol{\theta}_{eff}^{irr} \in \boldsymbol{\theta}^{irr}$, which is measured with the normally distributed experimental error, and that this range depends on the following efflux confidence intervals:

$$\mathbf{lb}_{eff}^{irr} = \max\left\{\mathbf{0},\ \mathbf{q}_{eff}^{mea} - \boldsymbol{\sigma}_{eff}^{mea} \cdot \sqrt{\chi_{\gamma}^{2}(1)}\right\} \leq \boldsymbol{\theta}_{eff}^{irr} \leq \mathbf{q}_{eff}^{mea} + \boldsymbol{\sigma}_{eff}^{mea} \cdot \sqrt{\chi_{\gamma}^{2}(1)} = \mathbf{ub}_{eff}^{irr}, \qquad (S-1.2.2)$$

where $\mathbf{q}_{eff}^{mea}$ and $\boldsymbol{\sigma}_{eff}^{mea}$ are the experimentally obtained values of $\boldsymbol{\theta}_{eff}^{irr}$ and their measurement variances, respectively; and $\chi_{\gamma}^{2}(1)$ is the value of the inverse of the cumulative distribution function of the χ^2-distribution, with one degree of freedom at a confidence level of γ (i.e., $\gamma = 0.95$ for the 95% confidence level). Thus, $100 \times \gamma\,\%$ from all possible experimental observations of $\boldsymbol{\theta}_{eff}^{irr}$ would be dispersed in the range defined by Ineq. $(S-1.2.2)$.

For the residual (e.g., intracellular) non-negative irreversible free fluxes, $\boldsymbol{\theta}_{in}^{irr}$, the upper bound, $\mathbf{ub}_{in}^{irr}$, is, by default, 15 times higher than the maximum substrate uptake rate, $15 \times \max \mathbf{q}_{eff}^{mea}$ [S-6]. In OpenFLUX2, it is possible to augment the range of the $\boldsymbol{\theta}_{eff}^{irr}$ flux variation constrained by Ineq. $(S-1.2.2)$ up to the bounds typical for the $\boldsymbol{\theta}_{in}^{irr}$ fluxes. This option could be preferable for flux

calculations when the $\mathbf{q}_{eff}^{mea}$ and/or $\boldsymbol{\sigma}_{eff}^{mea}$ experimental parameters could be inadequately estimated.

In turn, the $\mathbf{v}^{\leftarrow}$ fluxes are non-negative but have no natural upper limit. Nevertheless, in OpenFLUX2, the user can assign an artificial upper limit for $\mathbf{v}^{\leftarrow}$ (e.g., $\max \mathbf{v}^{\leftarrow} = 200 \times \max \mathbf{q}_{eff}^{mea}$). Moreover, a compactification operation is always used by OpenFLUX(2) to transform a physical $\mathbf{0} \le \mathbf{v}^{\leftarrow} < \infty$ scale to a numerical $\mathbf{0} \le \mathbf{v}^{\leftarrow,[0,1)} < \mathbf{1}$ scale [S-9] to improve the calculation efficiency [S-3, S-8]. In this situation, $\mathbf{v}^{\leftarrow,[0,1)} = \dfrac{\mathbf{v}^{\leftarrow}}{\mathbf{P} + \mathbf{v}^{\leftarrow}}$ – is a conversion of $\mathbf{v}^{\leftarrow}$ to the new $\mathbf{v}^{\leftarrow,[0,1)}$ vector, where $\mathbf{P}$ denotes a vector of scaling factors with values that could be proportional to $\max \mathbf{q}_{eff}^{mea}$ [S-6, S-10]. Thus, in OpenFLUX2, the $\boldsymbol{\theta}$ flux-vector and its compactification analog, $\boldsymbol{\theta}^{comp} = \begin{pmatrix} \boldsymbol{\theta}_{eff}^{irr} \\ \boldsymbol{\theta}_{in}^{irr} \\ \mathbf{v}^{\leftarrow,[0,1)} \end{pmatrix}$, is constrained by lower and upper bounds. Indeed, the corresponding constraints for $\boldsymbol{\theta}$, in particular, could be the following:

$$\mathbf{0} \le \mathbf{lb}_{\boldsymbol{\theta}} = \begin{pmatrix} \mathbf{lb}_{eff}^{irr} \\ \mathbf{0} \\ \mathbf{0} \end{pmatrix} \le \boldsymbol{\theta} = \begin{pmatrix} \boldsymbol{\theta}_{eff}^{irr} \\ \boldsymbol{\theta}_{in}^{irr} \\ \mathbf{v}^{\leftarrow} \end{pmatrix} \le \begin{pmatrix} \mathbf{ub}_{eff}^{irr} \\ \mathbf{ub}_{in}^{irr} \\ \max\left(\mathbf{v}^{\leftarrow}\right) \end{pmatrix} = \mathbf{ub}_{\boldsymbol{\theta}}, \text{ i.e., } \boldsymbol{\theta} \in R^p \equiv [\mathbf{lb}_{\boldsymbol{\theta}}, \mathbf{ub}_{\boldsymbol{\theta}}], \qquad (S-1.2.3)$$

where R^p indicates a feasible domain in $p = (n - r)$-dimensional space of the $\boldsymbol{\theta}$ free flux variation.

Simultaneously, the inequality $\left(\mathbf{v}_{dep}(\boldsymbol{\theta}) = -\mathbf{M} \cdot \boldsymbol{\theta} \ge \mathbf{0}\right)$ can not always be satisfied for all $\boldsymbol{\theta} \in R^p$ [S-3]. Thus, the narrowed domain, $\Re^p \equiv [\mathbf{lb}_{\boldsymbol{\theta}}^*, \mathbf{ub}_{\boldsymbol{\theta}}^*] \subseteq R^p$, could formally be defined as a set of all $\boldsymbol{\theta} \in R^p$ for which only the non-negative values of the $\mathbf{v}_{dep}(\boldsymbol{\theta})$ fluxes are generated.

Let $\mathbf{e}_k = (e_{1,k}, e_{2,k}, \ldots, e_{r,k})^{\mathrm{T}}$ and $k = (1, 2, \ldots, r)$ be a set of column-vectors, $\dim(\mathbf{e}_k) = (r \times 1)$, where the $e_{i,k}$ entry of each $\mathbf{e}_k$ is the following:

$$e_{i,k} = \begin{cases} 1, \text{ if } i = k \\ 0, \text{ if } i \neq k \end{cases} \qquad (S-1.2.4).$$

Then, two linear programming problems must be solved to provide the dependent flux variability analysis, i.e., to establish the available lower and upper bounds ($\mathbf{lb}_{\mathbf{v}_{dep}}$ and $\mathbf{ub}_{\mathbf{v}_{dep}}$, respectively) for non-negative variables of the $\mathbf{v}_{dep}$ flux:

$$\begin{aligned} \left\{\mathbf{lb}_{\mathbf{v}_{dep}}\right\}_k &= \min\left\{\mathbf{e}_k^{\mathrm{T}} \cdot (-\mathbf{M}) \cdot \boldsymbol{\theta} \,\middle|\, \boldsymbol{\theta} \in \Re^p \subseteq R^p\right\} \geq 0, \\ \left\{\mathbf{ub}_{\mathbf{v}_{dep}}\right\}_k &= \max\left\{\mathbf{e}_k^{\mathrm{T}} \cdot (-\mathbf{M}) \cdot \boldsymbol{\theta} \,\middle|\, \boldsymbol{\theta} \in \Re^p \subseteq R^p\right\} = \min\left\{\mathbf{e}_k^{\mathrm{T}} \cdot \mathbf{M} \cdot \boldsymbol{\theta} \,\middle|\, \boldsymbol{\theta} \in \Re^p \subseteq R^p\right\} \geq 0. \end{aligned} \qquad (S-1.2.5)$$

Therefore, only $\boldsymbol{\theta}$ values that belong to the non-empty $\Re^p$ domain are finally considered by OpenFLUX2 (see ***SF-1.1.***). Thus, the range of the feasible variation of all fluxes, which is constrained by stoichiometric and experimental data, is established:

$$\mathbf{0} \leq \mathbf{lb} = \begin{pmatrix} \mathbf{lb}_{\mathbf{v}_{dep}} \\ \mathbf{lb}_{\boldsymbol{\theta}}^{*} \end{pmatrix} \leq \mathbf{u}(\boldsymbol{\theta}) = \begin{pmatrix} \mathbf{v}_{dep} \\ \boldsymbol{\theta} \end{pmatrix} \leq \begin{pmatrix} \mathbf{ub}_{\mathbf{v}_{dep}} \\ \mathbf{ub}_{\boldsymbol{\theta}}^{*} \end{pmatrix} = \mathbf{ub}, \text{ i.e., } \mathbf{u}(\boldsymbol{\theta}) \in \Re^n \equiv [\mathbf{lb}, \mathbf{ub}]. \qquad (S-1.2.6)$$

These constraints formally determine the feasible domain $\Re^n$ in n-dimensional space for all flux values estimated using the iterative fitting procedure (***SF-1.3.***) and for potential bounds of the flux confidence intervals (***SF-1.5.*** – ***SF-1.7.***). $\Re^p$ indicates a feasible domain in $p = (n - r)$-dimensional space of the $\boldsymbol{\theta}$ free flux variation. The direct establishment of the $\Re^n$ parameters is necessary for providing, probably, an optimal iterative fitting procedure with the consequently chosen uniformly distributed points $\mathbf{u}_i(\boldsymbol{\theta}_i)$, $i = 1, 2, \ldots, K$ for each from $K \geq 100$ trials. At present, the algorithm generating the uniformly

distributed points has not been implemented in OpenFLUX2 yet (but already realized in high-performance 13CFLUX2 software [S-11]). In OpenFLUX2, the random points $\boldsymbol{\theta}_i$, $i = 1, 2, ..., K$ from the feasible $\Re^p$-domain are chosen for the consequent trial, but only those $\boldsymbol{\theta}_i$ are used in the fitting procedure that satisfy the $(S-1.2.1)$ inequality.

SF-1.3. Flux calculations using regression analysis

Let $\mathbf{x}^{mea} = \begin{pmatrix} \mathbf{x}^{mea}_{MID} \\ \mathbf{v}^{mea}_{eff} \end{pmatrix}$, $\dim(\mathbf{x}^{mea}_{MID}) = (w_{MID} \times 1)$, $\dim(\mathbf{v}^{mea}_{eff}) = (w_{eff} \times 1)$, $\dim(\mathbf{x}^{mea}) = (w \times 1)$, where $w = (w_{MID} + w_{eff})$, be a variable column-vector of experimentally measured data (containing both labeling measurements, $\mathbf{x}^{mea}_{MID}$, and the effluxes $\mathbf{v}^{mea}_{eff}$) with the corresponding measurement noise vector $\boldsymbol{\delta}^{mea} = \begin{pmatrix} \boldsymbol{\delta}^{mea}_{MID} \\ \boldsymbol{\delta}^{mea}_{eff} \end{pmatrix}$. If $\langle \mathbf{x} \rangle$ is the unknown in reality true value, then:

$$\langle \mathbf{x} \rangle = \mathbf{x}^{mea} + \boldsymbol{\delta}^{mea} \qquad (S-1.3.1).$$

Usually (see e.g., [S-11]), it is assumed that the noise vector, $\boldsymbol{\delta}^{mea}$, is normally distributed with an expectation vector $\mathrm{E}[\boldsymbol{\delta}^{mea}] = \mathbf{0}$, where E is the expected value operator, with a measurement variance-covariance matrix, $\Sigma_{\mathbf{x}}$, which is expressed as follows:

$$\Sigma_{\mathbf{x}} \equiv \mathrm{E}[(\mathbf{x}^{mea} - \langle \mathbf{x} \rangle) \cdot (\mathbf{x}^{mea} - \langle \mathbf{x} \rangle)^{\mathrm{T}}] = \mathrm{E}[\boldsymbol{\delta}^{mea} \cdot (\boldsymbol{\delta}^{mea})^{\mathrm{T}}], \qquad (S-1.3.2)$$

i.e., $\boldsymbol{\delta}^{mea} \sim \mathrm{N}(\mathbf{0}, \Sigma_{\mathbf{x}})$.

Notably, the correct bias estimation and the removal of the background signals from “raw” MS-mediated data, in particular, are crucial for the

evaluation of $\mathbf{x}_{MID}^{mea}$, which is consistent with the assumed normality of the $\boldsymbol{\delta}_{MID}^{mea}$ distribution [S-12].

OpenFLUX(2) assumes individual measurements to be pair-wise, independent, and uncorrelated. Under these assumptions, $\Sigma_{\mathbf{x}}$ can be considered diagonal, with squared measurement-variances as diagonal elements as follows:

$$\begin{aligned} (\Sigma_{\mathbf{x}})_{i,i} &= \mathrm{E}[\delta_i^{mea} \cdot (\delta_i^{mea})^{\mathrm{T}}] = (\sigma_i^{mea})^2 \\ \Sigma_{\mathbf{x}} &= \Sigma_{\mathbf{x}}(\boldsymbol{\sigma}^{mea}) = \mathbf{I} \cdot (\boldsymbol{\sigma}^{mea})^2 \end{aligned} \qquad (S-1.3.3),$$

where $\mathbf{I}$ is the identity matrix $\dim(\mathbf{I}) = (w \times w)$, and $(\boldsymbol{\sigma}^{mea})^2 = \begin{pmatrix} (\boldsymbol{\sigma}_{MID}^{mea})^2 \\ (\boldsymbol{\sigma}_{eff}^{mea})^2 \end{pmatrix}$ is the column-vector of the squared measurement variances $\dim((\boldsymbol{\sigma}^{mea})^2) = (w \times 1)$.

Let $\mathbf{x}^{input}$ be a column-vector of the labeling data of input substrate(s) and $f_i(\mathbf{x}^{input}, \boldsymbol{\theta}),\ i = 1, 2, \ldots, w_{MID},$ is parameterized by the $\mathbf{x}^{input}$ function, which describes the relation between the free fluxes $\boldsymbol{\theta}$ and the i-th MID fraction of the intracellular metabolite (e.g., proteinogenic amino acids). Then, the following isotopomer balance model could be generated as a nonlinear $\mathbf{f}$ vector-function, which maps $\mathbf{x}^{input}$ and $\boldsymbol{\theta}$ to a column-vector of simulated measurements data $\mathbf{x}^{calc}$, $\dim(\mathbf{x}^{calc}) = (w \times 1)$):

$$\mathbf{x}^{calc} = \mathbf{f}(\mathbf{x}^{input}, \boldsymbol{\theta}) = \left(f_1(\mathbf{x}^{input}, \boldsymbol{\theta}), \ldots, f_{w_{MID}}(\mathbf{x}^{input}, \boldsymbol{\theta}), \{\boldsymbol{\theta}_{eff}^{irr}\}_1, \ldots, \{\boldsymbol{\theta}_{eff}^{irr}\}_{w_{eff}}\right)^{\mathrm{T}}. \qquad (S-1.3.4)$$

The resulting $\mathbf{x}^{calc}$ vector corresponds to the $\mathbf{x}^{mea}$ measurements vector. In OpenFLUX(2), $f_i(\mathbf{x}^{input}, \boldsymbol{\theta}),\ i = 1, 2, \ldots, w_{MID},$ is the value of an EMU-based function that describes the relation between fluxes and the i-th MID fraction of the intracellular metabolite, which is calculated using the decomposition algorithm,

with the sequential identification of all reactant EMUs that contribute to the i-th EMU product [S-13]. The nonlinearity of the generated $f_i(\mathbf{x}^{input}, \boldsymbol{\theta})$ dependent variables with respect to the flux is based specifically on the fact that the full set of equations contains isotopomer abundance obtained due to unimolecular (linear) and condensation (bilinear) EMU reactions in the system [S-4, S-14]. Therefore, the nonlinear flux-dependent coefficients for the expression of the targeted EMU MIDs contain convolution terms involving lower level EMU variables. The automatic generation of the isotopomer balance model according to the EMU decomposition framework [S-13] using the algorithms applied in OpenFLUX(2) has been described previously [S-6].

Based on the generated isotopomer balance model $\mathbf{f}$, the following $\boldsymbol{\varepsilon}$ vector function is parameterized by $\mathbf{x}^{input}$ and $\mathbf{x}^{mea}$, which map values of $\boldsymbol{\theta}$ to the vector of residual errors between calculated values and experimental data, respectively:

$$\boldsymbol{\varepsilon}(\mathbf{x}^{input}, \mathbf{x}^{mea}, \boldsymbol{\theta}) = \mathbf{x}^{calc}(\mathbf{x}^{input}, \boldsymbol{\theta}) - \mathbf{x}^{mea} = \mathbf{f}(\mathbf{x}^{input}, \boldsymbol{\theta}) - \mathbf{x}^{mea} . \qquad (S-1.3.5)$$

Then, the measurement-variance-weighted sum of squared residual errors is assigned as the following objective scalar-function of the $\boldsymbol{\theta}$ variables and is parameterized by $\mathbf{x}^{input}$, $\mathbf{x}^{mea}$ and $\boldsymbol{\sigma}^{mea}$:

$$SSR_{\mathbf{f}}^{SLE}(\mathbf{x}^{input}, \mathbf{x}^{mea}, \boldsymbol{\sigma}^{mea}, \boldsymbol{\theta}) = \frac{1}{2} \cdot \left(\boldsymbol{\varepsilon}(\mathbf{x}^{input}, \mathbf{x}^{mea}, \boldsymbol{\theta})\right)^{\mathrm{T}} \cdot \Sigma_{\mathbf{x}}^{-1}(\boldsymbol{\sigma}^{mea}) \cdot \boldsymbol{\varepsilon}(\mathbf{x}^{input}, \mathbf{x}^{mea}, \boldsymbol{\theta}), \qquad (S-1.3.6)$$

where $\Sigma_{\mathbf{x}}^{-1}$ is the inverse of the $\Sigma_{\mathbf{x}}$ matrix. In the case of the diagonal form of $\Sigma_{\mathbf{x}}$ (see Eq. $(S-1.3.3)$), the applied weighting procedure normalizes the squared residual errors between the observed and simulated measurements using the squared measurement variances $\left(\boldsymbol{\sigma}^{mea}\right)^2$. The coefficient "½" is usually introduced in the definition of the $SSR_{\mathbf{f}}^{SLE}$-like objective functions [S-3, S-8, S-

15] to compensate for the coefficient “2”, which appears when the squared objective function is differentiated (see ***SF-1 Appendix 2***). In contrast, to provide statistical tests for model adequacy, the $\mathrm{N}(0,1)$ normal distribution of the individual residuals and the correspondence of their sum to χ^2-distribution at the point of convergence would be necessary to verify the $SSR_{\mathbf{f}}^{SLE}$ -like objective function without ½ as the coefficient. Thus, the $\Xi \equiv 2 \cdot SSR_{\mathbf{f}}^{SLE}$ function would be particularly assigned for these purposes in ***SF-1.4***. Namely, this $\Xi(\mathbf{x}^{input}, \mathbf{x}^{mea}, \boldsymbol{\sigma}^{mea}, \boldsymbol{\theta})$ function (instead of $SSR_{\mathbf{f}}^{SLE}$) is assigned as an objective function in several publications (e.g., [S-6, S-16, S-17, S-18 or S-4] where the authors do not mention the Hessian matrix of the objective function or omit some details that are essential for its approximation; see ***SF-1 Appendix 2***).

Let $\mathbf{m}^{input}$ be a vector of $\mathbf{x}^{input}$ values, which has been used in the actual provided SLE, and $\mathbf{m}^{mea} = \begin{pmatrix} \mathbf{m}_{MID}^{mea} \\ \mathbf{q}_{eff}^{mea} \end{pmatrix}$ is an experimental realization of the $\mathbf{x}^{mea}$ vector in this SLE. According to Eqs. $(S-1.3.3)$, $(S-1.3.5)$, and $(S-1.3.6)$, the obtained value of the $SSR_{\mathbf{f}}^{SLE}$ objective function can be presented as follows:

$$SSR_{\mathbf{f}}^{SLE}(\mathbf{m}^{input}, \mathbf{m}^{mea}, \boldsymbol{\sigma}^{mea}, \boldsymbol{\theta}) = \frac{1}{2} \cdot \left(\sum_{i=1}^{w_{MID}} \left(\frac{f_i(\mathbf{m}^{input}, \boldsymbol{\theta}) - \left\{ \mathbf{m}_{MID}^{mea} \right\}_i}{\left\{ \boldsymbol{\sigma}_{MID}^{mea} \right\}_i} \right)^2 + \sum_{j=1}^{w_{eff}} \left(\frac{\left\{ \boldsymbol{\theta}_{eff}^{irr} \right\}_j - \left\{ \mathbf{q}_{eff}^{mea} \right\}_j}{\left\{ \boldsymbol{\sigma}_{eff}^{mea} \right\}_j} \right)^2 \right). \qquad (S-1.3.7)$$

The following <u>n</u>on<u>l</u>inear <u>l</u>east-<u>s</u>quares minimization <u>p</u>roblem (NLLSP) must be solved to estimate the optimized $\mathbf{u}(\boldsymbol{\theta})$ parameters, which are subjected to constraints:

$$\begin{cases} \min\limits_{\boldsymbol{\theta} \in \Re^p} SSR_{\mathbf{f}}^{SLE}(\mathbf{m}^{input}, \mathbf{m}^{mea}, \boldsymbol{\sigma}^{mea}, \boldsymbol{\theta}) \\ \mathbf{u}(\boldsymbol{\theta}) \in \Re^n \end{cases}. \qquad (S-1.3.8)$$

An analogous strategy can be used to calculate the optimized $\mathbf{u}(\boldsymbol{\theta})$ in the PLE, including k independent LEs. The following $\mathbf{z}$ -like vectors,

$\mathbf{z} = \left((\mathbf{z})_1^{\mathrm{T}}, (\mathbf{z})_2^{\mathrm{T}}, ..., (\mathbf{z})_k^{\mathrm{T}}\right)^{\mathrm{T}}$, can be considered for this PLE, where $\mathbf{z}$ denotes the nonlinear vector-function with entries $(\mathbf{z})_i$ corresponding to vector-function $\mathbf{f}_i$ of *i*-th LE (1 ≤ *i* ≤ *k*), with defined vectors $\mathbf{m}_i^{input}$, $\mathbf{m}_i^{mea}$, and $\boldsymbol{\sigma}_i^{mea}$. In the case of the independence of individual LEs in the analyzed PLE, the objective scalar-function of $\boldsymbol{\theta}$ with $\mathbf{m}^{input} = \left((\mathbf{m}_1^{input})^{\mathrm{T}}, (\mathbf{m}_2^{input})^{\mathrm{T}}, \ldots, (\mathbf{m}_k^{input})^{\mathrm{T}}\right)^{\mathrm{T}}$, $\mathbf{m}^{mea} = \left((\mathbf{m}_1^{mea})^{\mathrm{T}}, (\mathbf{m}_2^{mea})^{\mathrm{T}}, ..., (\mathbf{m}_k^{mea})^{\mathrm{T}}\right)^{\mathrm{T}}$ and $\boldsymbol{\sigma}^{mea} = \left((\boldsymbol{\sigma}_1^{mea})^{\mathrm{T}}, (\boldsymbol{\sigma}_2^{mea})^{\mathrm{T}}, ..., (\boldsymbol{\sigma}_k^{mea})^{\mathrm{T}}\right)^{\mathrm{T}}$, which are the vector parameters, could be presented in the following form:

$$SSR_{\mathbf{z}}^{PLE}(\mathbf{m}^{input}, \mathbf{m}^{mea}, \boldsymbol{\sigma}^{mea}, \boldsymbol{\theta}) = \sum_{i=1}^{k} SSR_{\mathbf{f}_i}^{LE}\left((\mathbf{m}_i^{input}, \mathbf{m}_i^{mea}, \boldsymbol{\sigma}_i^{mea}, \boldsymbol{\theta}\right), \qquad \left(S-1.3.9\right)$$

and the following constrained NLLSP must be solved to calculate the optimized $\mathbf{u}(\boldsymbol{\theta})$ fluxes, which are the variable parameters of the common metabolic model:

$$\begin{cases} \min\limits_{\boldsymbol{\theta} \in \Re^p} SSR_{\mathbf{f}}^{PLE}(\mathbf{m}^{input}, \mathbf{m}^{mea}, \boldsymbol{\sigma}^{mea}, \boldsymbol{\theta}) \\ \mathbf{u}(\boldsymbol{\theta}) \in \Re^n \end{cases}. \qquad \left(S-1.3.10\right)$$

If individual LEs introduce significant grouping factors or correlation effects in the flux estimation, then hierarchical regression models, i.e., multi-level models, must be used for flux calculations, with the corresponding application of the modified least-squared approaches [S-19]; however, these strategies were not implemented in the presented software.

One of the possible solutions of the NLLSP expressed by Eqs. $\left(S-1.3.8\right)$ and $\left(S-1.3.10\right)$ can be obtained using a gradient-based local optimization algorithm [S-20, S-21], i.e., a variation of Newton's method, which is explained in ***SF-1 Appendix 2***.

In OpenFLUX(2), NLLSP Eq. $\left(S-1.3.8\right)$ for SLEs is solved with the assistance of the MATLAB's FMINCON function, which is included in the Optimization Toolbox. Analogously, Eq. $\left(S-1.3.10\right)$ for PLEs is solved by

FMINCON in OpenFLUX2. By default, FMINCON implements a quasi-Newton sequential quadratic programming (SQP) method for constrained, nonlinear optimization using the Broyden-Fletcher-Goldfarb-Shanno (BFGS) formula, which is based on the linearization of the Jacobian matrix to approximate the Hessian matrix [S-21], instead of the approximation used in ***SF-1 Appendix 2***, Eq. $(A-2.9)$. Notably, from a computational point of view, a quasi-Newton method is preferred to Newton's approach because the former directly generates an approximation of the inverse Hessian matrix. However, the analytical form of the Hessian matrix is extremely important in the statistical analysis and in the calculation of the contribution matrix and of the *D*-factor for optimizing the experimental design (see (***SF-1.5.***)).

The applied SQP algorithm of the NLLSP solution can potentially converge to local minima of the objective function [S-20]. Thus, an iterative optimization procedure with a different set of initial $\boldsymbol{\theta} \in \Re^p$ values must be used to search for a global minimum [S-3, S-16]. In the present study, K reiterative trials, where $K \geq 100$, are typically used to obtain the set of optimized flux estimates, $\mathbf{u}_i(\widehat{\boldsymbol{\theta}}_i),\ i = 1,\ 2,\ \ldots,\ K$. Usually, each i-th optimization trial proceeds through step-wise iterative changes of free flux value, i.e., $\boldsymbol{\theta}_1, \boldsymbol{\theta}_2, \ldots, \boldsymbol{\theta}_g$, until reaching the minimum of the target objective function. In OpenFLUX(2), by default, the termination criterion is defined by the Karush-Kuhn-Tucker conditions, with a user-provided tolerance of the change in the objective function value (e.g., 1×10^{-4}) and the constraint violation tolerance (e.g., 1×10^{-3}). In contrast, termination can occur not only if the minimization criteria are satisfied but also, for example, if the amount of the iterations, g, which are valid during one trial, is exceeded. The result of such a trial cannot be

recognized as an intrinsic solution of the raised minimization problems. In OpenFLUX(2), the reasons for each termination are included in the Optimization report; therefore, only the successfully terminated trials can be considered. The further statistical evaluation of the resulting flux pseudo-populations of the $\mathbf{u}_i(\hat{\boldsymbol{\theta}}_i),\ i = 1,\ 2,\ \ldots,\ K$, can indicate the existence of feasible unique or multiple optimization solution(s).

SF-1.4. Statistical tests of the model adequacy

The computation of a set of NLLSP solutions, $\mathbf{u}_i(\hat{\boldsymbol{\theta}}_i)$, does not indicate that the user-made model is adequate. A goodness-of-fit analysis of the model is an essential related part of the flux determination using a regression procedure [S-4, S-22].

When the flux model adequately represents measurement data, then the arbitrary i-th residuals in the $\Xi(\boldsymbol{\theta}) \equiv 2 \cdot SSR_{\mathbf{f}}(\boldsymbol{\theta})$ function evaluated at the point of convergence (i.e., $\left(\dfrac{f(\mathbf{m}^{input}, \hat{\boldsymbol{\theta}})_i - (m_{MID}^{mea})_i}{(\sigma_{MID}^{mea})_i}\right),\ i = 1, 2, \ldots, w_{MID}$, and $\left(\dfrac{(\hat{\theta}_{eff}^{irr})_j - (q_{eff}^{mea})_j}{(\sigma_{eff}^{mea})_j}\right),\ j = 1, 2, \ldots, w_{eff}$), are expected to follow $\mathrm{N}(0,1)$ distribution.

Therefore, according to the χ^2-statistics, the null hypothesis, H_0, that the $\Xi(\hat{\boldsymbol{\theta}})$ value, which is a stochastic variable, ξ_{Ξ}, follows a χ^2–distribution at a point of convergence is considered along with the complex alternative, H_1, that the specified value has a different distribution. Moreover, the number of degrees of freedom for the χ^2-distribution must be equal to the number of independent measurements W ($W = w$ in the case of SLE, and $W = \sum_{i=1}^{k} w_i$ in the case of PLE,

respectively), minus the number of free fluxes, p, as estimated parameters, i.e., $(W-p)$. Thus, to test the goodness-of-fit, the following statistics are used:

$$\delta(\xi_\Xi)=\begin{cases} H_0, \text{if } \chi^2_{\alpha/2}(W-p)<\xi_\Xi<\chi^2_{1-\alpha/2}(W-p); \\ H_0, \text{if } \xi_\Xi \le \chi^2_{\alpha/2}(W-p); \text{however, the model might be overfitted;} \\ H_1, \text{if } \xi_\Xi \ge \chi^2_{1-\alpha/2}(W-p), \end{cases} \qquad (S-1.4.1)$$

where α is a significance level used as a certain threshold, e.g., 0.05 for a 95% confidence interval. If the determined ξ_Ξ value exceeds the maximum critical value, $\chi^2_{1-\alpha/2}(W-p)$, then the H_0 hypothesis must be rejected because the used flux model does not satisfy the experimental data at the tested significance level. Furthermore, if the acquired value ξ_Ξ is lower than the minimum critical value, $\chi^2_{\alpha/2}(W-p)$, then the weighted difference between the experimentally measured data and their model-based estimations is questionably small or the measurement's variances have been overestimated. Strictly speaking, the former has not been a cause to reject the hypothesis H_0 [S-23] but might serve as an indication of model overfitting using the specified set of parameters. Usually, the effect of overfitting occurs in complex models due to an imbalance between the number of measurements and the number of fitted parameters [S-24].

Initially, to analyze the set of NLLSP solutions obtained in K trials, $\mathbf{u}_i(\widehat{\boldsymbol{\theta}}_i)$, $i=1,2,\ldots,K$, it seems logical to confirm the normality of the distribution of the individual residuals, followed by a goodness-of-fit test of the $\Xi(\widehat{\boldsymbol{\theta}}_i)$ values. However, practically, it is convenient to initiate analysis due to the selection of all solutions by the χ^2-mediated criterion. In this case, the normality of the distribution can be analyzed only for the more interesting solutions (e.g., for one i-th solution corresponding to the minimal value of $\Xi(\widehat{\boldsymbol{\theta}}_i)$ or for all

solutions for which the H_0 hypothesis is accepted). This normality test can be extremely important, particularly when no obtained solutions satisfy the criterion of the H_0 hypothesis. Indeed, sometimes these residual values reveal systematic deviations, which may show how the experiment or the man-made model can be improved [S-7, S-20]. If several weighted residuals demonstrate a significant lack-of-fit in these statistical tests, then the results of the flux estimation process could be incorrect, most likely due to (*i*) the corresponding entries of $\mathbf{m}^{mea}$ and/or $\boldsymbol{\sigma}^{mea}$ being inadequately evaluated and (*ii*) the use of the wrong flux model structure. In the case of (*i*), a reasoned modification of the variance value or a deletion of the corresponding residuals from $\Xi(\hat{\boldsymbol{\theta}})$ could result in the acquisition of the proper values of $\mathbf{u}(\hat{\boldsymbol{\theta}})$ fluxes after the new attempt of the optimization procedure. In the case of (*ii*), particularly if a significant number of weighted residuals do not satisfy the tested criteria, then the re-evaluation of the flux model is necessary. Notably, the relative importance of residuals that have been chosen as lack-of-fit candidates for discard from $\Xi(\hat{\boldsymbol{\theta}})$ can also be quantitatively evaluated from the contribution matrix calculated by OpenFLUX2 (see ***SF-1.5.***). If alternative residuals can be chosen for deletion, then primarily the measurements that manifest lower relative importance can be used.

In OpenFLUX2, the proposed $\mathrm{N}(0,1)$ distribution of individual measurement variance-weighted residuals in the expression of $\Xi(\hat{\boldsymbol{\theta}})$ is analyzed with the assistance of MATLAB's Statistic Toolbox using the Kolmogorov-Smirnov test. Moreover, a plot of the absolute value of the individual variance-weighted residuals could be drawn. A simple visual inspection of the plot aids in identifying unpredictable high values for the individual residuals/group of

the individual residuals, followed by the re-evaluation of the corresponding measurements with their variances and/or by the re-evaluation of the man-made metabolic model.

SF-1.5. Local linearized statistical approximations

Several important statistical properties of the NLLSP solution could be determined using local linearized approximations of the nonlinear $\mathbf{f}(\mathbf{x}^{input},\boldsymbol{\theta})$ vector-function in the neighborhood of the convergence point [S-4, S-10, S-12]. The first of these properties is the flux identifiability analysis, i.e., whether the NLLSP has unique or multiple solutions that can be estimated from the available experimental data.

Assuming that the obtained estimations $\hat{\boldsymbol{\theta}}$ are close to the true free flux values, the nonlinear $\mathbf{f}(\mathbf{x}^{input},\boldsymbol{\theta})$ vector-function in Eq. $(S-1.3.4)$ can be linearly approximated using the Taylor's expansion in the neighborhood of $\hat{\boldsymbol{\theta}}$:

$$\mathbf{f}(\boldsymbol{\theta}) \cong \mathbf{f}(\hat{\boldsymbol{\theta}}) + \mathbf{J}_{\mathbf{f}}(\hat{\boldsymbol{\theta}}) \cdot (\boldsymbol{\theta} - \hat{\boldsymbol{\theta}}), \qquad (S-1.5.1)$$

where $\mathbf{J}_{\mathbf{f}}(\hat{\boldsymbol{\theta}})$ is the Jacobian matrix that equals $\partial \mathbf{f}(\boldsymbol{\theta})/\partial \boldsymbol{\theta}$ evaluated at $\boldsymbol{\theta} = \hat{\boldsymbol{\theta}}$. Let $\mathbf{y} = \mathbf{m}^{mea} - [\mathbf{f}(\hat{\boldsymbol{\theta}}) - \mathbf{J}_{\mathbf{f}}(\hat{\boldsymbol{\theta}}) \cdot \hat{\boldsymbol{\theta}}]$, then the initial nonlinear regression model in Eq. $(S-1.3.5)$ can be approximated using the following linear regression model:

$$\mathbf{y} = \mathbf{J}_{\mathbf{f}}(\hat{\boldsymbol{\theta}}) \cdot \boldsymbol{\theta} + (-\boldsymbol{\varepsilon}), \qquad (S-1.5.2)$$

where $\boldsymbol{\varepsilon}$ (see Eq. $(S-1.3.5)$) expectedly follows a multivariate normal distribution, i.e., $\boldsymbol{\varepsilon} \sim \mathrm{N}(\mathbf{0}, \Sigma_{\mathbf{x}})$, with $\Sigma_{\mathbf{x}}$ defined in Eq. $(S-1.3.3)$. Regression parameters, $\boldsymbol{\theta}$, of the linear model represented in Eq. $(S-1.5.2)$ can be estimated for the objective function $\Phi(\mathbf{y},\boldsymbol{\sigma}^{mea},\boldsymbol{\theta})$, which is assigned as follows:

$$\Phi(\mathbf{y},\boldsymbol{\sigma}^{mea},\boldsymbol{\theta}) = [\mathbf{y} - \mathbf{J}_{\mathbf{f}}(\hat{\boldsymbol{\theta}}) \cdot \boldsymbol{\theta}]^{\mathrm{T}} \cdot (\Sigma_{\mathbf{x}})^{-1} \cdot [\mathbf{y} - \mathbf{J}_{\mathbf{f}}(\hat{\boldsymbol{\theta}}) \cdot \boldsymbol{\theta}], \qquad (S-1.5.3)$$

as a solution of the following generalized least squares problem (GLSP):

$$\boldsymbol{\theta}_{GLSP} = \arg\min_{\boldsymbol{\theta}\in\Re^{p}} \left([\mathbf{y} - \mathbf{J_f}(\hat{\boldsymbol{\theta}})\cdot\boldsymbol{\theta}]^{\mathrm{T}} \cdot (\Sigma_{\mathbf{x}})^{-1} \cdot [\mathbf{y} - \mathbf{J_f}(\hat{\boldsymbol{\theta}})\cdot\boldsymbol{\theta}]\right) . \qquad (S-1.5.4)$$

The analytical solution of this GLSP can be found as a solution of the following equation:

$$\frac{\partial\Phi(\boldsymbol{\theta})}{\partial\boldsymbol{\theta}} = -2\cdot\mathbf{J_f}(\hat{\boldsymbol{\theta}})^{\mathrm{T}}\cdot(\Sigma_{\mathbf{x}})^{-1}\cdot(\mathbf{y} - \mathbf{J_f}(\hat{\boldsymbol{\theta}}))\cdot\boldsymbol{\theta}) = \mathbf{0} . \qquad (S-1.5.5)$$

Equation $(S-1.5.5)$ can be transformed into the following form:

$$\left(\mathbf{J_f}(\hat{\boldsymbol{\theta}})^{\mathrm{T}}\cdot(\Sigma_{\mathbf{x}})^{-1}\cdot\mathbf{J_f}(\hat{\boldsymbol{\theta}})\right)\cdot\boldsymbol{\theta} = \left(\mathbf{J_f}(\hat{\boldsymbol{\theta}})^{\mathrm{T}}\cdot(\Sigma_{\mathbf{x}})^{-1}\right)\cdot\mathbf{y} , \qquad (S-1.5.6)$$

which results in the linear estimation of the $\boldsymbol{\theta}$ parameters in the neighborhood of $\hat{\boldsymbol{\theta}}$ as a particular solution of Eq. $(S-1.5.6)$:

$$\boldsymbol{\theta}_{GLSP} = \left((\mathbf{J_f}(\hat{\boldsymbol{\theta}}))^{\mathrm{T}}\cdot(\Sigma_{\mathbf{x}})^{-1}\cdot\mathbf{J_f}(\hat{\boldsymbol{\theta}})\right)^{-1}\cdot\left((\mathbf{J_f}(\hat{\boldsymbol{\theta}}))^{\mathrm{T}}\cdot(\Sigma_{\mathbf{x}})^{-1}\right)\cdot\mathbf{y} , \qquad (S-1.5.7),$$

with the $\Sigma_{\hat{\boldsymbol{\theta}}}$ covariance matrix of the estimated free fluxes as follows:

$$\Sigma_{\hat{\boldsymbol{\theta}}} \equiv \mathrm{cov}\left(\hat{\boldsymbol{\theta}}\right) = \left((\mathbf{J_f}(\hat{\boldsymbol{\theta}}))^{\mathrm{T}}\cdot(\Sigma_{\mathbf{x}})^{-1}\cdot\mathbf{J_f}(\hat{\boldsymbol{\theta}})\right)^{-1} . \qquad (S-1.5.8)$$

Simultaneously, it is known (e.g., see [S-3, S-12]) that a general solution of a linear nonhomogeneous system (such as in Eq. $(S-1.5.6)$), $\mathbf{A}\cdot\mathbf{x} = \mathbf{b}$, can be expressed as the sum of a fixed particular solution, e.g., $(\mathbf{A}^{\mathrm{T}}\cdot\mathbf{A})^{-1}\cdot\mathbf{A}^{\mathrm{T}}\cdot\mathbf{b}$, and a general solution of the homogeneous system, $\mathbf{A}\cdot\mathbf{x} = \mathbf{0}$, i.e., an arbitrary element, $\mathbf{c}$, of the null space, $\mathbf{c}\in\mathrm{Null}(\mathbf{A})$, or $\mathbf{c} = \mathrm{Null}(\mathbf{A})\cdot\boldsymbol{\beta}$, where $\boldsymbol{\beta}$ is a vector containing arbitrary non-zero entries. Specifically, the solution set to Eq. $(S-1.5.6)$ is:

$$\boldsymbol{\theta} = \Sigma_{\hat{\boldsymbol{\theta}}}\cdot(\mathbf{J_f}(\hat{\boldsymbol{\theta}}))^{\mathrm{T}}\cdot(\Sigma_{\mathbf{x}})^{-1}\cdot\mathbf{y} + \mathrm{Null}[\mathbf{J_f}(\hat{\boldsymbol{\theta}})]\cdot\boldsymbol{\beta} , \qquad (S-1.5.9)$$

where $\mathrm{Null}[\mathbf{J_f}(\hat{\boldsymbol{\theta}})]$ is the null space of the $\mathbf{J_f}(\hat{\boldsymbol{\theta}})$ matrix. The following can be concluded from the provided free flux linearized approximations $(S-1.5.9)$: these $\hat{\theta}_i$ values of the $\hat{\boldsymbol{\theta}}$ flux vector can be determined uniquely from the

$\mathbf{y}(\mathbf{x}^{mea})$ vector, depending on the experimentally measured data for whose estimates the corresponding rows of the $\mathrm{Null}[\mathbf{J}_\mathbf{f}(\hat{\boldsymbol{\theta}})]$ matrix consist of zeros. In contrast, if the entry of $\mathrm{Null}[\mathbf{J}_\mathbf{f}(\hat{\boldsymbol{\theta}})]$ corresponding to $\hat{\theta}_i$ is non-zero, then infinitively many solutions for $\hat{\theta}_i$ at the obtained set of experimental data can be expected [S-12]. Thus NLLSP has a unique solution that justifies the available experimental data when the $\mathrm{Null}[\mathbf{J}_\mathbf{f}(\hat{\boldsymbol{\theta}})]$ matrix is empty.

Due to the limited precision of the floating-point numbers, in certain cases numerical estimation of the $\mathrm{Null}[\mathbf{J}_\mathbf{f}(\hat{\boldsymbol{\theta}})]$ can be inaccurate and result in unreliable conclusion of flux identifiability. To evaluate reliability of identifiability analysis in OpenFLUX2, condition number $\mathrm{cond}\left(\mathbf{J}_\mathbf{f}(\hat{\boldsymbol{\theta}})\right)$ is compared to precision $\mathrm{eps}\left(\mathbf{J}_\mathbf{f}(\hat{\boldsymbol{\theta}})\right)$ of the floating-point representation of the computed Jacobian values. As a general rule of thumb, if the condition number $\mathrm{cond}(A)=10^n$, then up to n digits of accuracy might be lost in computations. Thus, identifiability results can be considered reliable only when condition number $\mathrm{cond}\left(\mathbf{J}_\mathbf{f}(\hat{\boldsymbol{\theta}})\right)$ is within a certain upper bound. Taking into consideration the null space computation method used in MATLAB the following bound was chosen. If $\mathrm{cond}\left(\mathbf{J}_\mathbf{f}(\hat{\boldsymbol{\theta}})\right)<\frac{1}{\sqrt{\mathrm{eps}\left(\mathbf{J}_\mathbf{f}(\hat{\boldsymbol{\theta}})\right)}}$, condition number is considered acceptable and computations are treated as reliable. Otherwise, condition number is too large, Jacobian matrix is considered to be ill-conditioned and results of identifiability analysis are treated as unreliable. Corresponding messages about condition number status are displayed alongside with judgment about identifiability of fluxes, based on computation of $\mathrm{Null}[\mathbf{J}_\mathbf{f}(\hat{\boldsymbol{\theta}})]$.

As observed from the direct comparison of the $\Sigma_{\hat{\boldsymbol{\theta}}}$ covariance matrix determination presented in $(S-1.5.8)$, an approximation of the reverse Hessian matrix, $[\mathbf{H}_{SSR}(\hat{\boldsymbol{\theta}})]^{-1}$, which is introduced in ***Appendix 2***, Eq. $(A2.10)$, the following equation has to be true:

$$\Sigma_{\hat{\boldsymbol{\theta}}} \cong [\mathbf{H}_{SSR}(\hat{\boldsymbol{\theta}})]^{-1}. \qquad (S-1.5.10)$$

This relation is usually considered one of the convenient ways to compute the approximate covariance matrix of the estimated parameters [S-23].

From the covariance matrix properties and from the relation between the $\boldsymbol{\theta}$ and $\mathbf{u}$ fluxes expressed by Eq. $(S-1.1.6)$, the covariance matrix for the estimated optimal fluxes, $\Sigma_{\hat{\mathbf{u}}}$, can be presented as follows:

$$\Sigma_{\hat{\mathbf{u}}} = \mathrm{cov}(\mathbf{N}\cdot\hat{\boldsymbol{\theta}}) = \mathbf{N}\cdot\mathrm{cov}(\hat{\boldsymbol{\theta}})\cdot\mathbf{N}^{\mathrm{T}} = \mathbf{N}\cdot\Sigma_{\hat{\boldsymbol{\theta}}}\cdot\mathbf{N}^{\mathrm{T}}. \qquad (S-1.5.11)$$

According to Eqs. $(S-1.5.9)$ – $(S-1.5.11)$, $\Sigma_{\hat{\mathbf{u}}}$ can be computed as follows:

$$\begin{aligned}\Sigma_{\hat{\mathbf{u}}} &= \mathbf{N}\cdot(\mathbf{H}_{SSR})^{-1}\cdot\mathbf{N}^{\mathrm{T}} = \mathbf{N}\cdot(\mathbf{H}_{SSR})^{-1}\cdot\mathbf{H}_{SSR}\cdot(\mathbf{H}_{SSR})^{-1}\cdot\mathbf{N}^{\mathrm{T}} = \\ &= \mathbf{N}\cdot[\mathbf{H}_{SSR}(\hat{\boldsymbol{\theta}})]^{-1}\cdot[\mathbf{J}_{\mathbf{f}}(\hat{\boldsymbol{\theta}})]^{\mathrm{T}}\cdot(\Sigma_{\mathbf{x}})^{-1}\cdot\mathbf{J}_{\mathbf{f}}(\hat{\boldsymbol{\theta}})\cdot[\mathbf{H}_{SSR}(\hat{\boldsymbol{\theta}})]^{-1}\cdot\mathbf{N}^{\mathrm{T}}.\end{aligned} \qquad (S-1.5.12)$$

Thus, the form where the linearized approximation of the flux variances, $(\sigma^{\hat{\mathbf{u}}}_{lin})_i = \sqrt{(\Sigma_{\hat{\mathbf{u}}})_{i,i}}$, $i = 1, 2, ..., n$, is linked with the measurement variances, $(\sigma^{mea})_j = \sqrt{(\Sigma_{\mathbf{x}})_{j,j}}$, $j = 1, 2, ..., w$, has been obtained. A set of linearized statistics parameters can be computed using this equation. In particular, the linearized approximation of the flux confidence intervals at the confidence level of γ, $CI^{lin}_{\gamma}(u_i)$, can be estimated [S-4, S-17] through the $(\sigma^{\hat{\mathbf{u}}}_{lin})_i$ variances as follows:

$$CI^{lin}_{\gamma}(u_i) = [\left(\hat{u}_i - \sqrt{\chi^2_{\gamma}(1)}\cdot(\sigma^{\hat{\mathbf{u}}}_{lin})_i\right), \left(\hat{u}_i + \sqrt{\chi^2_{\gamma}(1)}\cdot(\sigma^{\hat{\mathbf{u}}}_{lin})_i\right)]. \qquad (S-1.5.13)$$

These intervals must be additionally constrained according to Eq. $(S-1.2.6)$.

Flux parameters that are characterized by the local linearized statistics could be used for optimizing the experimental design according to the method supposed by Möllney et al. [S-10]. In this study, a comparison of different experimental designs was provided by the *D*-criterion [S-26], which means evaluation of the squared volumes of the confidence ellipsoids with the size of the axis equaled to $CI_{\gamma}^{lin}(u_i)$ for a given confidence level. According to [S-10], the *D*-factor, proportional (up to a constant that depends on confidence level γ) to the squared volume of the *p*-dimensional ellipsoid for the variances of the optimized free fluxes, is given by the following equation:

$$D(\mathbf{m}^{input},\mathbf{m}^{mea},\boldsymbol{\sigma}^{mea},\hat{\boldsymbol{\theta}}) = \det\,\mathrm{cov}\,(\mathbf{m}^{input},\mathbf{m}^{mea},\boldsymbol{\sigma}^{mea},\hat{\boldsymbol{\theta}}) \equiv \det\,\Sigma_{\hat{\boldsymbol{\theta}}} \cong \det[\mathbf{H}_{SSR}(\hat{\boldsymbol{\theta}})]^{-1}, \quad (S-1.5.14)$$

where the last equality, which is based on Eq. $(S-1.5.10)$, is used for the *D*-factor computation in OpenFLUX2. Several properties of Hessian matrix (like positive definite, condition number and *etc.*) are additionally controlled to provide reliability of *D*-factor calculation. The *D*-factor must be minimized with respect to the space of all feasible input tracers, $\mathbf{m}^{input}$, if the minimum ellipsoid volume for estimated free flux variances is the aim of the experimental design optimization as follows:

$$D_{opt}(\mathbf{m}^{input},\mathbf{m}^{mea},\boldsymbol{\sigma}^{mea},\hat{\boldsymbol{\theta}}) = \min_{\mathbf{m}^{input}-feasible} \det[\mathbf{H}_{SSR}(\hat{\boldsymbol{\theta}})]^{-1}. \quad (S-1.5.15)$$

Simultaneously, if the minimization of the targeted flux variance equaled to $\sqrt{(\Sigma_{\hat{\boldsymbol{\theta}}})_{ii}}$ is desired, then it is necessary to solve the following problem:

$$\min_{\mathbf{m}^{input}-feasible} \left(\sigma_{lin}^{\hat{\boldsymbol{\theta}}}\right)_i = \min_{\mathbf{m}^{input}-feasible} \sqrt{\left([\mathbf{H}_{SSR}(\boldsymbol{\theta}_{true})]^{-1}\right)_{ii}}\,. \quad (S-1.5.16)$$

Another field where the flux parameters evaluated by local linearized statistics play an important role in ^{13}C-MFA is in computing and applying the so called “contribution matrix”, which, in fact, is the squared value of the

Pearson's linear correlation coefficients between fluxes and measurements calculated based on a linear approximation of the nonlinear vector-function $\mathbf{f}(\mathbf{x}^{input},\boldsymbol{\theta})$ using Taylor's expansion. According to [S-4], Eq. $(S-1.5.12)$ is used to construct the contribution matrix, $\mathbf{CM}$, $\dim(\mathbf{CM})=(n\times w)$, with the $(\mathbf{CM})_{i,j}$ elements manifesting the relative importance of the variance of the j-th measurement to the approximated variance of the i-th flux as follows:

$$(\mathbf{CM})_{i,j}=\frac{\left(\mathbf{N}\cdot[\mathbf{H}_{SSR}(\hat{\boldsymbol{\theta}})]^{-1}\cdot[\mathbf{J}_{\mathbf{f}}(\hat{\boldsymbol{\theta}})]^{\mathrm{T}}\right)_{i,j}^{2}}{(\Sigma_{\hat{\mathbf{u}}})_{i,i}\cdot(\Sigma_{\mathbf{x}})_{j,j}}=\frac{\left(\mathbf{N}\cdot[\mathbf{H}_{SSR}(\hat{\boldsymbol{\theta}})]^{-1}\cdot[\mathbf{J}_{\mathbf{f}}(\hat{\boldsymbol{\theta}})]^{\mathrm{T}}\right)_{i,j}^{2}}{(\sigma_{lin}^{\hat{\mathbf{u}}})_{i}\cdot(\sigma^{mea})_{j}}, \qquad (S-1.5.17)$$

where the symmetry of the Hessian matrix, i.e., $\mathbf{H}_{SSR}=(\mathbf{H}_{SSR})^{\mathrm{T}}$ (and thus, $(\mathbf{H}_{SSR})^{-1}=\left((\mathbf{H}_{SSR})^{-1}\right)^{\mathrm{T}}$) is used in the equation derivation process. Thus, the $(\mathbf{CM})_{i,j}$ elements with large values indicate that the j-th measurement is important for evaluating the i-th flux. Furthermore, the sum of elements in an arbitrary row of the contribution matrix equals one (see ***SF-1 Appendix 3***). In turn, the sum of elements of an arbitrary j-th column of the $\mathbf{CM}$ matrix indicates the relative importance of the corresponding j-th measurement. Consequently, the large value of the sum column elements indicates the measurement that significantly contributes to the solution of the flux estimation problem. Usually [S-4], the tracer experiment is considered inadequately designed when a significant number of estimated fluxes depends on a small number of measurements. Computing the contribution matrix according to Eq. $(S-1.5.17)$ was incorporated into OpenFLUX2.

SF-1.6. Nonlinear search of flux confidence intervals

The nonlinear-based approach developed by Antoniewicz et al. [S-4] was implemented in OpenFLUX(2) to search for an estimation of the flux confidence

intervals, $CI_{\gamma}^{n-lin}(u_i)$, which is more accurate than the linear approximation, $CI_{\gamma}^{lin}(u_i)$. This approach is based on the assumption that provided measurement's errors data for the flux model used in SLEs (or in PLEs (Exp. (S-1.4.1))) follows normal distribution and correctly weights model's residuals. Therefore, at a convergence point, $\mathbf{u}(\hat{\boldsymbol{\theta}}) = (u_1(\hat{\boldsymbol{\theta}}),\ u_2(\hat{\boldsymbol{\theta}}),\ ...,\ u_n(\hat{\boldsymbol{\theta}}))^{\mathrm{T}}$, the value $\xi_{\Xi} = \min_{\boldsymbol{\theta}\in\Re^p}(\Xi(\boldsymbol{\theta}))$ could be treated as a random variable, which follows a χ^2-distribution with $(W-p)$ degrees of freedom. For the $u_i(\boldsymbol{\theta})$ flux, let $\psi_i(v) = \min_{\boldsymbol{\theta}\in\Re^p}(\Xi(\boldsymbol{\theta};\ u_i(\boldsymbol{\theta}) = v))$ denote the optimal value of the $\Xi(\boldsymbol{\theta})$ function when the $u_i(\boldsymbol{\theta})$ flux is fixed at the point, where $u_i(\boldsymbol{\theta}) = v$. Assuming the absence of mathematical singularity for the $\Xi(\boldsymbol{\theta})$ function around a point of convergence, the value of the function $\psi_i(v)$ for fixed v can be considered as a random variable that follows χ^2-distribution with $(W-p-1)$ degrees of freedom. Due to the additivity of the χ^2-distribution, the non-negative difference $(\psi_i(v) - \xi_{\Xi})$ follows a χ^2-distribution with one degree of freedom. According to Antoniewicz et al. [S-4], an approximate $\gamma\times100\%$ confidence interval for u_i, $CI_{\gamma}^{n-lin}(u_i)$, is given by the flux values for which the following inequality is true:

$$\psi_{\mathrm{i}}(v) < \xi_{\Xi} + \chi_{\gamma}^2(1). \qquad (S-1.6.1)$$

In OpenFLUX(2), the lower and upper bounds, $LB_{\gamma}^{n-lin}(u_i)$ and $UB_{\gamma}^{n-lin}(u_i)$ of the corresponding confidence interval $CI_{\gamma}^{n-lin}(u_i)$ are obtained as a solution by MATLAB's FMINCON of the following non-linear optimization problems:

$$\begin{cases} LB_{\gamma}^{n-lin}(u_i) = \min_{\boldsymbol{\theta}} u_i(\boldsymbol{\theta}) \\ \Xi(\boldsymbol{\theta}) - \xi_{\Xi} < \chi_{\gamma}^2(1) \\ \chi_{\gamma}^2(1) - (\Xi(\boldsymbol{\theta}) - \xi_{\Xi}) < \beta \\ u_i(\boldsymbol{\theta}) \le u_i(\widehat{\boldsymbol{\theta}}) \\ \boldsymbol{\theta} \in \Re^p \end{cases}, \qquad \begin{cases} UB_{\gamma}^{n-lin}(u_i) = \max_{\boldsymbol{\theta}} u_i(\boldsymbol{\theta}), \\ \Xi(\boldsymbol{\theta}) - \xi_{\Xi} < \chi_{\gamma}^2(1) \\ \chi_{\gamma}^2(1) - (\Xi(\boldsymbol{\theta}) - \xi_{\Xi}) < \beta \\ u_i(\boldsymbol{\theta}) \ge u_i(\widehat{\boldsymbol{\theta}}) \\ \boldsymbol{\theta} \in \Re^p \end{cases}, \qquad (S-1.6.2)$$

where β – a user-provided constraint tolerance, and the optimization procedure is started from the convergence point, $u_i(\widehat{\boldsymbol{\theta}})$, for each $CI_{\gamma}^{n-lin}(u_i)$ bound.

Thus in OpenFLUX(2), the $CI_{\gamma}^{n-lin}(u_i)$ intervals determined at the confidence level of γ are presented as follows:

$$CI_{\gamma}^{n-lin}(u_i) = [LB_{\gamma}^{n-lin}(u_i),\ UB_{\gamma}^{n-lin}(u_i)]\,. \qquad (S-1.6.3)$$

It should be noted, that, despite the fact that solution to the optimization problems $(S-1.6.2)$ gives the correct minimal and maximal flux values, which satisfy inequality $(S-1.6.1)$, computational approach proposed in OpenFLUX(2) is different from the algorithm proposed by Antoniewicz et al. [S-4]. Nevertheless, these computational approaches are equivalent in case, when $\psi_i(v)$ is a strictly monotone increasing function.

Since the lower and upper bounds of CI_{γ}^{n-lin} are obtained as a result of fitting procedure, it is important to perform detailed analysis of the optimization results. Original implementation of the non-linear search algorithm in OpenFLUX detects simple cases, when acquired value for the CI_{γ}^{n-lin} bounds may be unreliable, but does not provide tools for comprehensive analysis of the quality of acquired approximation of the respective CI_{γ}^{n-lin}, and control of error propagation in optimization procedure.

It was detected, that in some cases significant bias was introduced in CI_{γ}^{n-lin} estimation, which made it impossible to compare connfidence intervals

for different γ (e.g., for certain models it could be observed, that estimated $CI_{0.95}^{n-lin}$ were significantly narrower than $CI_{0.68}^{n-lin}$). Several examples of these "mistaken" evaluations usually detected for the minor part of the tested fluxes, were described in the item **2.4** of **Results**.

One of the reasons for poor CI_{γ}^{n-lin} estimation results was the fact that original OpenFLUX non-linear confidence intervals estimation procedure did not take into consideration the first-order optimality values calculated during FMINCON execution. These values represent fulfillment of necessary optimality conditions (Karush-Kuhn-Tucker conditions see, e.g., [S-21]) for constraint optimization problems. Thus, for certain metabolic models and function's termination tolerances. it led to termination of the optimization procedure before it reached the lower or upper bounds of the corresponding $CI_{\gamma}^{n-lin}(u_i)$. Original OpenFLUX software could incorrectly consider that optimization had converged in such cases. Thus, it is necessary to perform additional evaluation of precision of CI_{γ}^{n-lin} obtained by non-linear search algorithm.

Furthermore, adjustment of optimization parameters (e.g., function termination tolerance and constraint tolerance) can lead to significant improvement of first-order optimality values and consequently precision of CI_{γ}^{n-lin} determined by non-linear confidence estimation algorithm implemented in OpenFLUX.

Despite the fact, that current version of OpenFLUX2 still utilizes original implementation of the CI_{γ}^{n-lin} estimation algorithms, several modifications targeted to improve precision of computations, are now in the process of development.

One of the main advantages of the CI_{γ}^{n-lin} evaluation is its computation speed that is significantly higher than for more precise, but rather slow Monte Carlo-based approach (see, the item **2.4** of **Results**, and ***SF-1.7*** below). It means, that even the current, not perfect non-linear search algorithm implemented in OpenFLUX(2) could be efficiently used for the fast preliminary evaluation of the flux confidence intervals according to Eq. $(S-1.6.3)$. For the most part of fluxes, these evaluations are close to the precise parameters that could be determined later by Monte Carlo-based approach.

Using the computed $CI_{\gamma}^{n-lin}(u_i)$ parameters, it is possible to assign the value of the *i*-th “flux standard deviation” or “flux precision” [S-18], $\sigma_i^{n-lin}(\gamma)$, instead the linear approximations σ_i^{lin} (see, ***SF-1.5***), by the following way:

$$\sigma_{\gamma}^{n-lin}(u_i) = \frac{UB_{\gamma}^{n-lin}(u_i) - LB_{\gamma}^{n-lin}(u_i)}{2 \cdot \sqrt{\chi_{\gamma}^2(1)}}, \qquad (S-1.6.4)$$

formally comparing Eqs. $(S-1.5.13)$ and $(S-1.6.3)$. Eq. $(S-1.6.4)$ can be simplified [S-18] for the $\gamma = 0.95$ (95% confidence interval) to the following equation:

$$\sigma_{0.95}^{n-lin}(u_i) = \frac{1}{2 \cdot \sqrt{3.841}} \cdot \left(UB_{0.95}^{n-lin}(u_i) - LB_{0.95}^{n-lin}(u_i)\right) \approx \frac{1}{4} \cdot \left(UB_{0.95}^{n-lin}(u_i) - LB_{0.95}^{n-lin}(u_i)\right). \qquad (S-1.6.5)$$

Namely the value of $\sigma_{0.95}^{n-lin}(u_i)$ have been earlier used for evaluation the achieved flux resolution [S-18, 27]. Our proposal is to introduce the function $\eta_{\gamma}^{n-lin}\left(u_i(\hat{\boldsymbol{\theta}}), \beta\right)$, which is assigned as “the normalized flux precision at a determined confidence level of γ” or “normalized flux precision”, which must be computed in the following manner:

$$\eta_{\gamma}^{n-lin}\left(u_i(\widehat{\boldsymbol{\theta}}),\beta\right)=\begin{cases}1-\dfrac{CI_{\gamma}^{n-lin}(u_i)}{u_i\left(\widehat{\boldsymbol{\theta}}\right)+\beta\cdot\max\mathbf{V}_{eff}^{mea}}\cdot,\ \text{when}\ CI_{\gamma}^{n-lin}(u_i)\le u_i\left(\widehat{\boldsymbol{\theta}}\right)+\beta\cdot\max\mathbf{V}_{eff}^{mea}\\ 0,\ \text{when}\ CI_{\gamma}^{n-lin}(u_i)>u_i\left(\widehat{\boldsymbol{\theta}}\right)+\beta\cdot\max\mathbf{V}_{eff}^{mea}\end{cases},\qquad (S-1.6.6)$$

where β is a scaling parameter and $\max\mathbf{V}_{eff}^{mea}$ is the maximal measured efflux, which, usually, corresponds to input carbon flux. It should be noted that the $u_i\left(\widehat{\boldsymbol{\theta}}\right)$ flux value is the best available estimation computed from the SLE- or PLE-based ^{13}C-MFA, which approximates the unknown true flux value. In the present study, the known values of the true fluxes were used for estimation of flux precision efficiencies achieved due to analysis of different LEs performed *in silico*. According to Eq. $(S-1.6.6)$, in the case of rather small or zero values of flux parameters their estimation of flux precision is evaluated relative to the $\max\mathbf{V}_{eff}^{mea}$, as some characteristic value. A variable scaling parameter β provides flexibility in determination of what is the “slow” fluxes, depending on the case context. The scaling parameter β had been set to 0.1 at the present study. According to its definition, the $\eta_{\gamma}^{n-lin}\left(u_i(\widehat{\boldsymbol{\theta}}),\beta\right)$ function at each fixed β parameter is close to “1” for precisely estimated flux $u_i\left(\widehat{\boldsymbol{\theta}}\right)$ (with narrow confidence interval) and is close to “0” for poorly determined flux $u_i\left(\widehat{\boldsymbol{\theta}}\right)$.

Analogously, the normalized flux precision functions can be defined using the flux confidence intervals determined using the linearized approximation of the flux variances (see Eq. $(S-1.5.13)$) or the Monte Carlo-based approach (see ***SF-1.7***). The corresponding superscript, lin, $n-lin$, $MC-1$ or $MC-2$ in the definition of the flux precision function, e.g., $\eta_{\gamma}^{MC-1}\left(u_i(\widehat{\boldsymbol{\theta}}),\beta\right)$ or $\eta_{\gamma}^{lin}\left(u_i(\widehat{\boldsymbol{\theta}}),\beta\right)$, directly indicates the method used for computing the flux confidence intervals.

SF-1.7. Determination of flux confidence intervals using the Monte Carlo-based approach

Usually in the present study, the flux confidence intervals were determined using the Monte Carlo-based approach included previously in OpenFLUX [S-6], but substantively modified in the final design of OpenFLUX2 to allow fine-tuning of procedures parameters and add convergence control.

According to [S-16], the Monte Carlo approach of flux confidence interval search was based on discrete approximation of optimized flux parameter distributions obtained in L multi-trials when the measurement's data for each trial were artificially generated by applying normally distributed random errors to initially obtained experimental measurements data. The number of trials, L, is one of the important parameters of Monte Carlo-based procedure, and it has to be optimally chosen (L=L_{MAX}) for flux confidence intervals, CI_{γ}^{MC} , precise determination in a reasonable computation time (see, below). The data variations were performed by corrupting the MIDs and the effluxes measured with the corresponding deviations, $\boldsymbol{\sigma}_{MID}^{mea}$ and $\boldsymbol{\sigma}_{eff}^{mea}$. Random errors, which followed $\mathrm{N}(\mathbf{0}, \Sigma_{\mathbf{x}})$ distribution (with the relation in accordance to Eq. $(S-1.3.3)$ between $\Sigma_{\mathbf{x}}$, $\boldsymbol{\sigma}_{MID}^{mea}$, and $\boldsymbol{\sigma}_{eff}^{mea}$) were generated using the statistics Toolbox of MATLAB. Then, the generated L data sets, $\mathbf{m}_{j}^{mea}$, $j = 1, 2, ..., L$, were used as new "experimental" data in corresponding L constrained NLLSPs (as in case of Eq. $(S-1.3.8)$) to generate the following set of optimized flux estimations using the fitting-based quasi-Newton SQP method (see ***SF-1.3.***):

$$\mathbf{U}(\hat{\Theta}_L) = (\mathbf{u}(\hat{\boldsymbol{\theta}}_1), \mathbf{u}(\hat{\boldsymbol{\theta}}_2), ..., \mathbf{u}(\hat{\boldsymbol{\theta}}_j), ..., \mathbf{u}(\hat{\boldsymbol{\theta}}_L)), \qquad (S-1.7.1)$$

where $\widehat{\Theta}_L = \left(\widehat{\boldsymbol{\theta}}_1, \widehat{\boldsymbol{\theta}}_2, ..., \widehat{\boldsymbol{\theta}}_L\right)$ and $\mathbf{u}\left(\widehat{\boldsymbol{\theta}}_j\right) = \left(u_1(\widehat{\boldsymbol{\theta}}_j), u_2(\widehat{\boldsymbol{\theta}}_j), ..., u_n(\widehat{\boldsymbol{\theta}}_j)\right)^{\mathrm{T}}$, $j = 1, 2, ..., L$. Two different methods were implemented in OpenFLUX(2) to decrease computational time of the optimization process of each trial in Monte Carlo-based search of CI_{γ}^{MC} in comparison with the time needed to acquire optimized flux parameters as a result of the search for the solution of the initial constrained NLLSP.

The first method consists of a single run of the optimization procedure for each trial, that uses optimized free fluxes $\widehat{\boldsymbol{\theta}}$, which were obtained for the uncorrupted measurement data, as a starting point. This method, “single run per trial”, results in fast performance but tends to determine the local minimum instead of the global minimum.

The second method incorporates a simple technique, which attempts to determine the global minimum of the $\Xi(\boldsymbol{\theta})$ function. Instead of a single run in each trial, this technique consists of sequential multiple optimization runs from random starting points $\boldsymbol{\theta} \in \Re^p$, that additionally satisfy the inequality $(S-1.2.1)$. As described previously (see ***SF-1.3.***), each run proceeds through step-wise iterative changes of free flux values until reaching the minimum of the target objective function with the tunable termination criterion, TT – termination tolerance, which is by default equal to 1×10^{-4} in OpenFLUX2 (it is a tunable parameter in Monte Carlo search of flux confidence intervals). In each trial, the set of the best found local minimum is stored during optimization. If the new run results in a better optimized value $\xi'_{\Xi} = \Xi(\boldsymbol{\theta}')$ of $\Xi(\boldsymbol{\theta})$, then the set is emptied, and the new value ξ'_{Ξ} and the corresponding set of flux values $\mathbf{u}(\boldsymbol{\theta}')$ is stored. If the value ξ'_{Ξ} of the current run is close to the currently stored best

value $\hat{\xi}_{\Xi} = \Xi(\hat{\boldsymbol{\theta}})$, then this run solution is added to the set. Formally, the distance between the corresponding values (proximity) is defined by the following expression:

$$\rho\left(\xi'_{\Xi},\, \hat{\xi}_{\Xi}\right) = \left|\frac{\hat{\xi}_{\Xi} - \xi'_{\Xi}}{\hat{\xi}_{\Xi}}\right|, \qquad (S-1.7.2)$$

Thus, the current optimized value ξ'_{Ξ} is added to the set when $\rho\left(\xi'_{\Xi},\, \hat{\xi}_{\Xi}\right) \le \varepsilon$, where ε is a predefined proximity threshold (it is a tunable parameter in OpenFLUX2). The value $\varepsilon = 0.05$ has been initially used as a default in OpenFLUX(2), but it is highly recommended to decrease the default value up to $\varepsilon = 1\times 10^{-4}$ or below (see, **Figure SF-2.4**), to improve precision of CI_{γ}^{MC} determination with the help of OpenFLUX2. Notably, runs that encounter any problems during optimization step (e.g., the optimization procedure stops before reaching a prescribed convergence threshold) are completely ignored. Accordingly, the reiterative run solutions are accumulated. The procedure terminates when the size of the accumulated set exceeds a certain threshold value N_{AS} (it is a tunable parameter, and by default N_{AS}=3 in OpenFLUX2). Additionally, it is possible to constrain the number of runs in one trial. In this case, optimization procedure in each trial will terminate after performing the prescribed number of runs K_{NR} (it is a tunable parameter, and by default K_{NR}=50 in OpenFLUX2), when the set of accumulated run solutions is not filled completely, and the parameters corresponding to the best stored run would be considered as optimized for this trial. This mandatory termination leads to faster performance but can potentially demonstrate poorer results in comparison with the case when the number of run per trial is not restricted.

Certainly, optimization procedure that was performed according to the second strategy, “multiple runs per trial”, results in slower performance, but increases the chances of finding the global minimum for each out of L trials and, therefore, better estimates the flux confidence intervals. Namely, this strategy is used as a default approach for Monte Carlo procedure in OpenFLUX2, but the alternative, “one run per trial”, method could be used according to the user’s choice. Furthermore, in OpenFLUX2, it is possible to fine-tune the TT, ε, N_{AS} and K_{NR} parameters of the above-described procedure to govern solution accuracy and computation time of each trial. For example, in case of unrestricted number of runs per trial, values for the proximity threshold ε and the size of the accumulated run solutions set N_{AS} can be decreased or increased in comparison with the default values, correspondingly, to improve precision of the CI_{γ}^{MC} determination. Usually, these improvements resulted in narrowing of the CI_{γ}^{MC} width, that, in turn, resulted in decrease of the upper bound, and in increase of the lower bound of the target flux confidence interval. Computed CI_{γ}^{MC} ’s estimations for one of the fluxes as a function of different values of tunable parameters, which determine the accuracy of the constrained NLLSP solution in each trial of Monte Carlo-based approach, are presented in the **Figure SF-2.4**.

Therefore, one of the above-described strategies is used iteratively to generate a set of independently obtained flux distributions of size L (Eq. $(S-1.7.1)$). To determine the flux confidence intervals on the basis of these distributions, two different approaches are implemented in OpenFLUX2. According to the first approach, “discarding” strategy, for each $i, i=1,...,n,$ the set $\mathbf{U}_i(\widehat{\Theta}_L)=(u_i(\widehat{\boldsymbol{\theta}}_1), u_i(\widehat{\boldsymbol{\theta}}_2),..., u_i(\widehat{\boldsymbol{\theta}}_j),..., u_i(\widehat{\boldsymbol{\theta}}_L))$ of flux estimated values is first sorted

in ascending order. Then, the $\gamma \times 100\%$ (e.g., 95%, 80 or 68%) confidence interval on the basis of L trials, $CI_{\gamma}^{MC-1}\left(\mathbf{U}_i(\hat{\Theta}_L)\right)$, is obtained by discarding the top $\frac{1-\gamma}{2} \times 100\%$ (e.g., 2.5%, 10 or 16%) and the bottom $\frac{1-\gamma}{2} \times 100\%$ of the flux estimates $u_i\left(\hat{\boldsymbol{\theta}}_j\right)$, $j = 1,..., L$, which results (see, for example, [S-4]) in determination of upper and lower bounds $UB_{\gamma}^{MC-1}(u_i; L)$ and $LB_{\gamma}^{MC-1}(u_i; L)$ respectively, for each $u_i\left(\hat{\Theta}_L\right)$, $i = 1, 2,..., n$ at a confidence level of γ (see, **Figure 4** in the main text for clarity).

Alternatively [S-6, S-16, S-17], the “mean-varianced” strategy could be used, where i-th flux mean, $\mu_i^{MC-2}\left(\hat{\Theta}_L\right) = \frac{1}{L} \cdot \sum_{j=1}^{L} u_i\left(\hat{\boldsymbol{\theta}}_j\right)$, and the unbiased estimator of the variance, $\sigma_i^{MC-2}\left(\hat{\Theta}_L\right) = \sqrt{\frac{1}{(L-1)} \cdot \sum_{j=1}^{L} \left(u_i(\hat{\boldsymbol{\theta}}_j) - \mu_i^{MC-2}(\hat{\Theta}_L)\right)^2}$, are calculated to generate the confidence intervals at the confidence level of γ on the basis of L trials, $CI_{\gamma}^{MC-2}\left(\mathbf{U}_i(\hat{\Theta}_L)\right)$, which, finally, must be additionally constrained by the range $[\mathbf{lb}_i, \mathbf{ub}_i]$ of the corresponding feasible values for the i-th flux as follows:

$$CI_{\gamma}^{MC-2}\left(\mathbf{U}_i(\hat{\Theta}_L)\right) = \left[\max\left\{\mathbf{lb}_j, \mu_i^{MC-2}(\hat{\Theta}_L) - \Delta_{\gamma}(\hat{\Theta}_L)\right\}, \min\left\{\mu_i^{MC-2}(\hat{\Theta}_L) + \Delta_{\gamma}(\hat{\Theta}_L), \mathbf{ub}_j\right\}\right], \quad (S-1.7.3)$$

where $\Delta_{\gamma}(\hat{\Theta}_L) = \sigma_i^{MC-2}(\hat{\Theta}_L) \cdot \sqrt{\chi_{\gamma}^2(1)}$.

Finally, the Monte Carlo-based confidence intervals computed by “discarding”, $CI_{\gamma}^{MC-1}(u_i)$, or “mean-varianced”, $CI_{\gamma}^{MC-2}(u_i)$, strategies have to be determined as:

$$CI_{\gamma}^{MC-1}(u_j) = \lim_{L\to\infty} CI_{\gamma}^{MC-1}\left(\mathbf{U}_i\left(\hat{\Theta}_L\right)\right), \quad CI_{\gamma}^{MC-2}(u_j) = \lim_{L\to\infty} CI_{\gamma}^{MC-2}\left(\mathbf{U}_i\left(\hat{\Theta}_L\right)\right) \quad (S-1.7.4)$$

i.e., the estimated values of upper and lower bounds of $CI_{\gamma}^{MC}(u_i)$ have to converge in case of significant increase of the total number of trials. The special

ONLINE procedure has been implemented in OpenFLUX2 to control the number of optimization trials that is performed during estimation of target flux $CI_{\gamma}^{MC}(u_i)$ bound.

The designed control procedure is based on the assumption that increase in the number of trials results in improvement of approximation precision. That, in turn, has to result in convergence of $CI_{\gamma}^{MC}(u_i)$ bounds. In OpenFLUX2, the method of "sliding control" has been applied for evaluation of this convergence. Let, M be a preset number that characterized a sliding control "window" size. First, for each set $\mathbf{U}(\hat{\Theta}_{L-k})$ (see, Eq. $(S-1.7.1)$), $k = 0, 1, ..., (M-1)$, and i-th flux, $i = 1, 2, ..., n$, at confidence level of γ the bounds of $CI_{\gamma}^{MC}(u_i)$ are estimated, using, for example, the "discarding" strategy:

$$CI_{\gamma}^{MC-1}(\mathbf{U}_i(\hat{\Theta}_{L-k})) = \left[LB_{\gamma}^{MC-1}(\mathbf{U}_i(\hat{\Theta}_{L-k})),\ UB_{\gamma}^{MC-1}(\mathbf{U}_i(\hat{\Theta}_{L-k}))\right]. \qquad (S-1.7.5)$$

Then an absolute spread in the estimated lower (and upper) bounds of flux confidence intervals can be finally calculated as:

$$\Delta_{LB}^{MC-1}(\gamma, i, \hat{\Theta}_L, M) = \left| \max_{0\le k\le M-1} LB_{\gamma}^{MC-1}(\mathbf{U}_i(\hat{\Theta}_{L-k})) - \min_{0\le k\le M-1} LB_{\gamma}^{MC-1}(\mathbf{U}_i(\hat{\Theta}_{L-k})) \right|, \qquad (S-1.7.6)$$

$$\Delta_{UB}^{MC-1}(\gamma, i, \hat{\Theta}_L, M) = \left| \max_{0\le k\le M-1} UB_{\gamma}^{MC-1}(\mathbf{U}_i(\hat{\Theta}_{L-k})) - \min_{0\le k\le M-1} UB_{\gamma}^{MC-1}(\mathbf{U}_i(\hat{\Theta}_{L-k})) \right|.$$

Consequently, values of a relative spread for all estimated bounds can be determined as:

$$\delta_{LB}^{MC-1}(\gamma, i, \hat{\Theta}_L, M) = \frac{2\times\Delta_{LB}^{MC-1}(\gamma, i, \hat{\Theta}_L, M)}{\left| \max_{0\le k\le M-1} LB_{\gamma}^{MC-1}(\mathbf{U}_i(\hat{\Theta}_{L-k})) + \min_{0\le k\le M-1} LB_{\gamma}^{MC-1}(\mathbf{U}_i(\hat{\Theta}_{L-k})) \right|}, \qquad (S-1.7.7)$$

$$\delta_{UB}^{MC-1}(\gamma, i, \hat{\Theta}_L, M) = \frac{2\times\Delta_{UB}^{MC-1}(\gamma, i, \hat{\Theta}_L, M)}{\left| \max_{0\le k\le M-1} UB_{\gamma}^{MC-1}(\mathbf{U}_i(\hat{\Theta}_{L-k})) + \min_{0\le k\le M-1} UB_{\gamma}^{MC-1}(\mathbf{U}_i(\hat{\Theta}_{L-k})) \right|}.$$

If the obtained relative spread does not exceed the predetermined threshold for the targeted flux, it could be considered that the corresponding bound of $CI_{\gamma}^{MC}(u_i)$ converged (see, **Figure 5** for clarity).

OpenFLUX2 allows fine tuning of the Monte Carlo-based procedure in such a fashion, that the convergence for all $CI_{\gamma}^{MC}(u_i)$ bounds results in termination of calculations, even if the predetermined maximal number of trials, L_{MAX}, has not been achieved yet. This option can help to optimize the number of performed trials, and, in several cases, significantly decrease computation time for Monte Carlo-based approach.

OpenFLUX2 allows to fine-tune sliding control “window” to allow the user to balance between computation time and precision of $CI_{\gamma}^{MC}(u_i)$ determination. Indeed, a user can tune different parameters of “window” (e.g., size, deepness, frequency), modify the value of predetermined threshold for CI_{γ}^{MC} bounds. To save computation time, the user can preset the frequency of a sliding control tests (e.g., once per 40 performed trials). Moreover, OpenFLUX2 allows to analyze using the described sliding control “window” strategy estimations of CI_{γ}^{MC}, which were earlier obtained by Monte Carlo-based approach. The auxiliary visualization tools were implemented in OpenFLUX2 that allow plotting of optimized flux distributions, as well as displaying convergence plots of CI_{γ}^{MC} bounds as a function of the number of performed trials (see, **Figure 5, Figure SF-2.4**, as examples).

That is to say, an automatic convergence control is implemented at the stage of CI_{γ}^{MC} bound computation in OpenFLUX2: the several tunable parameters are introduced for evaluation of achieved convergence of the values

for the bounds as a function of increased *L*. Moreover, if several $CI_{\gamma}^{MC}(u_i)$ bounds for certain fluxes has not reached convergence for the chosen set of control parameters, then the calculation is stopped at the L=$L_{\max}$, and convergence plots of the corresponding CI_{γ}^{MC} bounds as well as corresponding diagrams of optimized fluxes can be visualized on the screen. In this case, user has to make the final decision concerning CI_{γ}^{MC} parameters for these questionable fluxes.

The mentioned automatic convergence control can be applied for several targeted fluxes (e.g., for all free fluxes, or for all fluxes) or for an arbitrary function of free fluxes (net fluxes, flux ratios, *etc.*). Certainly, the number of trials necessary to achieve convergence for flux confidence intervals (and proportionally the total computation time) can be different depending on the chosen target. If the convergence for all free fluxes CI_{γ}^{MC} bounds has been already achieved, then evaluation of CI_{γ}^{MC} for residual dependent fluxes, or for any other functions of free fluxes can be finally continued with the help of MATLAB's FMINCON function. But according to our experience (frankly speaking, not very significant), it is possible to achieve rather satisfactory results concerning evaluation of CI_{γ}^{MC} for the most of questionable fluxes after visualization of the plots when no more than 1,000 – 2,000 trials of Monte Carlo-based approach were performed, without additional exploiting of FMINCON.

It could be added, that when the $\mathbf{U}_i(\widehat{\Theta}_L)$, $i = 1, 2, ..., n,$ estimates acquired by the described Monte Carlo methods are non-symmetrically distributed, the computed $CI_{\gamma}^{MC-1}(\mathbf{U}_i(\widehat{\Theta}_L))$ values are close to the $CI_{\gamma}^{n-lin}(u_i)$ obtained using the

nonlinear-based approach (***1.6.***) [S-4]. Thus, it seems preferable to use the estimations of $CI_{\gamma}^{MC-1}(u_i)$ obtained by the “discarding” strategy in the Monte Carlo-based approach; however, the evaluations provided according to Eq. $(S-1.7.3)$ are also retained in OpenFLUX2.

SF-1 Appendix 1. The special form of the null-space matrix in OpenFLUX(2)

Suppose that the metabolic system consisted of t bi-directional (reversible) reactions and $s = n - 2t$ unidirectional (irreversible) reactions. It is possible to reorder the variables in the initial flux vector $\mathbf{v}$ for generating the following vector:

$$\mathbf{v}' = \begin{pmatrix} \mathbf{v}^{\rightarrow} \\ \mathbf{v}^{irr} \\ \mathbf{v}^{\leftarrow} \end{pmatrix}, \qquad (A1-1)$$

where $\mathbf{v}^{\rightarrow}$ and $\mathbf{v}^{\leftarrow}$ are t-dimensional column vectors consisting of all fluxes corresponded to forward and reverse bi-directional reactions, and $\mathbf{v}^{irr}$ is the s-dimensional column vector corresponding to all irreversible reactions.

The reordering of variables in the flux vector automatically leads to the corresponding reordering of the columns in the $\mathbf{S}$ matrix. Considering Eq. $(S-1.1.1)$, the $\tilde{\mathbf{S}}$ corresponding to the vector $\mathbf{v}'$ could be presented in the following form:

$$\tilde{\mathbf{S}} = \begin{pmatrix} \mathbf{S}^{rev} & \mathbf{S}^{irr} & -\mathbf{S}^{rev} \end{pmatrix}, \qquad (A1-2)$$

where $\mathbf{S}^{rev}$ and $\mathbf{S}^{irr}$ are the matrixes consisting of the columns of $\mathbf{S}$ that correspond to $\mathbf{v}^{\rightarrow}$ and $\mathbf{v}^{irr}$, respectively. Because $\mathrm{rank}(\mathbf{S}) = \mathrm{rank}(\tilde{\mathbf{S}}) = r$, Eq. $(S-1.1.1)$ could be converted to the equivalent equation:

$$\tilde{\mathbf{S}} \cdot \mathbf{v}' = \mathbf{0}. \qquad (A1-3)$$

Let $\mathbf{v}^{\rightarrow,net}$ be a new net forward flux vector that is determined as follows:

$$\mathbf{v}^{\rightarrow,net} = \mathbf{v}^{\rightarrow} - \mathbf{v}^{\leftarrow}, \qquad (A1-4)$$

where, in contrast to other flux vectors that always have their entries ≥ 0, v_i^{net} from $\mathbf{v}^{\rightarrow,net} = (v_1^{net}, v_2^{net}, \ldots, v_t^{net})^{\mathrm{T}}$ could be positive or negative depending on the

scalar values of corresponding $v_i^{\rightarrow}$ forward and $v_i^{\leftarrow}$ reverse fluxes of i-th, $1 \le i \le t$ bi-directional reaction. Then, let a new flux vector, $\mathbf{v}''$, be introduced according to the following equation:

$$\mathbf{v}'' = \begin{pmatrix} \mathbf{v}^{\rightarrow,net} \\ \mathbf{v}^{irr} \\ \mathbf{v}^{\leftarrow} \end{pmatrix}. \qquad (A1-5)$$

The following relation can be detected between $\mathbf{v}'$ and $\mathbf{v}''$:

$$\mathbf{v}' = \begin{pmatrix} \mathbf{v}^{\rightarrow} \\ \mathbf{v}^{irr} \\ \mathbf{v}^{\leftarrow} \end{pmatrix} = \begin{pmatrix} \mathbf{I} & \mathbf{0} & \mathbf{I} \\ \mathbf{0} & \mathbf{I} & \mathbf{0} \\ \mathbf{0} & \mathbf{0} & \mathbf{I} \end{pmatrix} \cdot \begin{pmatrix} \mathbf{v}^{\rightarrow,net} \\ \mathbf{v}^{irr} \\ \mathbf{v}^{\leftarrow} \end{pmatrix} = \mathbf{A} \cdot \mathbf{v}'', \qquad (A1-6)$$

where matrix $\mathbf{A}$ is assigned as follows:

$$\mathbf{A} = \begin{pmatrix} \mathbf{I} & \mathbf{0} & \mathbf{I} \\ \mathbf{0} & \mathbf{I} & \mathbf{0} \\ \mathbf{0} & \mathbf{0} & \mathbf{I} \end{pmatrix}. \qquad (A1-7)$$

Let $\tilde{\mathbf{S}}'$ and $\mathbf{v}'''$ be $\tilde{\mathbf{S}}' = \begin{pmatrix} \mathbf{S}^{rev} & \mathbf{S}^{irr} \end{pmatrix}$ and $\mathbf{v}''' = \begin{pmatrix} \mathbf{v}^{\rightarrow,net} \\ \mathbf{v}^{irr} \end{pmatrix}$, respectively. Then, in accordance with $(A1-2)$ and $(A1-6)$, the following equalities must be accomplished:

$$\tilde{\mathbf{S}} \cdot \mathbf{v}' = \tilde{\mathbf{S}} \cdot (\mathbf{A} \cdot \mathbf{v}'') = (\tilde{\mathbf{S}} \cdot \mathbf{A}) \cdot \mathbf{v}'' = \left(\begin{pmatrix} \mathbf{S}^{rev} & \mathbf{S}^{irr} & -\mathbf{S}^{rev} \end{pmatrix} \cdot \begin{pmatrix} \mathbf{I} & \mathbf{0} & \mathbf{I} \\ \mathbf{0} & \mathbf{I} & \mathbf{0} \\ \mathbf{0} & \mathbf{0} & \mathbf{I} \end{pmatrix} \right) \cdot \mathbf{v}'' =$$

$$= \begin{pmatrix} \mathbf{S}^{rev} & \mathbf{S}^{irr} & \mathbf{0} \end{pmatrix} \cdot \begin{pmatrix} \mathbf{v}^{\rightarrow,net} \\ \mathbf{v}^{irr} \\ \mathbf{v}^{\leftarrow} \end{pmatrix} = \tilde{\mathbf{S}}' \cdot \mathbf{v}''' . \qquad (A1-8)$$

As a result, the mass balance equation $(A1-3)$ is converted to the following equivalent equation:

$$\tilde{\mathbf{S}}' \cdot \mathbf{v}''' = \mathbf{0}, \qquad (A1-9)$$

in relation to the new flux vector, $\mathbf{v}'''$. Because the $\tilde{\mathbf{S}}'$ matrix has been obtained from the $\tilde{\mathbf{S}}$ matrix due to the elimination of the linearly dependent columns, the ranks of the mentioned matrixes are equal.

Notably, if some linearly dependent columns are present in the first t columns of the $\tilde{\mathbf{S}}'$ matrix corresponding to $\mathbf{v}^{\rightarrow,net}$, then the assumed metabolic model includes the cycle consisting of only bi-directional reactions. Certainly, this cycle could be presented as two cycles consisting of only direct and of only reverse fluxes of these bi-directional reactions because the parameters of the fluxes for the cycle consisting of the reverse fluxes potentially have no natural upper limit and do not depend on any parameters of other fluxes. In this case, at least one element from $\mathbf{v}^{\rightarrow,net}$ must be chosen as one of the free fluxes, leading to the assignment of direct and simultaneous reverse fluxes of one bi-directional reaction as free fluxes for this metabolic model. For future calculations, let us consider that there are no such cycles in the assumed model, and therefore, the first t columns of $\tilde{\mathbf{S}}'$ corresponding to $\mathbf{v}^{\rightarrow,net}$ are linearly independent.

A new matrix, $\tilde{\mathbf{S}}''$, could be obtained due to reordering the linearly dependent columns in the right part of the modified matrix and to the Gaussian elimination-mediated conversion of $\tilde{\mathbf{S}}'$ to the following form:

$$\tilde{\mathbf{S}}'' = \begin{pmatrix} \mathbf{I} & \mathbf{0} & \mathbf{M}' \\ \mathbf{0} & \mathbf{I} & \mathbf{M}'' \\ \mathbf{0} & \mathbf{0} & \mathbf{0} \end{pmatrix}, \qquad (A1-10)$$

where $\mathbf{M}'$ and $\mathbf{M}''$ are the matrixes with dimensions $t \times (n-r-t)$ and $(r-t) \times (n-r-t)$, respectively, which have been obtained at the stage of conversion of $\tilde{\mathbf{S}}'$ to $\tilde{\mathbf{S}}''$. In this equation, $\mathbf{v}'''$ is converted to $\hat{\mathbf{v}}'''$ in the following form:

$$\hat{\mathbf{v}}''' = \begin{pmatrix} \mathbf{v}^{\rightarrow,net} \\ \mathbf{v}_{dep}^{irr} \\ \boldsymbol{\theta}^{irr} \end{pmatrix}. \tag{A1-11}$$

Let $\mathbf{M}_{net}^{rev} = -\mathbf{M}'$ and $\mathbf{M}_{net}^{irr} = -\mathbf{M}''$, then from Eq. $(A1-10)$, the new form of the mass balance equation could be expressed as follows:

$$\tilde{\mathbf{S}}'' \cdot \hat{\mathbf{v}}''' = \begin{pmatrix} \mathbf{I} & \mathbf{0} & -\mathbf{M}_{net}^{rev} \\ \mathbf{0} & \mathbf{I} & -\mathbf{M}_{net}^{irr} \\ \mathbf{0} & \mathbf{0} & \mathbf{0} \end{pmatrix} \cdot \begin{pmatrix} \mathbf{v}^{\rightarrow,net} \\ \mathbf{v}_{dep}^{irr} \\ \boldsymbol{\theta}^{irr} \end{pmatrix} = \mathbf{0}, \tag{A1-12}$$

and the following equality would be true:

$$\begin{pmatrix} \mathbf{v}^{\rightarrow,net} \\ \mathbf{v}_{dep}^{irr} \end{pmatrix} = \begin{pmatrix} \mathbf{M}_{net}^{rev} \\ \mathbf{M}_{net}^{irr} \end{pmatrix} \cdot \boldsymbol{\theta}^{irr}. \tag{A1-13}$$

The $\mathbf{N}_{net}$ will be assigned as follows:

$$\mathbf{N}_{net} = \begin{pmatrix} \mathbf{M}_{net}^{rev} \\ \mathbf{M}_{net}^{irr} \\ \mathbf{I} \end{pmatrix}. \tag{A1-14}$$

Then, the general solution of the system $(A1.12)$ could be expressed as follows:

$$\hat{\mathbf{v}}''' = \mathbf{N}_{net} \boldsymbol{\theta}^{irr}. \tag{A1-15}$$

Let $\boldsymbol{\theta} = \begin{pmatrix} \boldsymbol{\theta}^{irr} \\ \mathbf{v}^{\leftarrow} \end{pmatrix}$. Then, according to Eqs. $(A1-5)-(A1-7)$, $(A1-11)$, and $(A1-14)-(A1-15)$, the vector could be returned back to the initial column flux vector expressed in new variables as follows:

$$\mathbf{u}=\begin{pmatrix}\mathbf{v}^{\rightarrow}\\ \mathbf{v}_{dep}^{irr}\\ \boldsymbol{\theta}^{irr}\\ \mathbf{v}^{\leftarrow}\end{pmatrix}=\mathbf{A}\cdot\mathbf{v}''=\mathbf{A}\cdot\begin{pmatrix}\mathbf{v}^{\rightarrow,net}\\ \mathbf{v}_{dep}^{irr}\\ \boldsymbol{\theta}^{irr}\\ \mathbf{v}^{\leftarrow}\end{pmatrix}=\mathbf{A}\cdot\begin{pmatrix}\hat{\mathbf{v}}'''\\ \mathbf{v}^{\leftarrow}\end{pmatrix}=\mathbf{A}\cdot\left(\begin{pmatrix}\mathbf{N}_{net} & \mathbf{0}\\ \mathbf{0} & \mathbf{I}\end{pmatrix}\cdot\begin{pmatrix}\boldsymbol{\theta}^{irr}\\ \mathbf{v}^{\leftarrow}\end{pmatrix}\right)=$$

$$=\left(\begin{pmatrix}\mathbf{I} & \mathbf{0} & \mathbf{0} & \mathbf{I}\\ \mathbf{0} & \mathbf{I} & \mathbf{0} & \mathbf{0}\\ \mathbf{0} & \mathbf{0} & \mathbf{I} & \mathbf{0}\\ \mathbf{0} & \mathbf{0} & \mathbf{0} & \mathbf{I}\end{pmatrix}\cdot\begin{pmatrix}\mathbf{M}_{net}^{rev} & \mathbf{0}\\ \mathbf{M}_{net}^{irr} & \mathbf{0}\\ \mathbf{I} & \mathbf{0}\\ \mathbf{0} & \mathbf{I}\end{pmatrix}\right)\cdot\begin{pmatrix}\boldsymbol{\theta}^{irr}\\ \mathbf{v}^{\leftarrow}\end{pmatrix}=\begin{pmatrix}\mathbf{M}_{net}^{rev} & \mathbf{I}\\ \mathbf{M}_{net}^{irr} & \mathbf{0}\\ \mathbf{I} & \mathbf{0}\\ \mathbf{0} & \mathbf{I}\end{pmatrix}\cdot\boldsymbol{\theta}=\mathbf{N}\cdot\boldsymbol{\theta} \quad . \qquad (A1-16)$$

The bracketed parts of Eq. $(A1-16)$ correspond to Eq. $(S-1.1.8)$ of the main text of ***Supplementary file 1***; therefore, the following relations could be expressed:

$$\mathbf{v}_{dep}^{\rightarrow}=\mathbf{M}_{net}^{rev}\cdot\boldsymbol{\theta}^{irr}+\mathbf{v}^{\leftarrow};\ \mathbf{v}_{dep}^{irr}=\mathbf{M}_{net}^{irr}\cdot\boldsymbol{\theta}^{irr}\ . \qquad (A1-17)$$

SF-1 Appendix 2. Newton's method of the constrained NLLSP solution

In the case of NLLSP for $SSR_{\mathbf{f}}^{SLE}$, the estimation of the optimal $\hat{\boldsymbol{\theta}} \in \Re^{p}$ follows an iterative solution scheme. Starting with random values of free fluxes from the constrained range, the model vector-function $\mathbf{f}$ is evaluated at each g-th iteration, $\boldsymbol{\theta}_g \in \Re^{p}$, to obtain the values of the objective scalar-function as follows:

$$\left(SSR_{\mathbf{f}}^{SLE}\right)_g = SSR_{\mathbf{f}}^{SLE}\left(\boldsymbol{\theta}_g\right) = \frac{1}{2} \cdot [\left(\mathbf{f}\left(\boldsymbol{\theta}_g\right) - \mathbf{m}^{mea}\right)^{\mathrm{T}} \cdot \left(\Sigma_{\mathbf{x}}(\boldsymbol{\sigma}^{mea})\right)^{-1} \cdot \left(\mathbf{f}\left(\boldsymbol{\theta}_g\right) - \mathbf{m}^{mea}\right)], \qquad (A2-1)$$

and to find the optimal vector of free flux changes, $\Delta\boldsymbol{\theta}_g$, which leads to $SSR_{\mathbf{f}}^{SLE}$ minimization, i.e., $\Delta\left(SSR_{\mathbf{f}}^{SLE}\right)_g = \left(SSR_{\mathbf{f}}^{SLE}\right)_{g+1} - \left(SSR_{\mathbf{f}}^{SLE}\right)_g \le 0$. At each consequent g-th iteration, the $SSR_{\mathbf{f}}^{SLE}$ function can be formally expressed around $\boldsymbol{\theta}_g$ using Taylor's expansion:

$$\begin{aligned} SSR_{\mathbf{f}}^{SLE}(\boldsymbol{\theta}) &= SSR_{\mathbf{f}}^{SLE}\left(\boldsymbol{\theta}_g\right) + \sum_{j=1}^{p} \frac{\partial SSR_{\mathbf{f}}^{SLE}}{\partial \theta_j}\left(\boldsymbol{\theta}_g\right) \cdot \left(\theta_j - (\theta_j)_g\right) + \\ &+ \frac{1}{2} \cdot \sum_{j=1}^{p} \sum_{i=1}^{p} \frac{\partial^2 SSR_{\mathbf{f}}^{SLE}}{\partial \theta_j \partial \theta_i}\left(\boldsymbol{\theta}_g\right) \cdot \left(\theta_j - (\theta_j)_g\right) \cdot \left(\theta_i - (\theta_i)_g\right) + O\left((\boldsymbol{\theta} - \boldsymbol{\theta}_g)^3\right) = \qquad , \qquad (A2-2) \\ &= SSR_{\mathbf{f}}^{SLE}\left(\boldsymbol{\theta}_g\right) + [\mathbf{J}_{SSR}\left(\boldsymbol{\theta}_g\right)]^{\mathrm{T}} \cdot \left(\boldsymbol{\theta} - \boldsymbol{\theta}_g\right) + \frac{1}{2} \cdot \left(\boldsymbol{\theta} - \boldsymbol{\theta}_g\right)^{\mathrm{T}} \cdot \mathbf{H}_{SSR}\left(\boldsymbol{\theta}_g\right) \cdot \left(\boldsymbol{\theta} - \boldsymbol{\theta}_g\right) + O\left((\boldsymbol{\theta} - \boldsymbol{\theta}_g)^3\right) \end{aligned}$$

where $\mathbf{J}_{SSR}\left(\boldsymbol{\theta}_g\right)$, with $\dim(\mathbf{J}_{SSR}) = (p \times 1)$, and $\mathbf{H}_{SSR}\left(\boldsymbol{\theta}_g\right)$, with $\dim(\mathbf{H}_{SSR}) = (p \times p)$, are the Jacobian and the Hessian matrixes of the $SSR_{\mathbf{f}}^{SLE}$ scalar-function evaluated at $\boldsymbol{\theta}_g \in \Re^{p}$. Thus, neglecting third and higher order terms of $\Delta\boldsymbol{\theta} = \left(\boldsymbol{\theta} - \boldsymbol{\theta}_g\right)$, $\Delta\left(SSR_{\mathbf{f}}^{SLE}\right)_g$ can be approximated by the following expression:

$$\Delta\left(SSR_{\mathbf{f}}^{SLE}\right)_g \cong [\mathbf{J}_{SSR}\left(\boldsymbol{\theta}_g\right)]^{\mathrm{T}} \cdot \Delta\boldsymbol{\theta} + \frac{1}{2} \cdot \Delta\boldsymbol{\theta}^{\mathrm{T}} \cdot \mathbf{H}_{SSR}\left(\boldsymbol{\theta}_g\right) \cdot \Delta\boldsymbol{\theta}. \qquad (A2-3)$$

Consequently, the minimum of the $\Delta\left(SSR_{\mathbf{f}}^{SLE}\right)_g$ function is achieved when

$$\frac{d\left((\Delta SSR_{\mathbf{f}}^{SLE})_g\right)}{d(\Delta\boldsymbol{\theta})} = \mathbf{J}_{SSR}(\boldsymbol{\theta}_g) + \mathbf{H}_{SSR}(\boldsymbol{\theta}_g)\cdot\Delta\boldsymbol{\theta}_g = \mathbf{0}. \qquad (A2-4)$$

Thus, the $\boldsymbol{\theta}_{g+1} = (\boldsymbol{\theta}_g + \Delta\boldsymbol{\theta}_g) \in \Re^p$ can be found. Indeed, the optimal search direction for the free fluxes at g-th, $g = 1, 2, \ldots$, iteration is as follows:

$$\Delta\boldsymbol{\theta}_g = -[\mathbf{H}_{SSR}(\boldsymbol{\theta}_g)]^{-1}\cdot\mathbf{J}_{SSR}(\boldsymbol{\theta}_g). \qquad (A2-5)$$

In the case of NLLSP for $SSR_{\mathbf{f}}^{PLE}$, which is determined by Eq. $(S-1.3.9)$, analogous calculations lead to the following search direction for the free fluxes:

$$\Delta\boldsymbol{\theta}_g = -[\sum_{i=1}^{k}\mathbf{H}_{SSR_i}(\boldsymbol{\theta}_g)]^{-1}\cdot[\sum_{i=1}^{k}\mathbf{J}_{SSR_i}(\boldsymbol{\theta}_g)], \qquad (A2-6)$$

where the Jacobian and Hessian matrixes, $\mathbf{J}_{SSR_i}(\boldsymbol{\theta}_g)$ and $\mathbf{H}_{SSR_i}(\boldsymbol{\theta}_g)$, respectively, must be calculated for each using the $SSR_{\mathbf{f}_i}^{LE}$ scalar objective functions, $i = 1, 2, \ldots, k$, at $\boldsymbol{\theta} = \boldsymbol{\theta}_g$.

The above-described optimization procedure continues until a predefined convergence criterion is met. Finally, the optimized values of all fluxes, $\hat{\mathbf{u}} = \mathbf{N}\cdot\hat{\boldsymbol{\theta}} \in \Re^n$, are calculated.

Each g-th iteration of the proposed optimization procedure requires the calculation of $\mathbf{J}_{SSR}(\boldsymbol{\theta}_g)$ and $\mathbf{H}_{SSR}(\boldsymbol{\theta}_g)$ matrixes in Eq. $(A2-5)$. The $\mathbf{J}_{SSR}(\boldsymbol{\theta}_g)$ matrix can be analytically calculated in the following way:

$$\mathbf{J}_{SSR}(\boldsymbol{\theta}_g) = \frac{\partial SSR_{\mathbf{f}}^{SLE}}{\partial\boldsymbol{\theta}}(\boldsymbol{\theta}_g) = \mathbf{J}_{\mathbf{f}}(\boldsymbol{\theta}_g)\cdot(\Sigma_{\mathbf{x}})^{-1}\cdot\left(\mathbf{f}(\boldsymbol{\theta}_g) - \mathbf{m}^{mea}\right), \qquad (A2-7)$$

where $\mathbf{J}_{\mathbf{f}}(\boldsymbol{\theta}_g) = \frac{\partial\mathbf{f}}{\partial\boldsymbol{\theta}}(\boldsymbol{\theta}_g)$ is the Jacobian matrix, $\dim(\mathbf{J}_{\mathbf{f}}) = (p\times p)$, of the $\mathbf{f}$ vector-function with respect to $\boldsymbol{\theta}$ evaluated at $\boldsymbol{\theta}_g \in \Re^p$. Notably, this matrix is often called the matrix of the simulated measurement sensitivities or the matrix of sensitivities [S-3, S-4, S-17]. In contrast, the Hessian matrix, $\mathbf{H}_{SSR}(\boldsymbol{\theta}_g)$, cannot

be calculated directly. The following approach is usually applied for the $\mathbf{H}_{SSR}(\boldsymbol{\theta}_g)$ approximated calculation [S-4, S-8]. First, the $\mathbf{f}$ vector-function around $\boldsymbol{\theta}_g$ is formally expressed using Taylor's expansion as follows:

$$\mathbf{f}(\boldsymbol{\theta}) = \mathbf{f}(\boldsymbol{\theta}_g) + [\mathbf{J}_{\mathbf{f}}(\boldsymbol{\theta}_g)]^{\mathrm{T}} \cdot (\boldsymbol{\theta} - \boldsymbol{\theta}_g) + O\left((\boldsymbol{\theta} - \boldsymbol{\theta}_g)^2\right). \qquad (A2-8)$$

Using the value of $\mathbf{f}(\boldsymbol{\theta})$ from Eq. $(A2-8)$, neglecting the second and higher orders of $(\boldsymbol{\theta} - \boldsymbol{\theta}_g)$, and applying Eq. $(A2-7)$, the following expression could be obtained for $\Delta\left(SSR_{\mathbf{f}}^{SLE}\right)_g$:

$$\Delta\left(SSR_{\mathbf{f}}^{SLE}\right)_g \cong [\mathbf{J}_{SSR}(\boldsymbol{\theta}_g)]^{\mathrm{T}} \cdot \Delta\boldsymbol{\theta} + \frac{1}{2} \cdot (\Delta\boldsymbol{\theta})^{\mathrm{T}} \cdot [\mathbf{J}_{\mathbf{f}}(\boldsymbol{\theta}_g)]^{\mathrm{T}} \cdot (\Sigma_{\mathbf{x}})^{-1} \cdot \mathbf{J}_{\mathbf{f}}(\boldsymbol{\theta}_g) \cdot \Delta\boldsymbol{\theta}\,. \qquad (A2-9)$$

From Eqs. $(A2-3)$ and $(A2-9)$ and due to the uniqueness of the form of Taylor's series, the Hessian matrix, $\mathbf{H}_{SSR}(\boldsymbol{\theta}_g)$, can be approximated as follows:

$$\mathbf{H}_{SSR}(\boldsymbol{\theta}_g) \cong [\mathbf{J}_{\mathbf{f}}(\boldsymbol{\theta}_g)]^{\mathrm{T}} \cdot (\Sigma_{\mathbf{x}})^{-1} \cdot \mathbf{J}_{\mathbf{f}}(\boldsymbol{\theta}_g). \qquad (A2-10)$$

Equations analogous to Eq. $(A2-7)$ and $(A2-10)$ can be obtained for computing the $\mathbf{J}_{SSR_i}(\boldsymbol{\theta}_g)$ and $\mathbf{H}_{SSR_i}(\boldsymbol{\theta}_g)$ in Eq. $(A2-6)$ from $\mathbf{f}_i(\boldsymbol{\theta}_g)$, $\mathbf{J}_{\mathbf{f}_i}(\boldsymbol{\theta}_g)$, $(\Sigma_{\mathbf{x}})_i$ and from $\left(\mathbf{m}^{mea}\right)_i$ for all $i = 1, 2, \ldots, k$ labeling experiments from the PLE.

SF-1 Appendix 3. Summing elements in an arbitrary row of the contribution matrix

Let the $\mathbf{C}$ matrix be assigned as follows:

$$\mathbf{C} = \mathbf{N} \cdot [\mathbf{H}_{SSR}(\widehat{\boldsymbol{\theta}})]^{-1} \cdot [\mathbf{J}_{\mathbf{f}}(\widehat{\boldsymbol{\theta}})]^{\mathrm{T}}, \qquad (A3-1),$$

then, Eqs. $(S-1.5.12)$ and $(S-1.5.17)$ can be rewritten in the following shortened forms, respectively:

$$\Sigma_{\hat{\mathbf{u}}} = \mathbf{C} \cdot (\Sigma_{\mathbf{x}})^{-1} \cdot \mathbf{C}^T ; \qquad (A3-2)$$

$$\{\mathbf{CM}\}_{i,j} = \frac{C_{i,j}^2}{\{\Sigma_{\hat{\mathbf{u}}}\}_{i,i} \cdot \{\Sigma_{\mathbf{x}}\}_{j,j}} . \qquad (A3-3)$$

Because the $\Sigma_{\mathbf{x}}$ matrix is considered diagonal (Eq. $(S=1.3.3)$), the inverse $(\Sigma_{\mathbf{x}})^{-1}$ is also a diagonal matrix with diagonal elements given by the following equation:

$$\{(\Sigma_{\mathbf{x}})^{-1}\}_{j,j} = \frac{1}{\{\Sigma_{\mathbf{x}}\}_{j,j}} = \left(\{\Sigma_{\mathbf{x}}\}_{j,j}\right)^{-1} . \qquad (A3-4)$$

Then, for an arbitrary i, the following equation is derived from Eqs. $(A3-2)$ and $(A3-4)$:

$$\{\Sigma_{\hat{\mathbf{u}}}\}_{i,i} = \sum_{k=1}^{w} C_{i,k} \cdot \left(\sum_{t=1}^{w} \{(\Sigma_{\mathbf{x}})^{-1}\}_{k,t} \cdot C_{t,i}^{\mathrm{T}} \right) = \sum_{k=1}^{w} C_{i,k} \cdot \{(\Sigma_{\mathbf{x}})^{-1}\}_{k,k} \cdot C_{k,i}^{\mathrm{T}} = \sum_{k=1}^{w} C_{i,k}^2 \cdot \left(\{\Sigma_{\mathbf{x}}\}_{k,k}\right)^{-1} . \qquad (A3-5)$$

Thus, the sum of all elements in the arbitrary i-th row of the $\mathbf{CM}$ matrix can be calculated using Eqs. $(A3-3)$, $(A3-4)$, and $(A3-5)$ as follows:

$$\sum_{j=1}^{w} \{\mathbf{CM}\}_{i,j} = \sum_{j=1}^{w} \frac{C_{i,j}^2}{\{\Sigma_{\hat{\mathbf{u}}}\}_{i,i} \cdot \{\Sigma_{\mathbf{x}}\}_{j,j}} = \sum_{j=1}^{w} \frac{C_{i,j}^2 \cdot \left(\{\Sigma_{\mathbf{x}}\}_{j,j}\right)^{-1}}{\sum_{k=1}^{w} C_{i,k}^2 \cdot \left(\{\Sigma_{\mathbf{x}}\}_{k,k}\right)^{-1}} = \frac{\sum_{j=1}^{w} C_{i,j}^2 \cdot \left(\{\Sigma_{\mathbf{x}}\}_{j,j}\right)^{-1}}{\sum_{k=1}^{w} C_{i,k}^2 \cdot \left(\{\Sigma_{\mathbf{x}}\}_{k,k}\right)^{-1}} = 1 . \qquad (A3-6)$$

References cited in Supplementary File 1

S-1. Wiechert W: **^{13}C metabolic flux analysis.** *Metab Eng* 2001, **3**:195-206.

S-2. Varma A, Palsson BO: **Metabolic flux balancing: basic concepts, scientific and practical use.** *Bio/Technology* 1994, **12:**994-998.

S-3. Yang TH: **^{13}C-based metabolic flux analysis: fundamentals and practice.** *Methods Mol Biol* 2013, **985:**297-334.

S-4. Antoniewicz MR, Kelleher JK, Stephanopoulos G: **Determination of confidence intervals of metabolic fluxes estimated from stable isotope measurements.** *Metab Eng* 2006, **8:**324-337.

S-5. Foster LV: **Rank and null space calculations using matrix decomposition without column interchanges.** *Linear Algebra Appl* 1986, **74**:47-71.

S-6. Quek LE, Wittmann C, Nielsen LK, Krömer JO: **OpenFLUX: efficient modelling software for ^{13}C-based metabolic flux analysis.** *Microb Cell Fact* 2009, **8:**25.

S-7. Wiechert W, Möllney M, Petersen S, de Graaf AA: **A universal framework for ^{13}C metabolic flux analysis.** *Metab Eng* 2001, **3:**265-283.

S-8. Yang TH, Frick O, Heinzle E: **Hybrid optimization for ^{13}C metabolic flux analysis using systems parametrized by compactification.** *BMC Systems Biol* 2008, **2:**29.

S-9. Wiechert W, de Graaf AA: **Bidirectional reaction steps in metabolic networks: I. Modeling and stimulation of carbon isotope labeling experiments.** *Biotechnol Bioeng* 1997, **55:**101-117.

S-10. Möllney M, Wiechert W, Kownatzki D, de Graaf AA: **Bidirectional reaction steps in metabolic networks: IV. Optimal design of isotopomer labeling experiments.** *Biotechnol Bioeng* 1999, **66:**86-103.

S-11. Wang NS, Stephanopoulos G: **Application of macroscopic balances to the identification of gross measurement errors.** *Biotechnol Bioengin* 1983, **25:**2177-2208.

S-12. Yang TH, Bolten CJ, Coppi MV, Sun J, Heinzle E: **Numerical bias estimation for mass spectrometric mass isotopomer analysis.** *Anal Biochem* 2009, **388:**192-203.

S-13. Antoniewicz MR, Kelleher JK, Stephanopoulos G: **Elementary metabolite units (EMU): a novel framework for modeling isotopic distributions.** *Metab Eng* 2007, **9:**68-86.

S-14. Schmidt K, Carlsen M, Nielsen J, Villadsen J: **Modeling isotopomer distributions in biochemical networks using isotopomer mapping matrices.** *Biotechnol Bioeng* 1997, **55:**931-840.

S-15. Byrd RH, Hribar ME, Nocedal J: **An interior point algorithm for large scale nonlinear programming.** *SIAM J Optim* 1999, **9**: 877-900.

S-16. Wittmann C, Heinzle E: **Genealogy profiling through strain improvement by using metabolic network analysis: metabolic flux genealogy of several generations of lysine-producing corynebacteria.** *Appl Environ Microbiol* 2002, **68:**5843-5859.

S-17. Wittmann C, Kim HM, Heinzle E: **Metabolic network analysis of lysine producing *Corynebacterium glutamicum* at a miniaturized scale.** *Biotechnol Bioeng* 2004, **87:**1-6.

S-18. Leighty RW, Antoniewicz MR: **COMPLETE-MFA: Complementary parallel labeling experiments technique for metabolic flux analysis.** *Metab Eng* 2013, **20**:49-55.

S-19. Bryk SW, Raudenbush AS: *Hierarchical linear models: applications and data analysis methods*. 2nd edition. Thousand Oaks, CA [u.a.]: Sage Publications; 2002.

S-20. Wiechert W, Siefke C, de Graaf AA, Mark A: **Bidirectional reaction steps in metabolic networks: II. Flux estimation and statistical analysis.** *Biotechnol Bioeng* 1997, **55:**118-135.

S-21. Nocedal J, Wright SJ: *Numerical optimization.* 2nd edition. Berlin, New York: Springer-Verlag; 2006.

S-22. Suthers PF, Burgard AP, Dasika MS, Nowroozi F, van Dien S, Keasling JD, Maranas CD: **Metabolic flux elucidation for large-scale models using ^{13}C labeled isotopes.** *Metab Eng* 2007, **9:**387-405.

S-23. Antoniewicz MR: **Using multiple tracers for ^{13}C metabolic flux analysis.** *Methods Mol Biol* 2013, **985**:353-365.

S-24. Khoshgoftaar TM, Allen EB: **Controlling overfitting in classification-tree models of software quality.** *Empirical Software Engineering* 2001, **6:**59-79.

S-25. Hartmann WM, Hartwig RE: **Computing the Moore-Penrose inverse for the covariance matrix in constrained nonlinear estimation.** *SIAM J Optim* 1996, **6:**727-747.

S-26. Pázman A: *Foundations of optimum experimental design.* Dordrecht, The Netherlands: Kluwer Academic Publishing; 1986.

S-27. He L, Xiao Y, Gebreselassie N, Zhang F, Antoniewicz MR, Tang YJ, Peng L: **Central metabolic responses to the overproduction of fatty acids in *Escherichia coli* based on ^{13}C-metabolic flux analysis.** *Biotechnol Bioeng* 2013, **111:**575-85.
