# Supplementary material for: OpenFLUX2: 13C-MFA modeling software package adjusted for the comprehensive analysis of single and parallel labeling experiments
Source: Microb Cell Fact. 2014 Nov 19;13:152. doi: 10.1186/s12934-014-0152-x (PMC4263107; doi:10.1186/s12934-014-0152-x)
Supplement: Additional file 2: — Additional figures illustrated results of performed in silico LEs. The PDF-format file contains additional illustrations of the in silico experimental results, denoted as Figure SF-2.x in the text. [file 12934_2014_152_MOESM2_ESM.pdf]

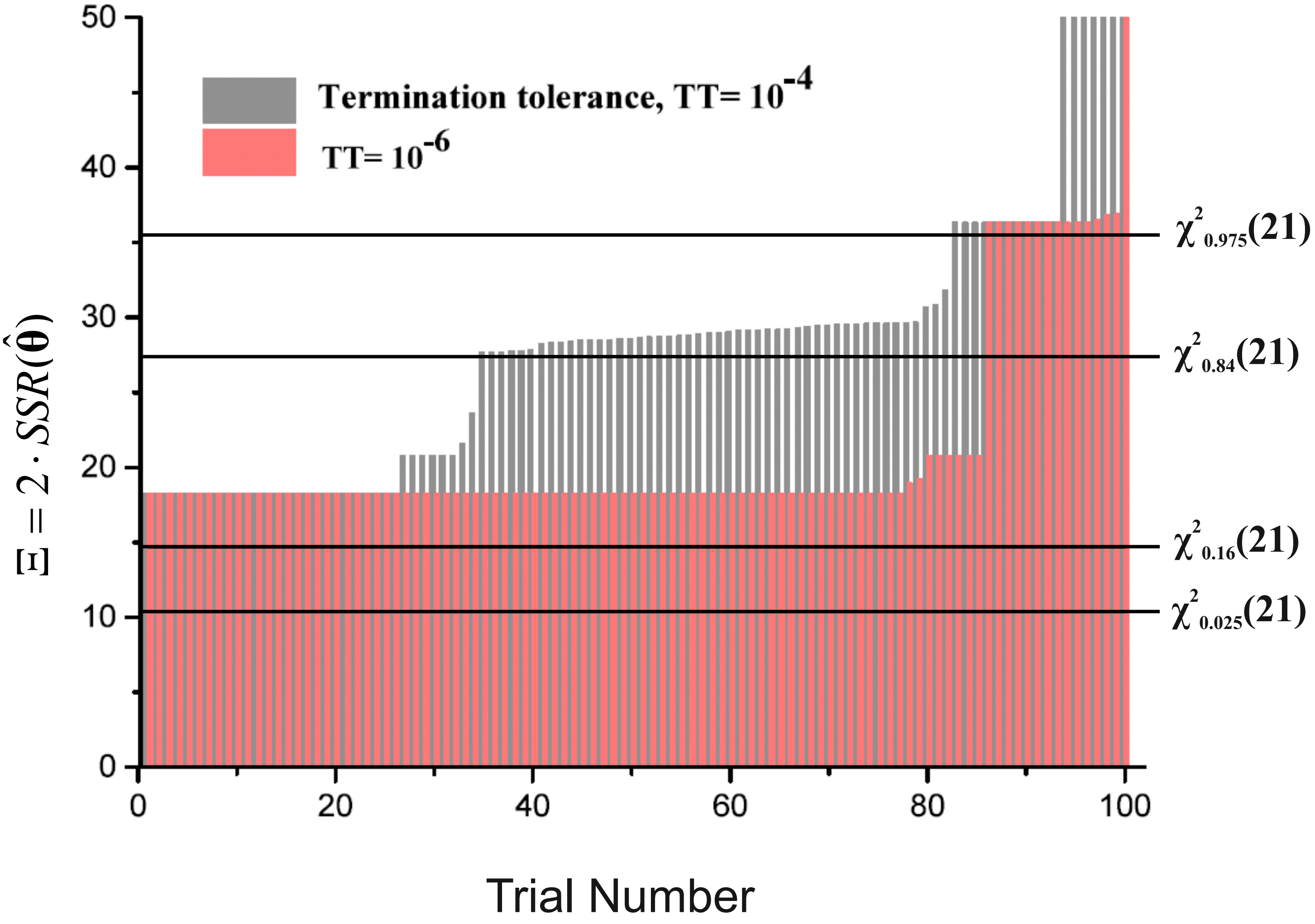

Figure SF-2.1 The spread of the $\Xi_k = \Xi(\widehat{\boldsymbol{\theta}}_k)$ **objective function values obtained in** $k = 1, 2, \ldots, 100$ **trials** of fitting procedures initiated each from an arbitrary set of initial free fluxes, $\boldsymbol{\theta}_k \in \Re^p$, and provided with default value of TT=10^{-4} **(A)**, or with higher accuracy, TT=10^{-6} **(B)**. The critical values of the χ^2-distribution function, $\chi^2_{\alpha/2}$ and $\chi^2_{1-\alpha/2}$, with degrees of freedom equal to 21 (see the main text) at the significance level of $\alpha = 0.05$ and $\alpha = 0.32$, respectively, are indicated.

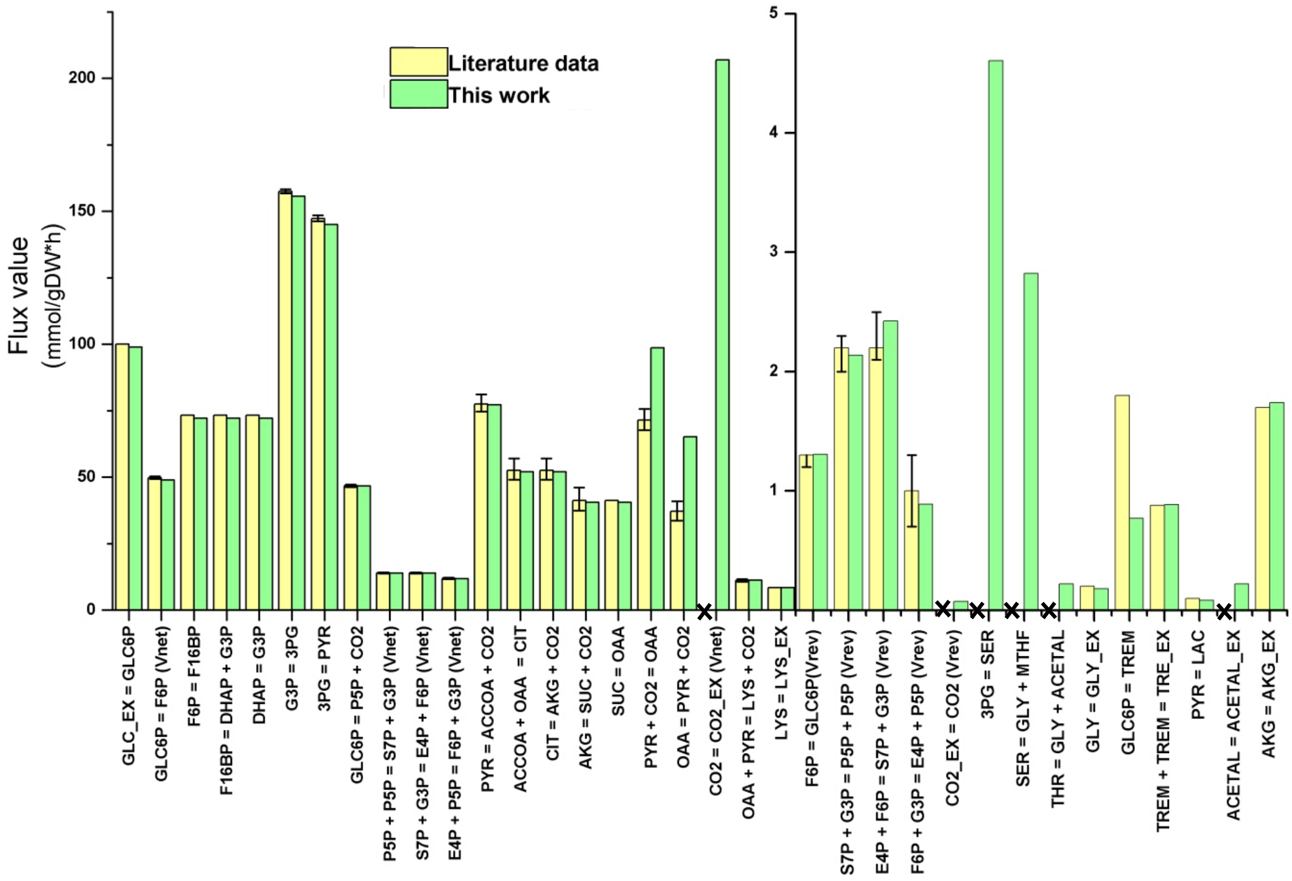

Figure SF-2.2. Comparison of the flux estimations for the models of central carbon metabolism of lysine-producing *C. glutamicum* developed previously [68] and slightly modified in the present study. Reactions related to biomass synthesis were different in these models and did not compare accordingly. The black crosses indicate fluxes in which estimations were not available from the literature. Error bars indicate a 90% confidence interval (if available) for the literature data. For better apparency, the novel and earlier computed parameters of fluxes were comparatively presented not in the form of $v^{\rightarrow}$ and $v^{\leftarrow}$ for direct and reverse components of bi-directional reaction, as considered in the present study, but in the form of $v_{net} = \left| v^{\rightarrow} - v^{\leftarrow} \right|$ and flux reversibility, $v_{rev} = v^{\leftarrow} / v^{net}$, as used in [68].

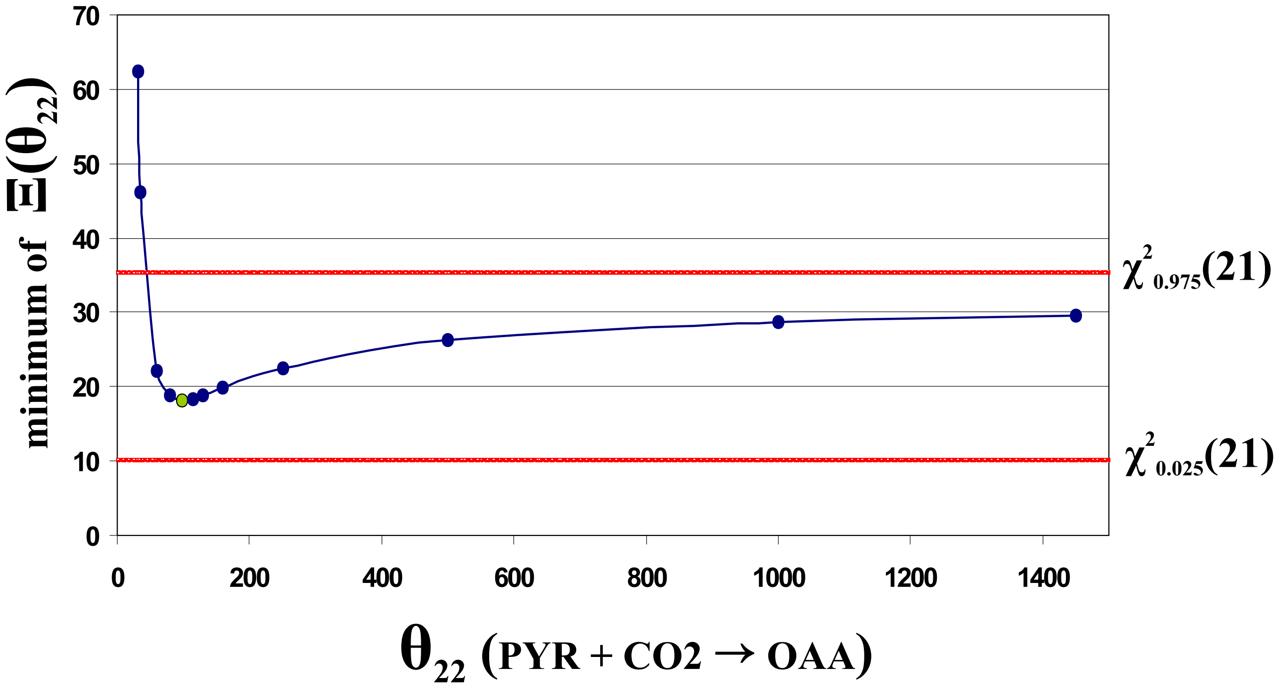

Figure SF-2.3 Dependence of the doubled minimized sum of squared residuals, i.e., $\Xi(\theta_{22}) = 2 \times \min_{\substack{\theta_{22}=const \\ \theta_j \in \Re^P, \forall j \neq 22}} SSR(\boldsymbol{\theta})$**, on the value of** θ_{22} **flux.** The flux θ_{22} was sequentially equal to a predefined value (i.e., $\theta_{22} = const = (\theta_{22})_{true} + \Delta(\theta_{22})$) from its feasible constrained domain, followed by minimizing the $\underset{\theta_j \in \Re^P, \forall j \neq 22}{SSR(\boldsymbol{\theta})}$. A green point indicates the found global minimum, i.e., $\Xi(\theta_{22} = (\theta_{22})_{true})$. The range of the χ^2-statistically acceptable $\Xi(\boldsymbol{\theta})$ values is bounded by red lines, which corresponds to minimal, $\chi^2_{0.025}(21)$, and maximal, $\chi^2_{0.975}(21)$, critical values of χ^2-distribution at significance level of 0.05 and degrees of freedom equal to 21.

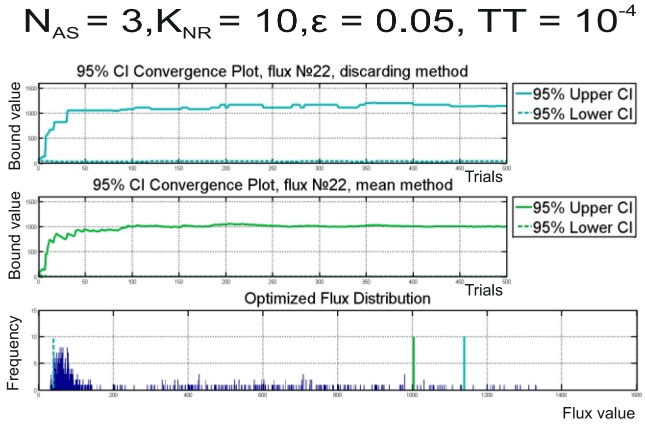

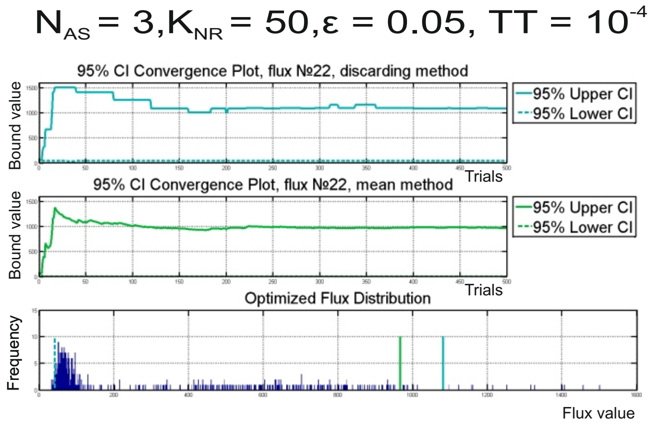

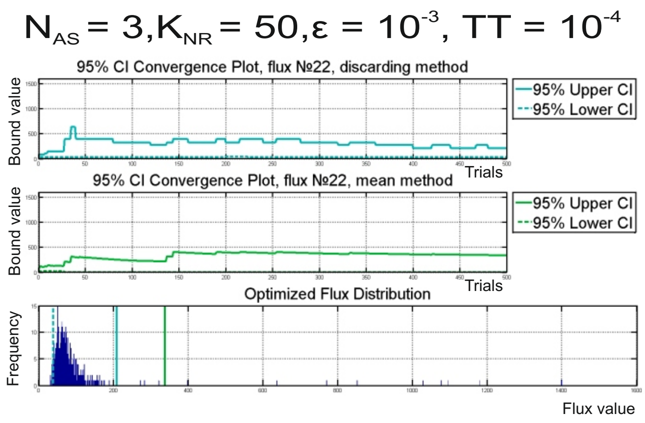

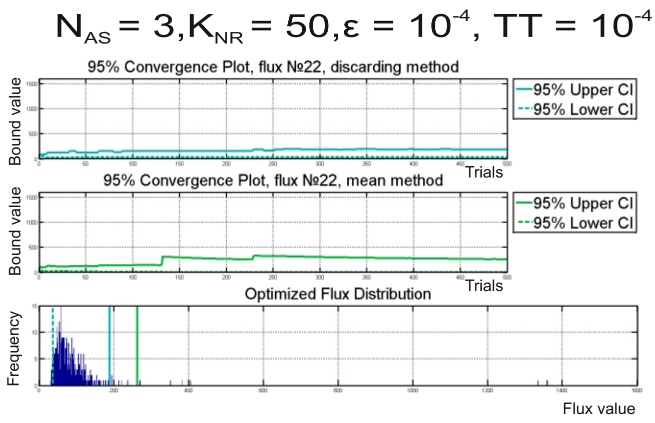

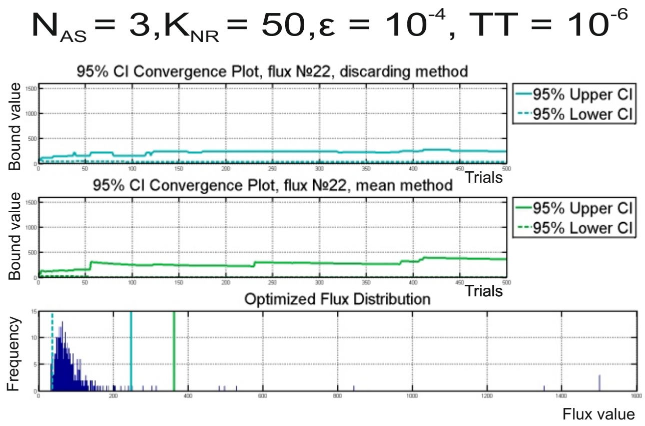

Figure SF-2.4 Confidence interval determination under different settings of Monte-Carlo control parameters. Following control parameters were varied: K_{NR} – maximal number of runs in each trial; ε – proximity threshold and TT – termination tolerance. Most essential effect was observed at the stage of ε decrease.

The number of performed trial was 500 in each case. Convergence plots and histogram were obtained by OpenFLUX2 visualization tool.

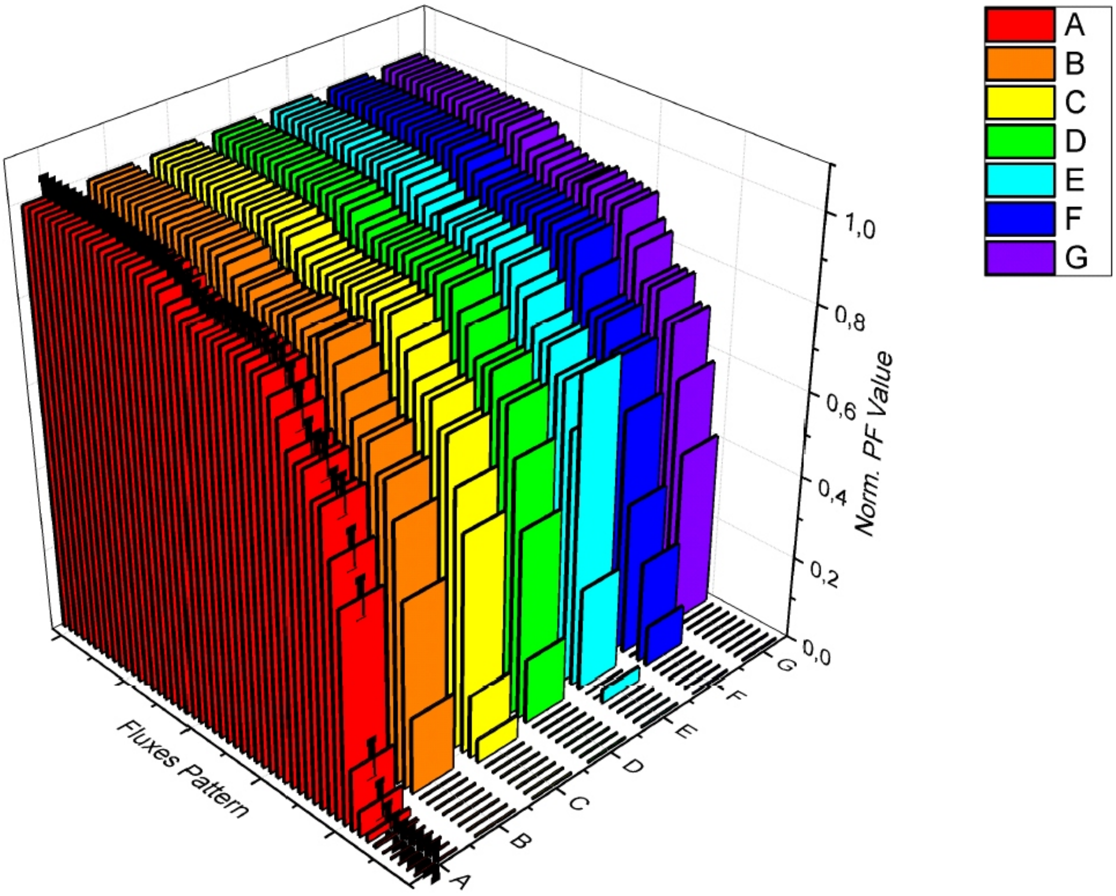

Figure SF-2.5 The values of normalized flux precision functions in dependence on fluxes computed for differently corrupted experimental data for SLEs with [1-^{13}C]-glucose as a tracer. The u_i flux positions were reordered in accordance with the increased values of $\eta_{0.95}^{MC-1}(u_i)$ for SLE with uncorrupted experimental data (row **G**). Row **A** presents the results obtained for the mean values of $\eta(u_i)$ functions, with an error bar indicated by the doubled $\sigma_{\eta(0.95)}(u_i)$ obtained for five independent SLEs with differently corrupted experimental data (rows **B - F**).

Figure SF-2.6 Identification of optimal [1-^{13}C]/[U-^{12}C]/[U-^{13}C]-glucose mixtures by experimental design studies of *D*-factor minimization **(A)** "general optimization", and minimization of linear approximation of the following free flux variances ("partial optimization"): **(B)** θ_{10} ; **(C)** θ_{12} ; **(D)** θ_{14} ; **(E)** θ_{22} ; and **(F)** θ_{31} . Positions corresponding to the optimized content of the mixed tracers are indicated by crisscrosses. Ternary plots were generated by OriginPro 9.1 software.

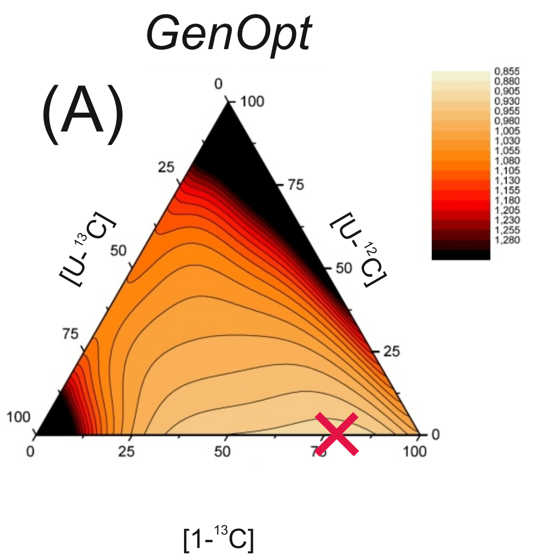

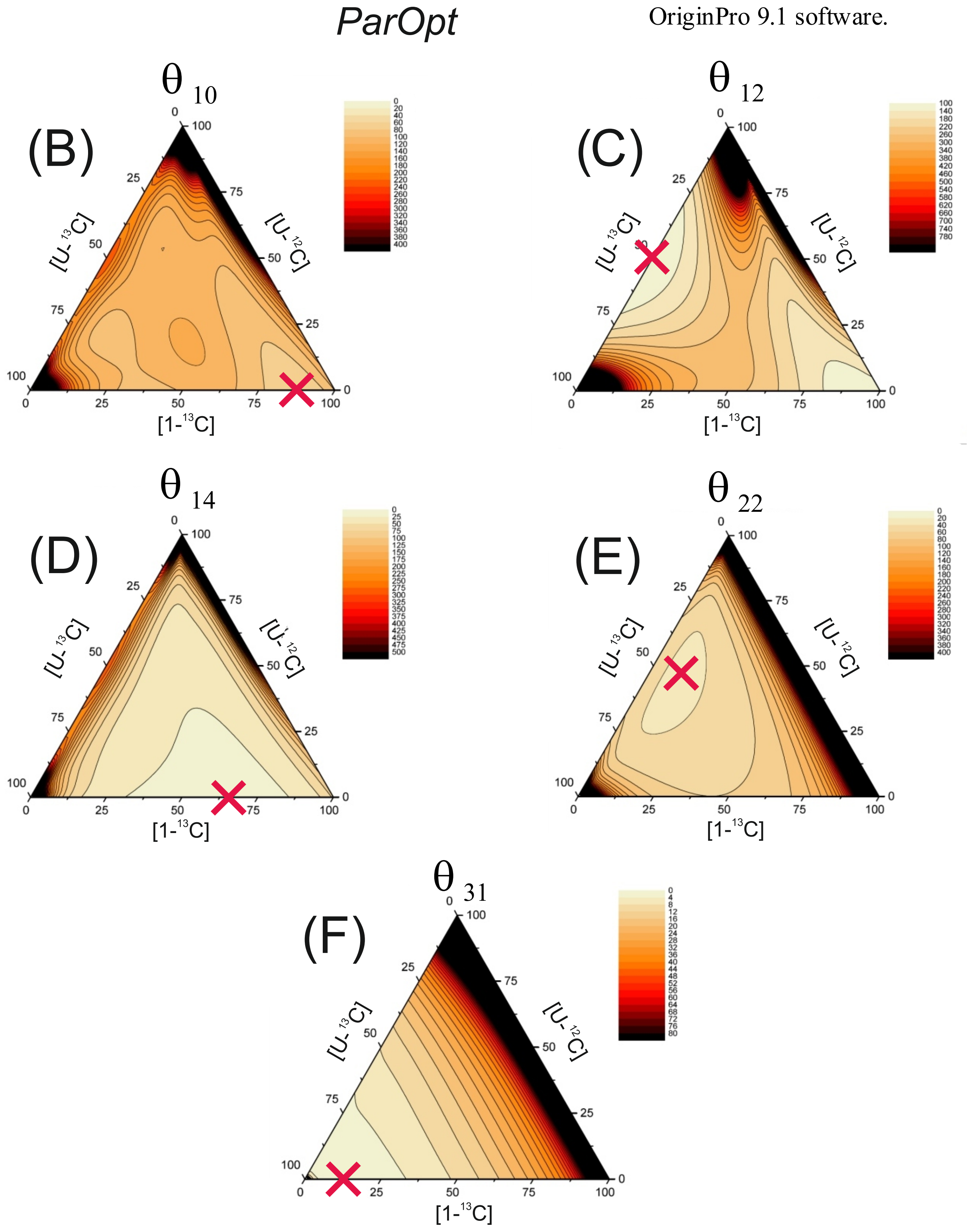

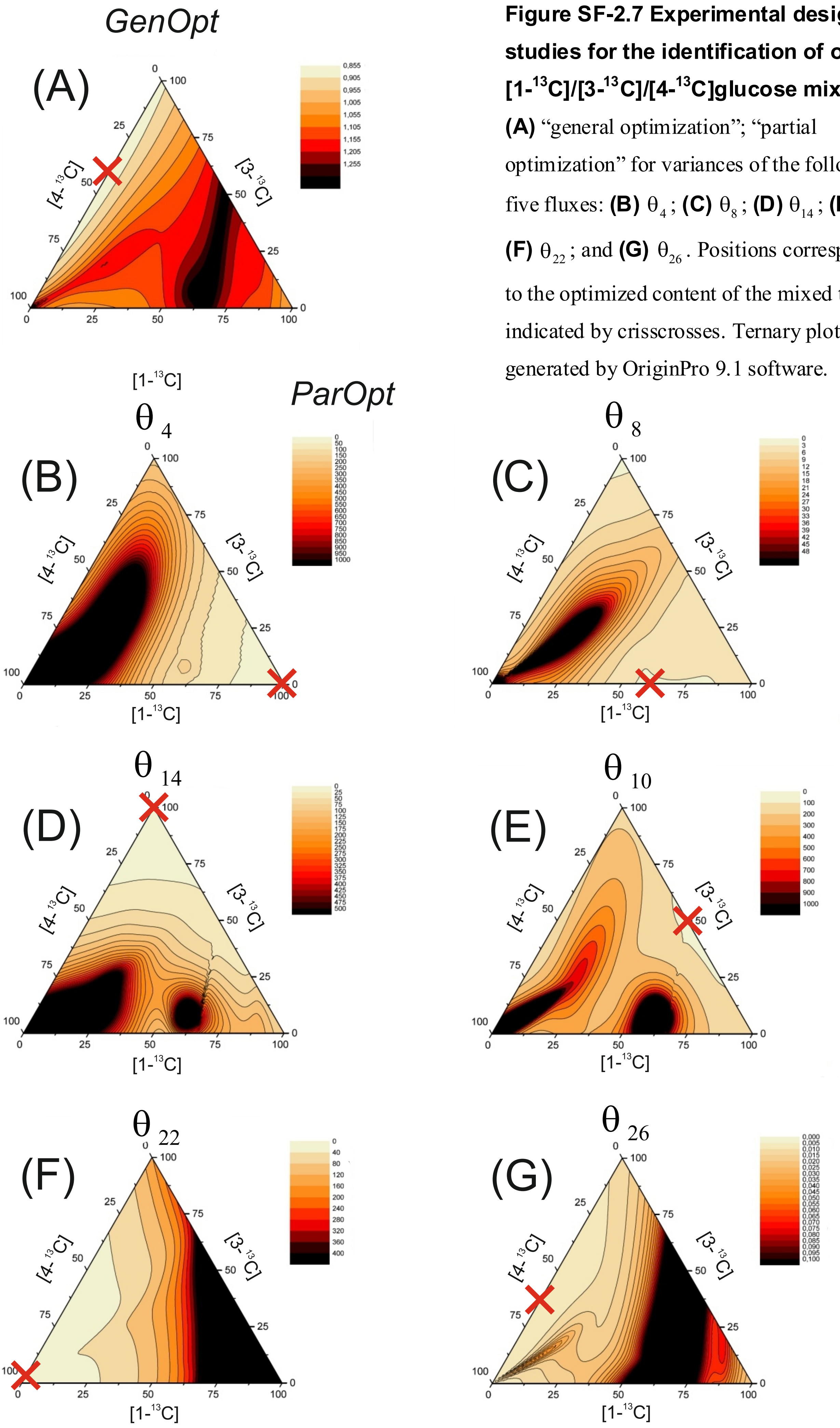

Figure SF-2.7 Experimental design studies for the identification of optimal [1-^{13}C]/[3-^{13}C]/[4-^{13}C]glucose mixtures. **(A)** "general optimization"; "partial optimization" for variances of the following five fluxes: **(B)** θ_4; **(C)** θ_8; **(D)** θ_{14}; **(E)** θ_{10}; **(F)** θ_{22}; and **(G)** θ_{26}. Positions corresponding to the optimized content of the mixed tracers are indicated by crisscrosses. Ternary plots were generated by OriginPro 9.1 software.
